# Comparative genomics of metabolic capacities of regulons controlled by *cis*-regulatory RNA motifs in bacteria

**DOI:** 10.1186/1471-2164-14-597

**Published:** 2013-09-02

**Authors:** Eric I Sun, Semen A Leyn, Marat D Kazanov, Milton H Saier, Pavel S Novichkov, Dmitry A Rodionov

**Affiliations:** 1 Department of Molecular Biology, Division of Biological Sciences, University of California at San Diego, 92093 La Jolla, CA, USA; 2 Sanford-Burnham Medical Research Institute, 92037 La Jolla, CA, USA; 3 A.A. Kharkevich Institute for Information Transmission Problems, Russian Academy of Sciences, 127994 Moscow, Russia; 4 Lawrence Berkeley National Laboratory, 94710 Berkeley, CA, USA

**Keywords:** RNA regulatory motif, Riboswitch, Regulon, Gene function, Comparative genomics, Bacteria

## Abstract

**Background:**

*In silico* comparative genomics approaches have been efficiently used for functional prediction and reconstruction of metabolic and regulatory networks. Riboswitches are metabolite-sensing structures often found in bacterial mRNA leaders controlling gene expression on transcriptional or translational levels.

An increasing number of riboswitches and other *cis*-regulatory RNAs have been recently classified into numerous RNA families in the Rfam database. High conservation of these RNA motifs provides a unique advantage for their genomic identification and comparative analysis.

**Results:**

A comparative genomics approach implemented in the RegPredict tool was used for reconstruction and functional annotation of regulons controlled by RNAs from 43 Rfam families in diverse taxonomic groups of Bacteria. The inferred regulons include ~5200 *cis*-regulatory RNAs and more than 12000 target genes in 255 microbial genomes. All predicted RNA-regulated genes were classified into specific and overall functional categories. Analysis of taxonomic distribution of these categories allowed us to establish major functional preferences for each analyzed *cis*-regulatory RNA motif family. Overall, most RNA motif regulons showed predictable functional content in accordance with their experimentally established effector ligands. Our results suggest that some RNA motifs (including thiamin pyrophosphate and cobalamin riboswitches that control the cofactor metabolism) are widespread and likely originated from the last common ancestor of all bacteria. However, many more analyzed RNA motifs are restricted to a narrow taxonomic group of bacteria and likely represent more recent evolutionary innovations.

**Conclusions:**

The reconstructed regulatory networks for major known RNA motifs substantially expand the existing knowledge of transcriptional regulation in bacteria. The inferred regulons can be used for genetic experiments, functional annotations of genes, metabolic reconstruction and evolutionary analysis. The obtained genome-wide collection of reference RNA motif regulons is available in the RegPrecise database (http://regprecise.lbl.gov/).

## Background

Riboswitches are found mostly in bacterial genomes and represent genetic elements that frequently reside at the leader regions of mRNAs. Riboswitches achieve gene regulation by possessing two alternative structural states and are likened to an “on/off” switch [1]. Direct binding of a metabolic or metal ion ligand to the aptamer domain in a riboswicth causes its conformation to change and allows regulated expression of downstream genes on either the transcriptional or the translational level [2,3]. The regulatory mechanisms for riboswitch-dependent gene control are based on the formation of alternative RNA structures that causes premature termination of transcription, inhibition of translation initiation or attenuation of mRNA stability [4,5]. Additionally, there are riboswitches that behave like ribozymes that self-cleave when a specific conformation is achieved [6], and riboswitches that situate in intron gene regions and modulate RNA splicing in eukaryotes [7]. As there is usually no requirement for a protein factor to mediate ligand recognition, it has been proposed that riboswitch elements represent an ancient gene regulatory mechanism that had arisen prior to the rise of protein/peptides [2,4,8].

An increasing number of riboswitches and other *cis*-acting RNA regulatory motifs have recently been identified using a combination of comparative genomics and experimental approaches [2,4,9,10]. At present, more than two dozen of different classes of riboswitches have been experimentally characterized. Many of these functionally known riboswitches operate as sensors for key intracellular metabolites including co-enzymes, amino acids and nucleotides, and they thus controls the key biosynthetic pathways in the bacterial cell [11]. Several other riboswitches such as the M-box and the magnesium sensor control metal homeostasis [3,12]. A special class of *cis*-regulatory RNA elements called T-boxes is utilized for the control of transcription of amino acid metabolic genes in *Bacillus subtilis* and other Gram-positive bacteria [13-15]. T-boxes sense the amino acid availability in the cell by a unique mechanism when an uncharged tRNA specifically recognizes the tRNA anticodon within the conserved structure of this RNA [16]. In contrast, *Escherichia coli* and related Gram-negative bacteria utilize a different RNA-based mechanism of transcription attenuation for the control of amino acid biosynthesis operons, which involves translation of a portion of the leader sequence that codes for a specific amino acid-rich peptide [17]. Finally, many ribosomal protein operons are controlled by unique leader mRNA structures both in Gram-negative and Gram-positive bacteria [18,19].

Many classes of widespread riboswitches controlling coenzyme and amino acid metabolism have been analyzed in completely sequenced organisms by comparative genomics resulting in *in silico* reconstruction of their respective regulons [13-15,20-24]. A high level of conservation among riboswitch sequences and secondary structures is useful for their computational identification in genomic sequences. Representatives of more than 40 riboswitches and other *cis*-regulatory RNA motifs identified in bacterial genomes are available in the Rfam database [25], where they are grouped into unique RNA families. Several computational tools, such as RibEx [26] and RiboSW [27], were previously implemented allowing the search of known riboswithces in any input nucleotide sequence. Identification of cognate ligands for yet uncharacterized candidate riboswitches is a challenging task, where genome context analyses of riboswicthes and identification of putative regulon members can help predict novel ligands [28]. On the other hand, the comprehensive analysis of the distribution of diverse riboswitches will offer a glimpse into the putative ancestral RNA world and the evolution of gene regulatory mechanisms. The previous analyses of the overall distribution of riboswitches in sequenced genomes demonstrated their abundance in complete genomes of Bacteria from major bacterial phyla (such as Firmicutes and Proteobacteria), as well as in environmental microbial sequences [29,30]. However, with the exception of the thiamin pyrophosphate (TPP) sensing riboswitch, all other widely distributed in bacteria riboswitches were not found in Archaea and Eukaryotes, suggesting two alternative evolutionary scenarios: (i) their origin in the last common ancestor of Bacteria, or (ii) their early origin perhaps from the RNA World and their subsequent loss in archaeal and eukaryotic kingdoms [2].

The comparative genomic analysis of microbial regulons is an efficient method for functional gene annotation and metabolic pathway reconstruction, especially when combined with traditional approaches of genome context analysis [31]. As most riboswitch domains are highly conserved, many genes with previously unknown functions or ligand specificities can be reliably annotated [32]. For example, the systematic genomic analysis of coenzyme-specific riboswitch regulons has resulted in identification of the unusual Energy-Coupling Factor (ECF) class of modular metabolic transporters involved in the uptake of essential vitamins [33]. Previous efforts on riboswitch analysis in microbial communities from diverse environmental sources had revealed a high abundance of certain coenzyme and amino acid specific riboswitches in metagenomes, allowing identification of new riboswicth-regulated functions such as phosphoserine aminotransferase and malate synthase under the regulation of the glycine riboswitch [34]. Although environmental sequencing can give us clues about the relative abundances of known riboswitches as well as allow discovery of novel putative riboswitches, it is only through careful integration of complete genomes that a meaningful picture of metabolic networks and their probable evolutionary pathways will emerge.

In this study, we combine genome context analysis with *cis*-regulatory RNA motif detection to elucidate the metabolic capacities of RNA-mediated gene regulation in bacteria. We combined the inference of regulatory RNAs and their operon analysis in completely sequenced bacterial genomes to evaluate the regulatory networks for different Rfam families of *cis*-regulatory RNA motifs. As a result, 43 RNA families including all known and candidate riboswitches, the ribosomal and amino acid operon leaders and T-boxes were analyzed in the respresentative subset of genomes from 24 taxonomic groups of Bacteria. Our results show that relatively few RNA motifs originated in the last common ancestor of Bacteria and bring into question the notion that the riboswitch is a remnant of the RNA world. Instead, many RNA motifs probably arose recently as they are restricted to a few closely related taxonomic groups. Functional analysis of reconstructed RNA motif regulons in diverse taxonomic lineages of bacteria revealed the lineage-specific metabolic gene content preferences and identified major trends in the evolution of their regulons. A large number of regulatory interactions from the inferred RNA motif regulons contribute to genome-wide transcriptional regulatory networks and metabolic models of bacterial cells.

## Results

In this work, we analyzed 43 described families of bacterial *cis*-regulatory RNA motifs that are currently available in the Rfam database [35] (Table 1). These include 21 known or 10 predicted metabolite-sensing riboswitches, the PyrR binding site motif, the T-box motif and ten families of *cis*-regulatory RNA motifs termed ‘leaders’ that reside in the leader regions of ribosomal and amino acid biosynthesis operons. For the comparative analysis of these RNA motif regulons, we selected a set of 255 representative genomes from 24 diverse taxonomic lineages of Bacteria (Additional file 1). Among the analyzed lineages, there are five taxonomic groups of Firmicutes and eleven groups of Proteobacteria. Within each lineage, we selected a representative subset of genomes that excludes closely-related strains and species and which is most suitable for comparative analysis using the RegPredict tool [36]. The obtained list of genomes for comparative analysis includes representatives of major bacteria phyla for which complete genomes of at least four diverse species are available (Table 2).

**Table 1:** Functional overview of regulatory RNA families included in this study

**CC**	**RNA motif name**	**Effector (for riboswitches)**	**No**	**Experimental**	**Rfam ID**
**A**	TPP (*THI* element)	thiamin pyrophosphate	564	known	RF00059
**A**	Cobalamin (*B12* element)	adenosylcobalamin	536	known	RF00174
**A**	Glycine	glycine	324	known	RF00504
**A**	yybP-ykoY	?	232	predicted	RF00080
**A**	FMN (*RFN* element)	flavin mononucleotide	233	known	RF00050
**B**	SAM (S-box)	S-adenosylmethionine	257	known	RF00162
**B**	pyrR	-	211	known	RF00515
**B**	Lysine (L-box)	lysine	186	known	RF00168
**B**	Purine (G-box)	guanine, adenine	141	known	RF00167
**B**	GEMM	cyclic di-GMP	89	known	RF01051
**B**	PreQ1	pre-queuosine_1_	72	known	RF00522
**B**	MOCO	molybdenum or tungsten cofactor?	62	known	RF01055
**B**	ydaO-yuaA	ATP	59	known	RF00379
**B**	ykkC-yxkD	?	58	predicted	RF00442
**B**	mini-ykkC	?	67	predicted	RF01068
**B**	ykoK (M-box)	magnesium	48	known	RF00380
**B**	glmS	glucosamine-6-phosphate	44	known	RF00234
**B**	SAH	S-adenosylhomocysteine	27	known	RF01057
**C**	SAM-Alpha	S-adenosylmethionine?	39	known	RF00521
**C**	serC	?	32	predicted	RF00517
**C**	THF	tetrahydrofolate	23	known	RF01831
**C**	PreQ1-II	pre-queuosine_1_	17	known	RF01054
**C**	sucA	?	14	predicted	RF01070
**C**	glnA	glutamine	13	known	RF01739
**C**	SAM-IV	S-adenosylmethionine	13	known	RF00634
**C**	SAM-SAH	S-adenosylmethionine, S-adenosylhomocysteine	13	known	RF01727
**C**	Smk-box	S-adenosylmethionine	13	known	RF01767
**C**	speF	?	12	predicted	RF00518
**C**	SAM-Chlorobi	S-adenosylmethionine	11	predicted	RF01724
**C**	ylbH	?	10	predicted	RF00516
**C**	ybhL	?	8	predicted	RF00520
**C**	Mg sensor	magnesium	5	known	RF01056
**D**	L10 leader	-	108	predicted	RF00557
**D**	S15 leader	-	98	known	RF00114
**D**	L20 leader	-	75	predicted	RF00558
**D**	L21 leader	-	67	predicted	RF00559
**D**	L19 leader	-	61	predicted	RF00556
**D**	L13 leader	-	43	predicted	RF00555
**E**	His leader	-	49	known	RF00514
**E**	Trp leader	-	48	known	RF00513
**E**	Thr leader	-	45	known	RF00506
**E**	Leu leader	-	43	known	RF00512
**F**	T-box	-	1134	known	RF00230

The RNA motifs are arranged according to ‘Classification code’ (column ‘CC’) and ‘Number of RNAs identified’ (column ‘No’)’. Effector’column includes experimentally identified or potential (marked with ‘?’) effectors of riboswitches; potential riboswitches with yet unknown effectors are marked with ‘?’; other classes of RNA motifs are indicated with ‘-‘. ‘Experimental’ column refers to *in vivo* or *in vitro* validation of a RNA motif function and/or riboswitch effector.

**Table 2:** Statistics of RNA sites, regulogs and target genes in the analyzed genomes

**Phylum / subdivision**	**Taxonomic group**	**Genomes**	**Sites**	Sites per genome*	**Regulogs**	**Target genes**	Genes per genome*
**Firmicutes**	Lactobacillaceae	15	581	39	39	1074	72
	Streptococcaceae	15	400	27	29	881	59
	Bacillales	11	668	61	39	1525	139
	Staphylococcaceae	7	288	41	30	647	92
	Clostridiaceae	20	958	48	40	2036	102
**Bacteroidae**	Bacteroidaceae	11	84	7.6	2	334	30
**Chlorobia**	Chlorobiales	11	73	6.6	6	263	24
**Actinobacteria**	Corynebacteriaceae	8	80	10	13	194	24
	Mycobacteriaceae	9	131	15	12	247	27
**Cyanobacteria**	Cyanobacteria	14	86	6.1	11	150	11
**Chloroflexi**	Chloroflexi	5	98	20	17	300	60
**Deinococcus-Thermus**	Deinococcus-Thermus	5	64	13	13	221	44
**Thermotogae**	Thermotogales	11	88	8.0	13	379	34
**Proteobacteria/Delta**	Desulfovibrionales	10	78	7.8	9	159	16
**Proteobacteria/Alpha**	Caulobacterales	4	36	9.0	8	70	18
	Rhodobacterales	15	182	12	13	443	30
	Rhizobiales	15	221	15	11	486	32
**Proteobacteria/Beta**	Burkholderia	8	127	16	9	319	40
	Ralstonia	6	66	11	10	173	29
**Proteobacteria/Gamma**	Enterobacteriales	12	188	16	18	601	50
	Pasteurellales	9	112	12	11	258	29
	Vibrionales	10	202	20	17	533	53
	Pseudomonadaceae	8	92	12	9	248	31
	Shewanellaceae	16	291	18	15	910	57
**TOTAL:**	**24 lineages**	**255**	**5204**	**20**	**394**	**12451**	**46**

*Average numbers of RNA sites and regulated genes per genome were calculated for each studied taxonomic group; the average numbers for all lineages are given in the last line.

The Rfam covariance models of each RNA motif were used to scan all selected microbial genomes using Infernal [37]. The resulting putative RNA sites were uploaded into the RegPredict web server. [36] in order to analyze their target operons, and reconstruct and functionally annotate the respective regulons by utilizing a comparative genomics approach (see Methods). The inferred gene and operon content for reconstructed RNA motif regulons in each of 255 analyzed genomes is summarized in Additional file 1. The T-box regulons, identified and reconstructed in a subset of 93 genomes from 9 taxonomic groups and classified by their amino acid specificities, are summarized in Additional file 2. The detailed results of this analysis, including sequences of all RNA sites, are captured in the RegPrecise database as the collection of regulons operated by RNA regulatory elements [38].

### Taxonomic distribution and statistics of RNA motifs, regulogs and regulated genes

By applying this workflow, we tentatively identified a reference set of 5204 RNA sites unevenly distributed in 255 genomes (Table 2). On average, this constitutes 20 RNA regulatory sites per genome. The largest average numbers of RNA sites per genome were found in all five lineages of the Firmicute phylum (from 27 to 61 sites), whereas the Cyanobacteria, Chlorobia, Bacteroidae, and Thermotogae phyla possess only between 6 and 8 sites per genome on average.

The main outcome of the comparative genomics-based analysis in RegPredict is an annotated regulog, which is defined as a set of regulons controlled by the same RNA motif family in a group of closely-related genomes. At the beginning, our analysis of 43 RNA motif families across 24 taxonomic groups had revealed 310 populated regulogs (Additional file 3). The remaining 722 potential combinations of RNA motifs and lineages have not been assigned to regulogs because of the absence of candidate RNA regulatory sites in the studied genomes. Since the identified T-box regulogs in Firmicutes have included the highest numbers of RNA sites per lineage (e.g. 291 and 337 T-boxes in Lactobacillaceae and Clostridiaceae, respectively), we split them into subsets based on their amino acid specificities. Using multiple alignment and sequence analysis of 1134 T-boxes, we determined the specifier codons and classified them into 18 amino acid specificity groups (Additional file 3). Individual analysis of amino acid-specific T-box regulons resulted in inference of 92 respective regulogs, increasing the total number of reconstructed RNA motif regulogs to 393 (Table 2).

**Figure 1:**
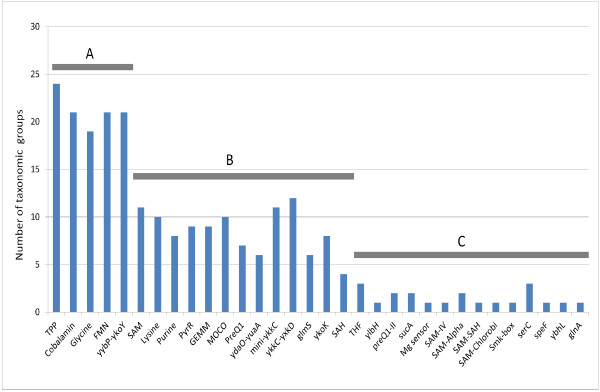
**Distribution of RNA motifs in 24 taxonomic groups of bacteria.** Each bar shows the number of microbial lineages studied that possess at least one predicted RNA motif regulon. Ribosomal and amino acid biosynthesis operon leaders and T-boxes are not shown. The classification codes: **(A)**, widely distributed RNA motifs; **(B)** moderately distributed RNA motifs; **(C)** RNA motifs with restricted taxonomic distribution.

Based on the distribution of RNA regulogs in the analyzed taxonomic groups of bacteria (Figure 1), we classified RNA motifs into three major groups (Table 1). In group A, we include the widely distributed RNA motifs (that are present in more than 75% of taxonomic groups), including riboswitches specific for cofactors (TPP, cobalamin, and FMN), glycine, and unknown substrate (yybP-ykoY). In group B, we have RNA motifs with more moderate distribution, including riboswitches specific for cofactors (preQ1, MOCO), amino acids and their derivatives (lysine, SAM, SAH), nucleotides (purine), sugars (*glmS*), metal ions (*ykoK*), secondary messengers (cyclic di-GMP), and unknown substrates (*ydaO-yuaA*, mini-*ykkC*, *ykkC-yxkD*), as well as the PyrR binding motif controlling the pyrimidine metabolism. In group C, we include RNA motifs that are restricted to no more than three taxonomic groups. The group C contains riboswitches that are specific for amino acids and derivatives (*glnA*, SAM-SAH, SAM-Chlorobi, Smk-box, SAM-Alpha, SAM-IV), cofactors (THF, preQ1-II), ions (Mg sensor), and unknown substrates (serC, speF, *ybhL*, *sucA*, *ylbH*). In addition, we classified the *cis*-regulatory RNA leaders that control the ribosomal protein operons (S15, L10, L20, L21, L19, L13) and the amino acid biosynthesis operons (Trp, His, Leu, Thr leaders) into groups D and E, respectively. T-box RNA motifs also control the amino acid metabolism, however since these *cis*-regulatory RNAs utilize a unique mechanism involving uncharged tRNA, we classified them into the separate group F (Table 1).

**Figure 2:**
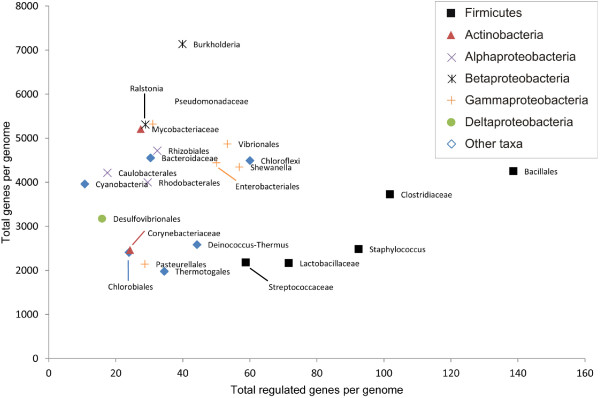
**Scatter plot of all identified RNA motif-regulated genes versus total number of genes in an individual taxon.** Each dot in the figure represents the average value of all the genomes in that taxon.

Our inference of putative regulons has included all genes co-regulated by an RNA motif and organized in putative operons. Thus the numbers of co-regulated genes are larger than the respective numbers of identified RNA sites. We calculated the total numbers of genes controlled by each reconstructed RNA motif regulog in each studied taxonomic group of bacteria (Additional file 4). The total number of RNA motif-regulated genes in all 24 lineages exceeds twelve thousands, while the average number of target genes per genome was 46 (Table 2). As expected, we observed high variation in the RNA regulon content between the studied taxonomic groups (Figure 2). Five lineages from the Firmicutes phylum had the highest average numbers of RNA motif-regulated genes (93 genes per genome on average), and these numbers were proportional to the average genome size for each lineage. In contrast, Cyanobacteria had the smallest number of regulated genes (11 genes per genome).

### Functional classification of RNA motif-regulated genes

As part of our analysis of RNA regulon content, we introduced a two-level functional classification scheme for regulated genes (Table 3). The scheme is based on functional gene annotations that were inferred in the course of regulon reconstructions in the RegPredict server (see Methods). Each gene from an RNA motif regulon was assigned to a Specific Functional Category (SFC), and also to an Overall Functional Category (OFC) according to the table in Additional file 5. Altogether, we used 45 SFC and 10 OFC categories (Table 3). In contrast to other functional classifications such as metabolic subsystems in the SEED database [39], novel functional categories permit us to make distinctions between biosynthetic and transport genes and compare the respective metabolic requirements between auxotrophic and prototrophic organisms. We restricted our analysis to those genes with either previous experimental validation, functional annotation in a SEED subsystem, or high sequence similarity to previously established functional orthologs. The remaining genes were grouped in the ‘Miscellaneous’ category.

**Table 3:** Two-level functional classification of identified RNA motif-regulated genes

**Overall functional categories (OFC)**	**Specific functional categories (SFC)**
**1. Coenzyme metabolism**	B12 biosynthesis; Thiamin biosynthesis; Riboflavin biosynthesis; Queuosine biosynthesis; Folate biosynthesis; Molybdenum cofactor biosynthesis
**2. Coenzyme uptake**	B12 & precursor transporters; Thiamin & precursor transporters; Riboflavin transporters; Queuosine & precursor transporters; Folate transporters
**3. Amino acid metabolism**	Methionine biosynthesis; Methionine & SAM recycling; Cysteine biosynthesis; Lysine biosynthesis; Glycine metabolism; Serine metabolism; Ornithine degradation; Leucine biosynthesis; Histidine biosynthesis; Threonine biosynthesis; Tryptophan biosynthesis; Amino acid biosynthesis
**4. Amino acid uptake**	Methionine transporters; Lysine transporters; Glycine transporters; Histidine transporters; Amino acid transporters
**5. Secondary metabolism**	Aminosugar biosynthesis; Citric acid cycle; Urea and agmatine utilization
**6. Metal homeostasis**	Cobalt transporters; Molybdenum & tungsten transporters; Magnesium transporters; Potassium transporters
**7. Nucleobase metabolism**	Purine metabolism; Pyrimidine metabolism
**8. Nucleobase uptake**	Purine & precursor transporters; Pyrimidine transporters
**9. Protein synthesis**	Ribosome biogenesis; Amino acyl-tRNA synthetases
**10. Miscellaneous**	Isozymes of B12-dependent enzymes; Multidrug resistance transporter; Polysaccharide degradation; Other or unknown functional roles

For each RNA motif and each taxonomic group, we calculated the numbers of genes that appeared in each SFC category (Additional file 6). The detailed analysis of RNA regulon compositions across all studied taxonomic groups using the SFC classification is described below and in the supplementary text in Additional file 7. The supporting diagrams with average numbers of RNA motif-regulated genes from each SFC in each taxonomic group are presented in Figures 3, 4, 5 and 6 (for group A riboswitches) and in supplementary figures S1-S17 within Additional File 8 (for the remaining RNA motifs). Below we describe the results of functional regulon content analysis for four widespread metabolic riboswitches from group A.

1. Cobalamin (coenzyme B_12_)

The cobalamin riboswitch regulates many enzymes responsible for the synthesis of the corrin ring from uroporphyrinogen-III, insertion of the cobalt ion into the corrin ring, and its subsequent modification to form active coenzyme B_12_. In addition, a large number of known or predicted transporters for cobalt ions (that are required for B_12_ biosynthesis) and B_12_ (that allow vitamin salvage from the environment), as well as isofunctional enzymes that provide alternatives to B_12_-dependent enzymes, have been previously found to be subject to regulation by cobalamin riboswitches in bacterial genomes [22]. We established the following four SFCs for genes included in the cobalamin regulons: (i) B_12_ biosynthesis, (ii) B_12_ and precursor transporters, (iii) cobalt transporters, and (iv) isozymes of B_12_-dependent enzymes (Additional file 5). In turn, these SFCs were classified under the broader OFC categories of coenzyme metabolism, coenzyme uptake, metal homeostasis, and miscellaneous, respectively. We identified 43 unique functional gene orthologs encoding B_12_ biosynthetic enzymes, whereas the overall numbers of gene orthologs involved in transport of vitamin B_12_ and cobalt ions were 26 and 12, respectively.

The cobalamin regulons were identified in all examined taxonomic groups except two lineages of Firmicutes (*Staphylococcus* and *Streptococcus*) and one group of γ-proteobacteria (Pasteurellales), both representing pathogenic species with reduced biosynthetic capacities (Additional file 4). Interestingly, there was one genome (*Streptococcus sanguinis*) that still retains the complete B_12_ biosynthetic pathway despite the absence of a cobalamin riboswitch. This fact suggests that the riboswitch-dependent control of cognate metabolic pathways is not ubiquitous. The cobalamin riboswitch regulons are the largest in the dataset. Altogether this dataset includes 535 RNA sites that control over 2400 genes in 197 genomes (Additional files 3 and 4). On average, these numbers correspond to 12 regulated genes per genome or 4.5 genes per riboswitch.

**Figure 3:**
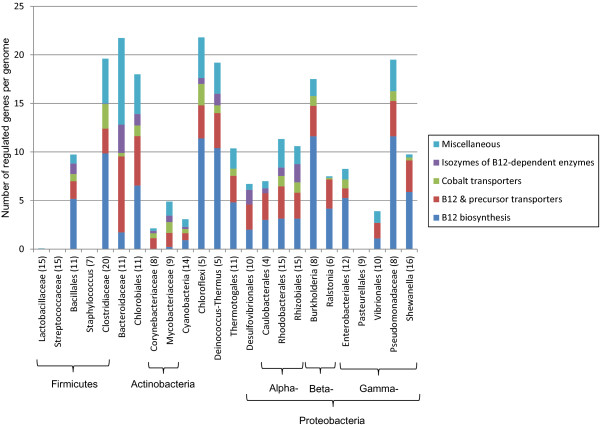
**Functional composition of cobalamin riboswitch regulons across different lineages.** Average number of riboswitch regulated genes per genome denotes the overall height of each bar. Colored bar parts show functional regulon composition using SFC categories indicated in the legend. For each taxonomic group, a number in parenthesis represents the number of studied genomes. Phylum / subdivision names are indicated by braces beneath the taxon names.

We used the SFC classification to understand functional preferences for the genes regulated by cobalamin riboswitches across all taxonomic groups (Figure 3). There is large variation in the number of these riboswitch-regulated genes, ranging from more than 20 genes per genome for Bacteroidaceae and Chloroflexi to less than ten genes for many taxa. As expected, almost all bacteria examined are capable of synthesizing and/or taking up vitamin B_12_ as indicated by their regulon content. However, the proportion of regulated genes in the B_12_ biosynthesis and transport SFCs is also highly variable among the different taxa. For example, the *Bacteroides* and *Mycobacterium* species have less than 10% of B_12_ biosynthesis genes under cobalamin riboswitch control. In contrast, 70% of cobalamin-regulated genes in *Burkholderia* are involved in B_12_ biosynthesis. In Corynebacteriaceae, no B_12_ biosynthetic genes were regulated by a cobalamin riboswitch, and the reduced cobalamin regulons mostly included B_12_ and cobalt transport genes. Noteworthy, however, the complete sets of B_12_ biosynthesis genes are still present in many corynebacterial genomes (according to the SEED database), suggesting they are either constitutive or regulated by a different mechanism.

2. TPP (thiamin pyrophosphate)

The TPP riboswitch had previously been found to control various biosynthetic enzymes and known and predicted transporters involved in the synthesis and salvage of thiamin and its metabolic precursors, hydroxymethylpyrimidine and hydroxyethylthiazole [20]. For functional description of TPP riboswitch regulons, we used the following two SFCs: (i) thiamin biosynthesis, and (ii) thiamin and precursor transporters, including 20 and 19 functional gene orthologs, respectively (Additional file 5). TPP riboswitches are the most ubiquitous RNA motifs that are present in all 24 studied taxonomic groups of bacteria (Additional file 4). These RNA motifs have been identified in all studied genomes except only six species (*Lactobacillus helveticus, Bartonella quintana, Desulfovibrio magneticus, Lawsonia intracellularis* and two *Chlorobium* spp.). It should be noted that some of the above species (e.g. *Bartonella*, *Lawsonia*) possess the thiamin biosynthesis genes but not TPP riboswitches in their genomes, again indicating that there are exceptions from almost universal regulation by coenzyme-sensing riboswitches. Altogether we annotated 564 TPP RNA sites that control near 1800 genes in 249 genomes. On average, these numbers correspond to 7 regulated genes per genome or 3.2 genes per riboswitch.

**Figure 4:**
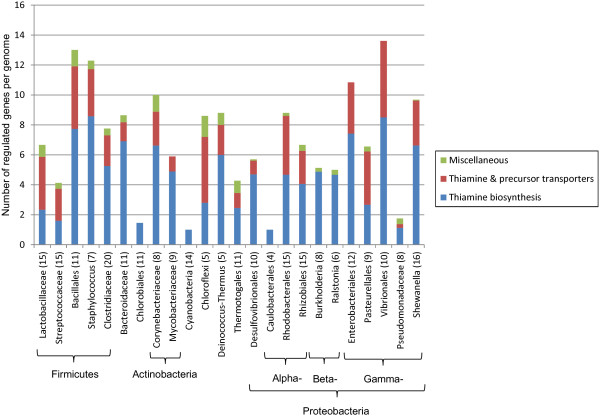
**Functional composition of TPP riboswitch regulons across different lineages.** See Figure 3 for figure descriptions.

Despite the wide distribution of TPP motifs, there is a large variation in the number of regulated genes per genome (Figure 4). Vibrionales has the highest number of regulated genes at nearly 14 genes per genome. In contrast, two other lineages from the Proteobacterial phylum, Caulobacterales and Pseudomonadaceae, have less than 2 TPP-regulated genes per genome. In all Cyanobacteria, the TPP riboswitch regulates only one gene per genome, *thiC*, which encodes an essential thiamin biosynthesis enzyme. Among TPP-regulated genes, thiamin biosynthesis genes dominate the SFC categories in the majority of lineages, accounting for over 65% in most cases. The thiamin and thiamin precursor transporter genes constitute the next most abundant SFC and can be found in almost all the studied lineages except *Burkholderia*, Caulobacterales, Chlorobiales, Cyanobacteria, and *Ralstonia*. On average, the transporter genes represent 30% of TPP-regulated genes. However, the thiamin transporters dominate over the biosynthesis genes in Chloroflexi, Lactobacillaceae, Pasteurellales, and Streptococcaceae, accounting for over the half of the total regulated genes. The abundance of various TPP-regulated transporters in this species correlates with their predicted thiamin auxotrophic phenotypes, caused by the absence of thiamin biosynthetic genes.

3. FMN (Flavin mononucleotide)

Similarly to TPP regulons, the FMN riboswitch regulons were classified into two SFCs: (i) riboflavin biosynthesis, and (ii) riboflavin transporters, that include seven and five functional gene orthologs, respectively (Additional file 5). The FMN regulons were identified in all examined taxonomic groups except Bacteroidaceae, Mycobacteriaceae and Cyanobacteria (Additional file 4). Bacteria from these three lineages possess the riboflavin biosynthesis genes in their genomes; however these genes are not associated with FMN motifs. In general, there are fewer FMN motifs and genes in their regulons when compared to the TPP and cobalamin riboswitches. Altogether we annotated 233 FMN RNA sites that control near 432 genes in 183 genomes. On average, these numbers correspond to 2.4 regulated genes per genome or 1.9 genes per riboswitch.

**Figure 5:**
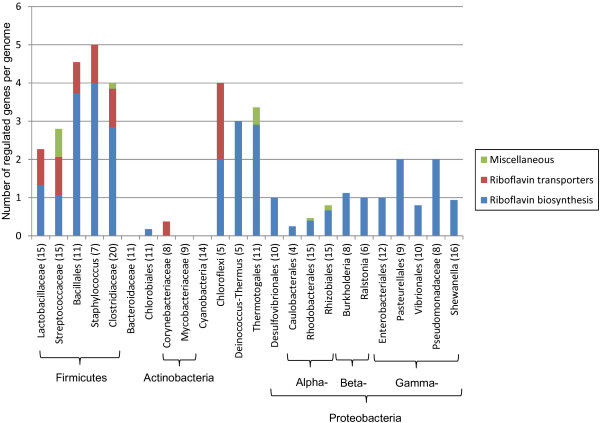
**Functional composition of FMN riboswitch regulons across different lineages.** See Figure 3 for figure descriptions

Based on the analysis of distribution of FMN riboswitch-regulated genes (Figure 5), roughly two distinct patterns of regulation were identified. In most lineages of Firmicutes, as well as in Chloroflexi, the *Deinococcus-Thermus* group and Thermotogales, there are three to five FMN-regulated genes per genome, and the riboflavin biosynthesis genes (often organized into the *ribDEBA* operon) represent the largest proportion of regulated genes. In contrast, most species from the Proteobacterial phylum, as well as two *Chlorobia* spp, have a single FMN-controlled gene involved in riboflavin biosynthesis (*ribB* or *ribH2*). In contrast to cobalamin and TPP, transporters specific for riboflavin are not as widely distributed and can only be found in 7 taxonomic groups (representing just over 15% of all regulated genes on average). Interestingly, the riboflavin transporter *pnuX* is the only FMN-regulated gene in Corynebacteria.

4. Glycine

**Figure 6:**
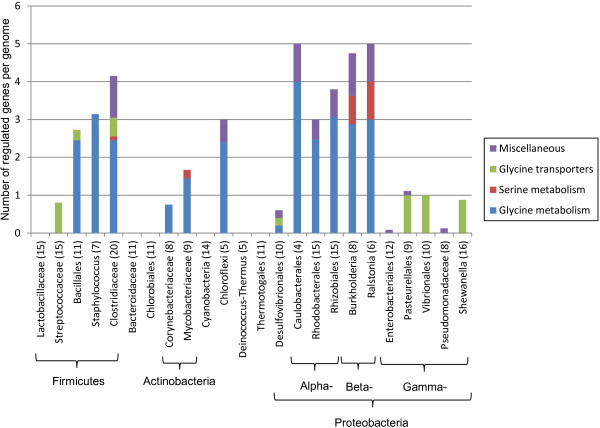
**Functional composition of glycine riboswitch regulons across different lineages.** See Figure 3 for figure descriptions.

The glycine riboswitch-regulated genes were classified into three SFCs: (i) glycine metabolism, (ii) glycine transporters, and (iii) serine metabolism that include twelve, three and three functional gene orthologs, respectively (Additional file 5). Glycine riboswitches were found in 18 taxonomic groups including all studied lineages of Proteobacteria, four groups of Firmicutes, both Actinobacterial groups and Chloroflexi (Figure 6). Altogether we annotated 324 glycine RNA sites that control near 415 genes in 145 genomes. On average, these numbers correspond to 2.2 regulated genes per genome. However, in contrast to other RNA motifs, most glycine-regulated operons (82%) are preceded by two copies of a glycine riboswitch. Such a tandem arrangement of glycine regulatory RNAs may promote positive cooperative responses to increasing concentrations of glycine [40]. Taking into account these tandem riboswitches, the average size of glycine-controlled operons equals to 2.3 genes.

Three groups of Firmicutes, α- and β-proteobacteria and Chloroflexi have shown the largest numbers of glycine-regulated genes (Figure 6). As expected, most of these genes (67% on average) are involved in glycine metabolism. In contrast, glycine transporter genes occurred with much lower frequencies in different taxonomic groups at 15% on average. However, the overall regulon content is not well conserved between the different lineages. For instance, glycine transporter genes make up almost the entirety of the glycine regulons in Streptococci and three branches of γ-proteobacteria but are rarely encountered at all remaining taxa. Despite the ease of conversion between glycine and serine, genes responsible for serine metabolism are rarely regulated by glycine riboswitches. Of all the regulated serine metabolic genes, *sdaA*, responsible for interconversion between pyruvate and serine, was found in β-proteobacteria and two *Mycobacterium* spp., while *serC* and *serA* bridge the pathways between glycolysis and serine biosynthesis and were found in a single *Clostridium* species (Additional file 1).

### Distributions of overall functional categories across taxonomic groups and RNA motif families

**Figure 7:**
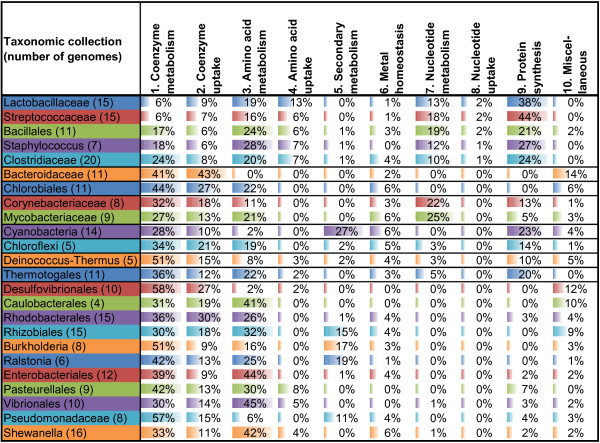
**Proportion of overall functional categories in all reconstructed RNA regulons for individual taxonomic groups.** For each taxonomic group, the relative contribution of ten overall functional categories (OFCs) to the RNA-regulated genes with assigned functions is shown. The proportions are calculated based on cumulative OFC statistics for all studied RNA families provided in Additional file 6 and exclude functionally unknown genes. The colored bar in each cell is proportional to the total number of OFC in each taxon, with different colors used for each row. Under the first column, the number following the name of each taxon represents the number of genomes examined in this study. The taxonomic groups are arranged according to their phylogeny.

**Figure 8:**
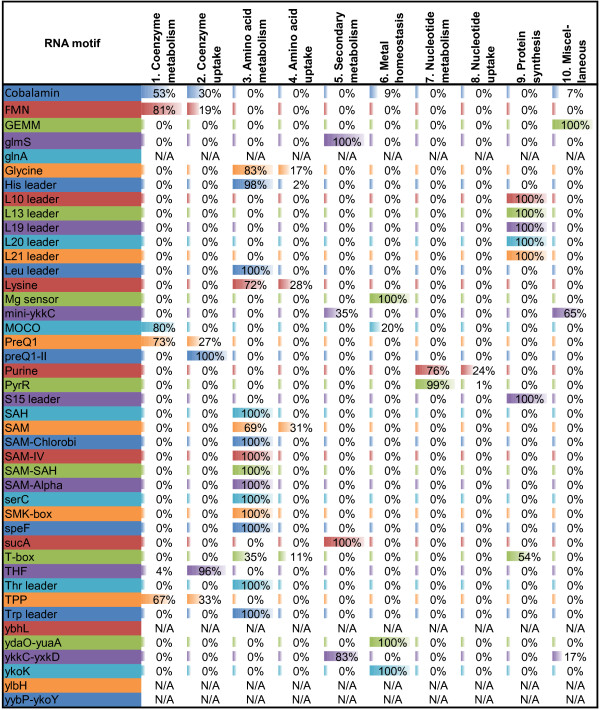
**Proportion of overall functional categories for individual RNA motifs.** For each RNA motif, the relative contribution of ten overall functional categories (OFCs) to the riboswitch-regulated genes with assigned functions is shown. The proportions are calculated based on cumulative OFC statistics for all studied taxonomic groups provided in Additional file 6 and exclude functionally unknown genes. The colored bar in each cell is proportional to the total number of OFC for each RNA motif family with different colors used for each row. RNA motifs controlling genes without assigned OFCs have their rows labeled as ‘N/A’.

Ten overall functional categories (OFCs) were introduced in this work to facilitate the functional content analysis of reconstructed RNA regulons (Table 3). OFCs provide a more general biological classification for 45 specific functional categories (SFCs) assigned to RNA motif-regulated genes. We calculated the relative contribution of these OFCs to the cumulative pools of all regulated genes with assigned SFCs independently for each taxonomic group (Figure 7) and for each RNA motif family (Figure 8).

When all RNA motif regulons were combined, the coenzyme metabolic category appeared to be the most abundant OFC, ranging from ~20% to over 50% across different taxonomic lineages (Figure 7). Exceptions were noted in some Firmicutes, especially Lactobacillaceae and Streptococcaceae, which only have coenzyme metabolic genes accounting for 6% of regulated contents. The next most abundant OFC is the amino acid metabolic category, with high proportions in many lineages of Firmicutes, γ-, and α-proteobacteria. On the other end of this distribution spectrum, the genes responsible for nucleotide metabolism were generally low across different lineages, with notable exceptions in Firmicutes and Actinobacteria. This taxon-specific abundance is also exhibited in other OFCs. For example, Firmicutes have high proportions of regulated protein synthesis genes, ranging from 20% to over 40%. In Cyanobacteria, the abundance of genes for secondary metabolism and protein synthesis was also noted with each accounting for roughly a quarter of regulated genes.

When analyzed according to RNA motif family, most regulons showed predictable OFC content (Figure 8). For instance, most of regulons controlled by the cofactor and amino acid specific riboswitches contain OFCs for the respective metabolic pathways and transporters, while the ribosomal and amino acid leaders and T-boxes regulons contain OFCs for protein synthesis and amino acid metabolism, respectively. Among miscellaneous OFCs, the GEMM riboswitch contains genes responsible for polysaccharide degradation (21 genes counted) although the majority of the regulon content exhibits unknown functions (114 genes). Even though both the *ykkC-yxkD* and mini*-ykkC* RNA motifs are fairly well-distributed with many taxonomic overlaps (Additional file 1), they regulate a similar set of genes with OFC, specific for multidrug resistance transporter as well as urea and agmatine utilization; however, the proportion of multidrug resistance transporter is higher in the mini-*ykkC* regulons (65%) when compared to the *ykkC-yxkD* regulons (17%). A similar situation was observed between the preQ1 and preQ1-II riboswitches for queuosine metabolism and uptake. preQ1 regulates a small proportion of genes related to coenzyme uptake (27%), whereas the remaining target genes are classified as the coenzyme metabolic OFC. In contrast, preQ1-II exclusively regulates genes from the coenzyme uptake OFC.

## Discussion

While much effort has been put into discovering novel RNA *cis*-regulatory sequences in bacterial genomes, there have been few attempts at analyzing their overall distributions or even the operon contents of individual RNA-regulated operons across different microbial genomes. In this study we established regulatory capacities of different *cis*-regulatory RNAs and used comparative genomics to describe potential functions for many previously uncharacterized genes in RNA regulons.

Analysis of RNA motif abundances and diversity allowed us to make certain predictions regarding the origins of some of these regulators. Among the most widespread group of RNA motifs (Group A), many riboswitches are specific for cofactors such as TPP, cobalamin, and FMN (Figure 1 and Additional file 2). As these cofactors are essential for cellular functions such as carbon metabolism, DNA repair, and energy production, their abundances give credence to the notion of ancient origins of riboswitches. Still, there are other cofactor-related riboswitches from groups B and C that are much more rare in their taxonomic distributions. These include riboswitches controlling the metabolism of tetrahydrofolate (THF) and queuosine (preQ1, preQ1-II) that are mostly limited to Firmicutes, whereas the putative molybdenum cofactor (MOCO) riboswitch is ubiquitous in γ-proteobacteria but also can be sporadically found in several other bacterial lineages (Additional file 7). Second, there are several widespread riboswitches from groups A and B that are specific for amino acids including glycine, lysine and the methionine derivative, S-adenosylmethionine (SAM). The essentiality of amino acids and the SAM cofactor supports the relatively ancient introduction of these riboswitches that could be proposed based their wide distribution across bacterial phyla. On the other hand, the existence of multiple distinct families of riboswitches that recognize the same ligand demonstrates great flexibility inherent in an RNA molecule. Most notably, there are seven riboswitch families with four distinct binding pockets that are specific for SAM and/or S-adenosylhomocysteine (SAH) although it is not clear whether they share common ancestries [2]. The occurrence of narrow phylogenetic distributions of many SAM-binding riboswitches from group C (SAM-IV, SAM-Alpha, SAM-SAH, SAM-Chlorobi, Smk-box) argues for their relatively recent emergence in individual taxonomic lineages.

The occurrences of many riboswitches from group B in taxonomically distant organisms suggest two possible scenarios for their evolutionary histories (Additional file 3). First, a large extent of horizontal transfer probably occurred in the evolution of several riboswitch families. For example, the preQ1and purine riboswitches that are ubiquitous in Firmicutes were also found in one or two lineages of Proteobacteria, whereas the SAH riboswitches that are most abundant in β/γ-proteobacteria were also identified in Mycobacteriaceae. Second, the elimination of RNA motifs in certain lineages at a later time could have contributed to a moderate distribution of several RNA families including SAM, GEMM, MOCO, and *ykkC-yxkD*. For instance, the SAM riboswitch (also known as the S-box) controlling methionine metabolism is widely distributed in three lineages of Firmicutes and is also present in diverse species from eight other phyla, suggesting an ancient origin. Within the Firmicutes phylum, the loss of SAM riboswitches in Streptococcaceae and almost all *Lactobacillus* genomes is correlated with the emergence of several novel regulatory systems for the control of cysteine and methionine metabolism including two transcription factors (CymR and MtaR), the Smk-box RNA motif, and the expansion of methionine-specific T-boxes [23,31]. In many cases, a mosaic phylogenetic distribution of RNA motifs in microbial genomes is a likely to have resulted from a combination of both evolutionary scenarios. For example, the lysine riboswitch is widespread among Firmicutes and also occurs in most γ-proteobacterial lineages and in most Thermotogales species. The phylogenetic analysis supports the hypothesis that the lysine biosynthetic operon and its cognate lysine riboswitch have been acquired by a common ancestor of Thermotogales via horizontal transfer from a *Clostridium*-like Firmicute [21].

With the diversity and numbers of riboswitches and other *cis*-regulatory RNA motifs analyzed, we proceeded to look at the operon contents of these regulatory elements in more detail. Within group A riboswitches, we found great diversity in operon contents that reflect different ways the same metabolites are synthesized or utilized across different organisms. A majority of genes encoded in group A operons are biosynthetic or metabolic genes that are dedicated to core metabolism such as carbon metabolism and energy production; a few were dedicated to transport of metabolites or their precursors (Figures 3, 4, 5 and 6). Still, a large portion of genes regulated by *yybP-ykoY* riboswitches appeared to encode transporters tasked with tellurium resistance as well as transport of unknown substrates. The fact that *yybP-ykoY* riboswitches are widespread suggests basic cellular roles such as osmoregulation, and/or resistance to toxic metabolites.

The cobalamin (B_12_) riboswitch regulates a variety of genes in several phyla, and the difference in operon contents may be influenced by lifestyle and/or habitat of individual species. For instance, Bacteroidaceae species are part of the human intestinal microflora, and the ability to synthesis B_12_ may be replaced by B_12_ transporters or transporters with multiple specificities (Figure 3). In Chloroflexi, most of the analyzed free-living species have the cobalamin-regulated B_12_ biosynthetic genes; however the lack of the biosynthetic genes in a single species from this phylum, *Herpetosiphon aurantiacus*, correlates with its capability of predation on other bacteria, and the presence of the riboswitch-regulated B_12_ transporter suggests its probable dependence on extracellular vitamin B_12_. Even in organisms with the complete biosynthetic pathway, the existence of B_12_ and precursor transporters may impart selective advantages during times of hardship or during different life stages.

From our observations of TPP riboswitch regulons, many taxa examined seem to be capable of producing thiamin and its biologically active form, TPP. Even for those lineages with very few regulated genes, such as Caulobacterales, Cyanobacteria, and Chlorobiales, genes required for the synthesis of both hydroxyethylthiazole and hydroxymethylpyrimidine precursors of thiamin appeared to exist. However, even though thiamin precursors can be readily obtained via amino acid and purine metabolism, thiamin and precursor transporter genes are surprisingly abundant even among those organisms that already contain the enzymes necessary for its biosynthesis (Figure 4). Presumably, the ability for thiamin and precursor transport grants selective advantage when other pathways compete with the thiamin biosynthetic pathway for available amino acids and purines. In contrast, many other species and bacterial phyla lack thiamin precursor biosynthesis, and this is compensated for by the presence of a variety of transporters for salvage of thiamin precursors from the environment. For example, thiamin auxotrophic species from the Chloroflexi phylum possess several TPP-regulated transport systems for uptake of both thiamin precursors and thiamin itself.

Regarding FMN riboswitches, most riboflavin biosynthetic genes (often organized in a single operon) and the riboflavin transporter genes, *ribU*, are controlled by FMN riboswitches in Firmicutes (Figure 5). On the other hand, Bacteroidaceae, Cyanobacteria and Mycobacteriaceae do not contain FMN riboswitch, though the riboflavin biosynthesis pathway is conserved. Despite having only riboflavin transporter genes being regulated by FMN riboswitches, the riboflavin biosynthesis pathway appears to be retained in Corynebacteriaceae. However, whether it is regulated or not is unknown. Interestingly, our analysis of FMN regulons identified for the first time a novel type of riboflavin transporter in Chloroflexi, termed RibXY, which is homologous to the ABC-type thiamin transporter ThiXYZ but lack an ATPase component. In all analyzed Chloroflexi genomes, the *ribXY* transporter operons are preceded by FMN riboswitches, and in one case, in *Roseiflexus castenholzii,* it compensates for the absence of a riboflavin biosynthetic operon.

Even though only glycine transporter genes were found to be regulated by glycine riboswitches in Streptococcaceae and certain γ-proteobacteria, glycine biosynthetic genes exist elsewhere in the genomes of these organisms for *de novo* production of glycine. The regulatory differences observed between glycine biosynthetic and transporter genes may reflect the different metabolic needs of these organisms. Although the regulatory mechanisms for glycine and serine metabolic genes were expected to be similar, given the ease of conversion between these two amino acids, serine metabolic genes were largely absent in most of glycine riboswitch regulons. Even in those lineages in which serine metabolic genes were found, they constitute a small proportion of overall regulated genes. Our findings may reflect the ways these two amino acids are utilized inside a cell. For example, serine may be more valuable as a precursor for energy production in glycolysis than as a precursor for other amino acids, but under most circumstances, glycine may better serve as a building block.

In contrast to group A, group B RNA motifs are responsible for regulating a wide variety of metabolic processes including the transport of amino acids, cofactors, nucleotides and metal ions. Still, many regulate genes with unknown cellular functions. The fact that frequent horizontal transfer probably occurred in this group of RNA motifs suggests peripheral metabolic functions that, when incorporated, confer certain survival benefits. Many novel genes were also found associated with other group B motifs, and their functions can be putatively assigned based on their regulators. For example, various genes found in Bacillales, Clostridiaceae, and Chloroflexi under SAM riboswitch regulation are probably involved in cysteine and methionine metabolism or the transport of corresponding substrates. Similarly, many genes regulated by GEMM RNA motifs can be assigned to cellular motility, virulence and biofilm formation. Some preQ1 riboswitch regulated genes of unknown function may function in the queuosine biosynthesis or salvage pathways; examples of this include *iunH* and *folE* from genomes of many Firmicutes. The *ydaO-yuaA* riboswitch regulates a wide variety of transporters, which may all play roles in osmoregulation; in addition, regulated cell wall remodeling enzymes like COG0791 and COG3773 from Corynebacteriaceae and Clostridiaceae may serve similar roles. *ykkC-yxkD* and the functionally equivalent mini-*ykkC* riboswitches both have been proposed previously to function in regulating efflux pumps and other detoxification mechanisms; however, our findings also yielded genes (COG3382 and COG731 from Clostridiaceae) that function in capacities other than transport. For SAH riboswitches, some novel genes (PF04020 and PF04993) were predicted in *Ralstonia* although their functional contribution to SAM/SAH homeostasis is unknown.

Group C RNA motifs are phylogenetically confined to a few closely related taxonomic groups. Their limited distribution points to their recent invention and, as a consequence, their existence in these few taxa may have been the result of vertical transmission. Their regulon contents are also highly conserved with little variation between genomes, thus providing additional evidence for vertical transmission.

For group D RNA motifs, even though ribosomal subunits are highly conserved between different bacterial lineages, their regulatory regions appear to exhibit greater variation than the coding sequences. Four studied motifs (L13, L19, L20, L21) that regulate the large ribosomal subunits appear to be confined to all lineages of Firmicutes but were also identified in Thermotogales. It is plausible that these four RNA motifs were originated in Firmicutes, and that the L20 and L21 motifs in Thermotogales were horizontally acquired from Firmicutes. The L10 and S15 motifs have wider taxonomic distributions, being identified in 13 and 10 lineages, respectively (Additional file 3). As ribosomal subunits are tightly regulated, it may be worthwhile to reexamine their regulatory regions in those lineages with apparently missing known motifs to detect weakly conserved regulatory RNAs or even novel RNA regulatory elements. Two recent genomic studies of ribosomal regulatory RNAs from *E. coli* and *B. subtilis* reported that most of these RNA structures were narrowly distributed in γ-proteobacteria and Bacilli, respectively, and that many distinct ribosomal RNA motifs may yet to be identified in bacteria [18,19].

Group E regulatory elements are amino acid attenuators (excluding T-boxes that utilize uncharged tRNA as the signal molecule), and their substrate specificities are currently restricted to histidine, leucine, threonine and tryptophan, as only these four RNA motifs were available in the Rfam database. In addition to these four motifs, a couple of other amino acid attenuator motifs specific to isoleucine/valine and phenylalanine were previously characterized [17]; however they are not currently represented in Rfam. The amino acid attenuator motifs may have originated from γ-proteobacteria with very few cases of horizontal transfer. Their target operons were highly conserved as well, with most genes encoding enzymes of amino acid biosynthesis. However, despite the lack of group E regulators in many genomes, the presence of the corresponding amino acid metabolic pathways is widespread, and the use of regulators other than amino acid attenuators may explain this difference. For instance the histidine and tryptophan biosynthesis operons in Bacillales are regulated by the transcription factor HisR [41] and an RNA-based mechanism involving the tryptophan-activated RNA-binding attenuation protein TRAP [42], respectively.

For group F, despite the ubiquity of tRNAs and their associated genes in bacteria, the distribution of T-box RNA motifs is rather limited to Firmicutes, Actinobacteria, Chloroflexi and the Thermus/Deinococcus group. In addition, T-boxes have been previously found in *Geobacter* spp. [13], which belongs to the δ-proteobacterial lineage that was not analyzed here. Two potential scenarios on evolutionary origin of T-box RNA motifs have been proposed: (i) the development of T-boxes in the Firmicutes phylum with subsequent horizontal transfers to other taxonomic groups, and (ii) the presence of T-boxes in a common ancestor of bacteria from the above diverse phyla [13-15]. Aminoacyl-tRNA synthetase genes are the most frequently regulated genes followed by amino acid biosynthesis and then amino acid transporter genes. Among the evolutionary processes shaping the modern T-box regulons in Firmicutes, there were numerous duplications of T-boxes and changes in their amino acid specificity [14]. Many cases of expansion of amino acid-specific T-box regulons were noted in different lineages and genomes of Firmicutes such as the methionine T-box regulon in Lactobacillales [23].

## Conclusions

In this study, we performed a comprehensive comparative genomics analysis of RNA regulons controlled by 43 Rfam motifs including metabolite-sensing riboswitches, ribosomal and amino acid operon leaders, and T-boxes. As result, 5204 RNA regulatory sites were identified in 255 complete genomes demonstrating a high variability in the genomic distributions of numbers and types of RNA sites across diverse taxonomic groups of bacteria. We further performed reconstruction and functional annotation of 3251 RNA regulons controlling over 12,000 genes. Each regulon was defined as a set of genes in a single genome controlled by RNA regulatory sites from the same Rfam family. To facilitate the comparative analysis of this large number of regulons, we used the regulog approach implemented in the RegPredict web server. All studied organisms were subdivided into 24 taxonomic groups, and the analyses of RNA regulons were performed independently in each lineage, resulting in reconstruction of 393 regulogs. Based on taxonomic distribution of the obtained regulogs, each known or putative riboswitch was assigned to one of three groups, whereas the ribosomal and amino acid leaders and T-boxes were classified independently. Further, we introduced a two-level functional classification of genes controlled by each analyzed RNA motif. Specific functional categories (SFCs) allowed us to analyze functional preferences for each individual RNA motif family across taxonomic groups. Overall functional categories (OFCs) provided an overview of biological processes controlled by RNA motifs by taxonomy and also according to RNA motif family.

Our analyses allowed inferences of possible evolutionary pathways for many of the examined RNA regulatory elements as well as functional annotations to the regulated genes. Our results suggested that some RNA motifs may have arisen in the last common ancestor of most or all bacteria, but far more may have arisen relatively recently. Additional analyses of the metabolic pathways from organisms missing the corresponding RNA motifs suggested that functionally equivalent regulators are also widespread. Despite the simplicity of *cis*-regulatory RNA regulation and the ease of horizontal transmission for these regulators, the preservation of other regulators appears necessary for normal metabolism in other organisms. Our functional annotations of many of regulated genes will allow researchers to validate our initial predictions.

The results of this study suggest that many metabolic RNA motif regulons evolve rapidly in microbial lineages to adapt to changes in habitats and lifestyles. The reconstructed RNA regulons obtained for the reference collection of genomes will provide the basis for computational propagation of these regulons to new genomes. An expanded set of genomes with mapped RNA motif regulons will enable more detailed studies on the co-evolution of these regulatory RNAs and their cognate metabolic pathways.

## Methods

255 complete genomes of bacteria analyzed in this study (Additional file 1) were downloaded from the MicrobesOnline database [43]. Computational searches of *cis*-regulatory RNAs in the uploaded genomes were performed using covariance models of 43 RNA motifs uploaded from the Rfam database [35]. Each covariance model is a probabilistic model, which describes the secondary structure and primary sequence of an RNA motif [44]. Scanning of genomes using the covariance models was performed by the Infernal tool [37], using the noise threshold provided for each RNA motif model in Rfam. Computations were performed on a Linux-based computational cluster in the Sanford-Burnham Medical Research Institute (La Jolla, CA). The amino acid specificities of T-boxes were determined by using their multiple sequence alignments and subsequent visual determination of the specifier codons as previously described [23]. The identified candidate RNA regulatory elements (sites) were uploaded into the RegPredict Web server (http://regpredict.lbl.gov) [36] to perform comparative genomics-based analyses and reconstruction of the respective RNA regulons.

The RegPredict tool allows simultaneous analysis of multiple microbial genomes and integrates information on location of regulatory sites, gene orthologs, operon predictions, and functional gene annotations. Each RNA motif regulon was independently analyzed in each of the 24 selected groups of closely-related genomes. The genome scanning procedure implemented in RegPredict utilizes the pre-computed files with genomic coordinates and scores of candidate RNA sites from each Rfam family. The genome scan parameters were set up to reduce the chance of nonfunctioning sequences from being detected. Specifically, positions of RNA sites were set between 500 nucleotides upstream and 100 nucleotides downstream of a gene start codon. The maximum intergenic distance for an operon boundary was set to 200 nucleotides under ‘Operon definition’, and the ‘coding region overlap’ option was chosen. After genome scanning and filtering, the RNA motif regulons were clustered into groups termed Clusters of co-Regulated Orthologous operoNs (CRONs) in RegPredict [36]. This approach helped to significantly reduce the overprediction rates. For each analyzed regulon, the set of constructed CRONs was prioritized based on the level of conservation of regulatory interactions, allowing focusing on the most prominent regulon members. At the next step, the functional and genomic context analysis of each CRON was conducted using the advanced web interface, facilitating the decision on CRON inclusion and its gene content in the final regulon model. Although initial operon boundaries were set to 200 nucleotides, additional orthologues can be inferred through comparative genome analysis to expand the operon boundary. Similarly, the location of an initial regulatory region can also be expanded beyond the initial range to accommodate the discovery of new CRONs. Combining all accepted CRONs for a given RNA motif yields the reconstructed RNA motif regulon for a group of target genomes.

Biological functions of regulated genes were predicted and annotated in RegPredict by a combination of a sequence similarity search against the Swiss-Prot section of the UniProtKB database [45], domain architecture analysis in MicrobesOnline [43], and using functional gene annotations from the SEED [39] and KEGG [46] databases. Complete descriptions of the reconstructed regulons were uploaded to the RegPrecise database (http://regprecise.lbl.gov) [38], where they are publicly available within the RNA family collections of regulons. The regulatory annotations obtained for RNA regulons can be also programmatically accessed via the RegPrecise web services [47].

## Abbreviations

SAM: S-adenosylmethionine; SAH: S-adenosylhomocysteine; tRNA: Transfer RNA; ECF: Energy-coupling factor; SFC: Specific functional category; OFC: Overall functional category; TPP: Thiamin pyrophosphate; FMN: Flavin mononucleotide; MOCO: Molybdenum cofactor; THF: Tetrahydrofolate; CRON: Cluster of co-regulated orthologous operons.

## Competing interests

The authors declare that they have no competing interests.

## Authors’ contributions

DAR and PSN conceived and designed the research project. EIS and DAR wrote the manuscript. EIS and SAL performed comparative genomic analysis for reconstruction of regulons. DAR also provided the quality control as a curator. MDK computed RNA regulatory sites in microbial genomes. MHS contributed to the development of the project and writing of the manuscript. All authors read and approved the final manuscript.

## Supplementary Material

Additional file 1 **The inferred content of 42 RNA motif regulons in microbial genomes from 24 taxonomic groups.** Each cell contains all genes and operons predicted to be regulated by a particular RNA motif in a genome. Multiple candidate operons are separated by semicolons. In the operon description, dashes separate neighboring genes but are omitted for genes sharing the same locus mnemonic. Parentheses indicate fused genes. Click here for file

Additional file 2 **The inferred content of 19 amino acid-specific T-box regulons in microbial genomes from 9 taxonomic groups.** Each cell contains all genes and operons predicted to be regulated by a particular RNA motif in a genome. Multiple candidate operons are separated by semicolons. In the operon description, dashes separate neighboring genes but are omitted for genes sharing the same locus mnemonic. Parentheses indicate fused genes. Click here for file

Additional file 3 **Distribution of RNA motifs in microbial genomes.** Riboswitches are arranged in six groups according to abundance and substrate types. Click here for file

Additional file 4 **Total number of genes controlled by predicted RNA motif regulons in 24 taxonomic groups of Bacteria.** Riboswitches are arranged in six groups according to abundance and substrate types. Click here for file

Additional file 5 **Classification of identified RNA motif-regulated genes by specific and overall functional categories (SFC and OFC).** Only genes with annotated functional roles in the RegPrecise database are shown under ‘gene name’; number of gene families indicates the total types of different orthologues found. Click here for file

Additional file 6 **Distribution of Specific Functional Categories regulated by each RNA motif family across 24 analyzed taxonomic groups.** SFC number for individual riboswitch family is listed in each row, with averaged proportion listed in the last column; ‘per genome’ values and listed at the bottom. Click here for file

Additional file 7 **Comparative genomics analysis of functional regulon content for RNA motifs from groups B and C, ribosomal and amino acid operon leaders (groups D and E), and T-boxes (group F).** Additional text describes detailed analyses of the remaining RNA motif families that were not included in the main manuscript text. Click here for file

Additional file 8 **Supporting figures S1-17 for Additional file **7 (Comparative genomics analysis of functional regulon content for RNA motifs from groups B, C, D, E, F). Each figure represents functional composition of RNA motif regulons across different lineages. Average number of RNA-regulated genes per genome denotes the overall height of each bar. Colored bar parts show functional regulon composition using SFC categories indicated in the legend. For each taxonomic group, a number in parenthesis represents the number of studied genomes. Phylum / subdivision names are indicated by braces beneath the taxon names. Click here for file
