# Supplementary material for: Comparative genomics of metabolic capacities of regulons controlled by cis-regulatory RNA motifs in bacteria
Source: BMC Genomics. 2013 Sep 2;14:597. doi: 10.1186/1471-2164-14-597 (PMC3766115; doi:10.1186/1471-2164-14-597)
Supplement: Additional file 3 — Distribution of RNA motifs in microbial genomes. Riboswitches are arranged in six groups according to abundance and substrate types. [file 1471-2164-14-597-S3.pdf]

Additonal File 3. Distribution of RNA motifs in microbial genomes.

Taxonomic collection	Geno-mes	Group A - Widely distributed RNA motifs					Group B Moderately distributed RNA motifs												
		TPP	Cobalamin	Glycine	FMN	yybP-ykoY	SAM	Lysine	Purine	PyrR	GEMM	MOCO	PreQ1	ydaO-yuaA	mini-ykkC	ykkC-yxkD	glmS	ykoK	SAH
Lactobacillaceae	15	36	1	0	19	13	2	25	18	49	0	0	9	0	0	2	1	10	0
Streptococcaceae	15	23	0	18	19	15	0	4	11	54	0	0	2	0	0	0	0	5	0
Bacillales	11	47	18	22	20	16	98	23	49	31	9	0	15	18	0	11	11	7	0
Staphylococcaceae	7	30	0	8	14	7	40	15	7	13	0	0	13	0	0	1	7	0	0
Clostridiaceae	20	62	50	52	38	35	72	38	41	45	29	6	23	18	0	11	17	10	0
Bacteroidaceae	11	19	65	0	0	0	0	0	0	0	0	0	0	0	0	0	0	0	0
Chlorobiales	11	11	34	0	2	0	14	0	0	0	0	1	0	0	0	0	0	0	0
Corynebacteriaceae	8	23	6	4	3	10	1	0	0	6	0	0	0	5	0	0	0	1	0
Mycobacteriaceae	9	22	21	19	0	13	0	0	0	8	0	0	0	9	0	3	0	9	6
Cyanobacteria	14	14	20	0	0	4	2	0	0	0	6	0	0	8	1	6	0	0	0
Chloroflexi	5	14	13	6	8	5	14	0	0	3	0	0	0	0	0	0	5	0	0
Deinococcus-Therm	5	10	12	0	5	5	9	0	0	2	3	1	0	0	0	0	3	0	0
Thermotogales	11	16	20	0	8	1	1	9	2	0	0	2	0	0	0	0	0	0	0
Desulfovibrionales	10	21	23	6	10	7	4	0	0	0	5	0	0	1	1	0	0	0	0
Caulobacterales	4	4	7	11	1	4	0	0	0	0	0	1	0	0	4	0	0	0	0
Rhodobacterales	15	30	48	31	7	1	0	0	0	0	0	1	1	0	10	3	0	0	0
Rhizobiales	15	30	57	36	12	3	0	0	0	0	0	0	0	0	12	7	0	0	0
Burkholderia	8	18	38	20	9	12	0	0	0	0	0	0	0	0	9	5	0	0	8
Ralstonia	6	6	7	22	6	8	0	0	0	0	1	0	0	0	2	2	0	0	6
Enterobacteriales	12	38	16	2	12	21	0	5	0	0	1	12	0	0	10	1	0	5	0
Pasteurellales	9	20	0	18	9	0	0	10	0	0	0	12	9	0	0	0	0	0	0
Vibrionales	10	30	13	20	8	22	0	22	6	0	15	10	0	0	3	0	0	1	0
Pseudomonadaceae	8	9	33	2	8	11	0	0	0	0	0	0	0	0	8	6	0	0	7
Shewanella	16	31	34	27	15	19	0	35	7	0	20	16	0	0	7	0	0	0	0
TOTAL	255	564	536	324	233	232	257	186	141	211	89	62	72	59	67	58	44	48	27
number of regulogs	24	24	21	18	21	21	11	10	8	9	9	10	7	6	11	12	6	8	4

Taxonomic collection	Group C Phylogenetically restrictive RNA motifs														Group D Ribosomal operon leaders						Group E - a.a. operon leaders				Group F T-boxes
	THF	ylbH	preQ1-II	sucA	Mg sensor	SAM-IV	SAM alpha	SAM-SAH	SAM Chlorobi	Smk box	serC	speF	ybhL	glnA	S15	L10	L20	L21	L19	L13	Trp	His	Leu	Thr	*T-boxes
Lactobacillaceae	8	0	3	0	0	0	0	0	0	13	0	0	0	0	9	15	15	15	14	13	0	0	0	0	291
Streptococcaceae	4	0	14	0	0	0	0	0	0	0	0	0	0	0	15	15	15	15	13	15	0	0	0	0	143
Bacillales	0	10	0	0	0	0	0	0	0	0	0	0	0	0	0	11	9	11	10	8	0	0	0	0	214
Staphylococcaceae	0	0	0	0	0	0	0	0	0	0	0	0	0	0	0	7	7	6	7	7	0	0	0	0	99
Clostridiaceae	11	0	0	0	0	0	0	0	0	0	0	0	0	0	3	19	9	15	17	0	0	0	0	0	337
Bacteroidaceae	0	0	0	0	0	0	0	0	0	0	0	0	0	0	0	0	0	0	0	0	0	0	0	0	0
Chlorobiales	0	0	0	0	0	0	0	0	11	0	0	0	0	0	0	0	0	0	0	0	0	0	0	0	0
Corynebacteriaceae	0	0	0	0	0	0	0	0	0	0	0	0	0	0	7	3	0	0	0	0	3	0	0	0	8
Mycobacteriaceae	0	0	0	0	0	13	0	0	0	0	0	0	0	0	0	1	0	0	0	0	0	0	0	0	7
Cyanobacteria	0	0	0	0	0	0	0	0	0	0	0	0	0	13	0	11	0	1	0	0	0	0	0	0	0
Chloroflexi	0	0	0	0	0	0	0	0	0	0	0	0	0	0	0	5	0	0	0	0	0	0	0	0	25
Deinococcus-Therm	0	0	0	0	0	0	0	0	0	0	0	0	0	0	0	4	0	0	0	0	0	0	0	0	10
Thermotogales	0	0	0	0	0	0	0	0	0	0	0	0	0	0	0	11	10	4	0	0	3	1	0	0	0
Desulfovibrionales	0	0	0	0	0	0	0	0	0	0	0	0	0	0	0	0	0	0	0	0	0	0	0	0	0
Caulobacterales	0	0	0	0	0	0	0	0	0	0	4	0	0	0	0	0	0	0	0	0	0	0	0	0	0
Rhodobacterales	0	0	0	0	0	0	14	13	0	0	14	0	0	0	9	0	0	0	0	0	0	0	0	0	0
Rhizobiales	0	0	0	0	0	0	25	0	0	0	14	12	8	0	0	0	0	0	0	0	5	0	0	0	0
Burkholderia	0	0	0	8	0	0	0	0	0	0	0	0	0	0	0	0	0	0	0	0	0	0	0	0	0
Ralstonia	0	0	0	6	0	0	0	0	0	0	0	0	0	0	0	0	0	0	0	0	0	0	0	0	0
Enterobacteriales	0	0	0	0	5	0	0	0	0	0	0	0	0	0	12	0	0	0	0	0	12	12	12	12	0
Pasteurellales	0	0	0	0	0	0	0	0	0	0	0	0	0	0	10	3	0	0	0	0	0	9	5	7	0
Vibrionales	0	0	0	0	0	0	0	0	0	0	0	0	0	0	9	3	0	0	0	0	10	10	10	10	0
Pseudomonadaceae	0	0	0	0	0	0	0	0	0	0	0	0	0	0	8	0	0	0	0	0	0	0	0	0	0
Shewanella	0	0	0	0	0	0	0	0	0	0	0	0	0	0	16	0	0	0	0	0	16	16	16	16	0
TOTAL	23	10	17	14	5	13	39	13	11	13	32	12	8	13	98	108	75	67	61	43	49	48	43	45	1134
number of regulogs	3	1	2	2	1	1	2	1	1	1	3	1	1	1	10	13	6	7	5	4	6	5	4	4	9

*T-boxes	Geno-mes	Ala	Arg	Asn	Asp	Cys	Gly	His	Ile	Leu	Lys	Met	Phe	Pro	Ser	Thr	Trp	Tyr	Val
Lactobacillaceae	15	15	14	13	16	1	15	4	28	16	1	54	15	3	15	16	15	29	21
Streptococcaceae	15	15	0	3	2	0	15	7	15	0	0	2	15	0	15	15	24	0	15
Bacillales	11	10	2	8	5	11	9	0	16	29	7	6	11	21	13	20	17	19	10
Staphylococcaceae	7	7	7	7	0	7	7	0	7	7	0	8	6	0	7	9	6	8	6
Clostridiaceae	20	20	21	24	4	21	10	3	29	30	1	19	22	16	16	22	41	16	22
Bacteroidaceae	11	0	0	0	0	0	0	0	0	0	0	0	0	0	0	0	0	0	0
Chlorobiales	11	0	0	0	0	0	0	0	0	0	0	0	0	0	0	0	0	0	0
Corynebacteriaceae	8	0	0	0	0	0	0	0	8	0	0	0	0	0	0	0	0	0	0
Mycobacteriaceae	9	0	0	0	0	0	0	0	7	0	0	0	0	0	0	0	0	0	0
Cyanobacteria	14	0	0	0	0	0	0	0	0	0	0	0	0	0	0	0	0	0	0
Chloroflexi	5	0	0	0	0	0	5	0	5	0	0	0	2	1	2	4	5	0	1
Deinococcus-Therm	5	0	0	0	0	0	3	0	5	0	0	0	0	0	0	0	0	0	2
Thermotogales	11	0	0	0	0	0	0	0	0	0	0	0	0	0	0	0	0	0	0
Desulfovibrionales	10	0	0	0	0	0	0	0	0	0	0	0	0	0	0	0	0	0	0
Caulobacterales	4	0	0	0	0	0	0	0	0	0	0	0	0	0	0	0	0	0	0
Rhodobacterales	15	0	0	0	0	0	0	0	0	0	0	0	0	0	0	0	0	0	0
Rhizobiales	15	0	0	0	0	0	0	0	0	0	0	0	0	0	0	0	0	0	0
Burkholderia	8	0	0	0	0	0	0	0	0	0	0	0	0	0	0	0	0	0	0
Ralstonia	6	0	0	0	0	0	0	0	0	0	0	0	0	0	0	0	0	0	0
Enterobacteriales	12	0	0	0	0	0	0	0	0	0	0	0	0	0	0	0	0	0	0
Pasteurellales	9	0	0	0	0	0	0	0	0	0	0	0	0	0	0	0	0	0	0
Vibrionales	10	0	0	0	0	0	0	0	0	0	0	0	0	0	0	0	0	0	0
Pseudomonadaceae	8	0	0	0	0	0	0	0	0	0	0	0	0	0	0	0	0	0	0
Shewanella	16	0	0	0	0	0	0	0	0	0	0	0	0	0	0	0	0	0	0

Note: riboswitches are arranged according to abundance and substrate types.
Number of lineages indicates the number of taxa in which a riboswitch can be found.
