# Supplementary material for: Comparative genomics of metabolic capacities of regulons controlled by cis-regulatory RNA motifs in bacteria
Source: BMC Genomics. 2013 Sep 2;14:597. doi: 10.1186/1471-2164-14-597 (PMC3766115; doi:10.1186/1471-2164-14-597)
Supplement: Additional file 4 — Total number of genes controlled by predicted RNA motif regulons in 24 taxonomic groups of Bacteria. Riboswitches are arranged in six groups according to abundance and substrate types. [file 1471-2164-14-597-S4.pdf]

Additional File 4. Total number of genes controlled by predicted RNA motif regulons in 24 taxonomic groups of Bacteria.

Taxonomic group (number of genomes)	Group A - Widely distributed RNA motifs					Group B Moderately distributed RNA motifs													Group C Phylogenetically restrictive RNA motifs														Group D Ribosomal operon leaders						Group E - a.a. operon leaders				Group F T-boxes	
	TPP	Cobalamin	Glycine	FMN	ybbP-ykoY	SAM	Lysine	Purine	PyrR	GEMM	MOCO	PreQ1	ydaO-yuaA	mini-ykkC	ykkC-yxkD	glmS	ykoK	SAH	THF	ylbH	preQ1-II	sucA	Mg sensor	SAM-IV	SAM alpha	SAM-SAH	SAM Chlorobi	Smk box	serC	speF	ybhL	glnA	S15	L10	L20	L21	L19	L13	Trp	His	Leu	Thr	All T-boxes	Total genes
Lactobacillaceae (15)	100	1	0	34	13	2	74	25	122	0	0	13	0	0	3	1	15	0	8	0	3	0	0	0	0	0	0	13	0	0	0	0	9	30	45	39	14	26	0	0	0	0	484	1074
Streptococcaceae (15)	62	0	12	42	16	0	4	24	122	0	0	4	0	0	0	0	6	0	5	0	14	0	0	0	0	0	0	0	0	0	0	0	15	30	45	38	13	30	0	0	0	0	399	881
Bacillales (11)	143	107	30	50	18	242	23	181	109	20	0	46	35	0	19	11	10	0	0	20	0	0	0	0	0	0	0	0	0	0	0	0	0	22	27	33	10	16	0	0	0	0	353	1525
Staphylococcus (7)	86	0	22	35	7	66	63	28	57	0	0	27	0	0	2	7	0	0	0	0	0	0	0	0	0	0	0	0	0	0	0	0	0	14	21	17	7	14	0	0	0	0	174	647
Clostridiaceae (20)	155	392	83	80	38	131	62	70	115	63	33	52	19	0	16	17	14	0	11	0	0	0	0	0	0	0	0	0	0	0	0	0	3	37	55	45	17	0	0	0	0	0	528	2036
Bacteroidaceae (11)	95	239	0	0	0	0	0	0	0	0	0	0	0	0	0	0	0	0	0	0	0	0	0	0	0	0	0	0	0	0	0	0	0	0	0	0	0	0	0	0	0	0	0	334
Chlorobiales (11)	16	198	0	2	0	24	0	0	0	0	1	0	0	0	0	0	0	0	0	0	0	0	0	0	0	0	22	0	0	0	0	0	0	0	0	0	0	0	0	0	0	0	0	263
Corynebacteriaceae (8)	80	17	6	3	11	1	0	0	36	0	0	0	5	0	0	0	1	0	0	0	0	0	0	0	0	0	0	0	0	0	0	0	7	6	0	0	0	0	13	0	0	0	8	194
Mycobacteriaceae (9)	53	44	15	0	13	0	0	0	50	0	0	0	9	0	3	0	14	6	0	0	0	0	0	31	0	0	0	0	0	0	0	0	0	2	0	0	0	0	0	0	0	0	7	247
Cyanobacteria (14)	14	43	0	0	4	2	0	0	0	6	0	0	13	1	29	0	0	0	0	0	0	0	0	0	0	0	0	0	0	0	0	15	0	21	0	2	0	0	0	0	0	0	0	150
Chloroflexi (5)	43	109	15	20	5	43	0	0	10	0	0	0	0	0	0	5	0	0	0	0	0	0	0	0	0	0	0	0	0	0	0	0	0	10	0	0	0	0	0	0	0	0	40	300
Deinococcus-Thermus (5)	44	96	0	15	8	21	0	0	8	3	3	0	0	0	0	3	0	0	0	0	0	0	0	0	0	0	0	0	0	0	0	0	0	8	0	0	0	0	0	0	0	0	12	221
Thermotogales (11)	47	114	0	37	1	3	70	16	0	0	11	0	0	0	0	0	0	0	0	0	0	0	0	0	0	0	0	0	0	0	0	0	0	22	30	23	0	0	2	3	0	0	0	379
Desulfovibrionales (10)	57	67	6	10	7	4	0	0	0	6	0	0	1	1	0	0	0	0	0	0	0	0	0	0	0	0	0	0	0	0	0	0	0	0	0	0	0	0	0	0	0	0	0	159
Caulobacterales (4)	4	28	20	1	4	0	0	0	0	0	1	0	0	4	0	0	0	0	0	0	0	0	0	0	0	0	0	0	8	0	0	0	0	0	0	0	0	0	0	0	0	0	0	70
Rhodobacterales (15)	132	170	45	7	1	0	0	0	0	0	1	4	0	10	8	0	0	0	0	0	0	0	0	0	16	13	0	0	27	0	0	0	9	0	0	0	0	0	0	0	0	0	0	443
Rhizobiales (15)	100	159	57	12	3	0	0	0	0	0	0	0	0	27	40	0	0	0	0	0	0	0	0	0	30	0	0	0	28	12	8	0	0	0	0	0	0	0	10	0	0	0	0	486
Burkholderia (8)	41	140	38	9	12	0	0	0	0	0	0	0	0	10	21	0	0	24	0	0	0	24	0	0	0	0	0	0	0	0	0	0	0	0	0	0	0	0	0	0	0	0	0	319
Ralstonia (6)	30	45	30	6	8	0	0	0	0	1	0	0	0	3	8	0	0	24	0	0	0	18	0	0	0	0	0	0	0	0	0	0	0	0	0	0	0	0	0	0	0	0	0	173
Enterobacteriales (12)	130	99	1	12	21	0	5	0	0	1	53	0	0	10	5	0	5	0	0	0	0	0	5	0	0	0	0	0	0	0	0	0	12	0	0	0	0	0	67	92	49	34	0	601
Pasteurellales (9)	59	0	10	18	0	0	10	0	0	0	39	24	0	0	0	0	0	0	0	0	0	0	0	0	0	0	0	0	0	0	0	0	10	6	0	0	0	0	0	48	15	19	0	258
Vibrionales (10)	136	39	10	8	22	0	22	6	0	15	46	0	0	4	0	0	1	0	0	0	0	0	0	0	0	0	0	0	0	0	0	0	9	6	0	0	0	0	62	80	37	30	0	533
Pseudomonadaceae (8)	14	156	1	16	11	0	0	0	0	0	0	0	0	9	19	0	0	14	0	0	0	0	0	0	0	0	0	0	0	0	0	0	8	0	0	0	0	0	0	0	0	0	0	248
Shewanella (16)	155	156	14	15	19	0	48	7	0	20	119	0	0	7	0	0	0	0	0	0	0	0	0	0	0	0	0	0	0	0	0	0	16	0	0	0	0	0	96	126	64	48	0	910
Total	1796	2419	415	432	242	539	381	357	629	135	307	170	82	86	173	44	66	68	24	20	17	42	5	31	46	13	22	13	63	12	8	15	98	214	223	197	61	86	250	349	165	131	2005	12451
