# Supplementary material for: Comparative genomics of metabolic capacities of regulons controlled by cis-regulatory RNA motifs in bacteria
Source: BMC Genomics. 2013 Sep 2;14:597. doi: 10.1186/1471-2164-14-597 (PMC3766115; doi:10.1186/1471-2164-14-597)
Supplement: Additional file 6 — Distribution of Specific Functional Categories regulated by each RNA motif family across 24 analyzed taxonomic groups. SFC number for individual riboswitch family is listed in each row, with averaged proportion listed in the last column; ‘per genome’ values and listed at the bottom. [file 1471-2164-14-597-S6.pdf]

Additional File 6. Distribution of Specific Functional Categories regulated by each RNA family across 24 analyzed taxonomic groups.

RNA motif	SFC*	Total # of SFC per	Lactobacillaceae	Streptococcaceae	Bacillales	Staphylococcus	Clostridiaceae	Bacteroidaceae	Chlorobiales	Corynebacteriacea	Mycobacteriaceae	Cyanobacteria	Chloroflexi	Deinococcus-Therm	Thermotogales	Desulfovibrionales	Caulobacterales	Rhodobacterales	Rhizobiales	Burkholderia	Ralstonia	Enterobacteriales	Pasteurellales	Vibrionales	Pseudomonadacea	Shewanella
Genome count:		255	15	15	11	7	20	11	11	8	9	14	5	5	11	10	4	15	15	8	6	12	9	10	8	16
Cobalamin	B12 biosynthesis	1027	0	0	57	0	197	19	72	0	2	13	57	52	53	20	12	47	47	93	25	63	0	11	93	94
	B12 & precursor transporters	589	0	0	20	0	51	86	56	9	13	10	17	18	30	26	11	50	40	25	18	12	0	16	29	52
	Cobalt transporters	183	0	0	8	0	51	4	12	4	10	6	11	4	8	0	0	16	16	8	1	11	0	0	8	5
	Isozymes of B12-dependent enzymes	136	0	0	12	0	1	32	13	2	6	3	3	6	0	15	2	13	28	0	0	0	0	0	0	0
	Miscellaneous	484	1	0	10	0	92	98	45	2	13	11	21	16	23	6	3	44	28	14	1	13	0	12	26	5
	TOTAL	2419	1	0	107	0	392	239	198	17	44	43	109	96	114	67	28	170	159	140	45	99	0	39	156	156
TPP	Thiamine biosynthesis	1145	35	24	85	60	105	76	16	53	44	14	14	30	27	47	4	70	61	39	28	89	24	85	9	106
	Thiamine & precursor transporters	553	53	32	46	22	41	14	0	18	9	0	22	10	11	9	0	59	33	0	0	41	32	51	2	48
	Miscellaneous	98	12	6	12	4	9	5	0	9	0	0	7	4	9	1	0	3	6	2	2	0	3	0	3	1
	TOTAL	1796	100	62	143	86	155	95	16	80	53	14	43	44	47	57	4	132	100	41	30	130	59	136	14	155
FMN	Riboflavin biosynthesis	332	20	16	41	28	57	0	2	0	0	0	10	15	32	10	1	6	10	9	6	12	18	8	16	15
	Riboflavin transporters	78	14	15	9	7	20	0	0	3	0	0	10	0	0	0	0	0	0	0	0	0	0	0	0	0
	Miscellaneous	22	0	11	0	0	3	0	0	0	0	0	0	0	5	0	0	1	2	0	0	0	0	0	0	0
	TOTAL	432	34	42	50	35	80	0	2	3	0	0	20	15	37	10	1	7	12	9	6	12	18	8	16	15
Lysine	Lysine biosynthesis	272	52	2	11	54	40	0	0	0	0	0	0	0	69	0	0	0	0	0	0	5	2	9	0	28
	Lysine transporters	104	22	2	12	7	20	0	0	0	0	0	0	0	0	0	0	0	0	0	0	0	8	13	0	20
	Miscellaneous	5	0	0	0	2	2	0	0	0	0	0	0	0	1	0	0	0	0	0	0	0	0	0	0	0
	TOTAL	381	74	4	23	63	62	0	0	0	0	0	0	0	70	0	0	0	0	0	0	5	10	22	0	48
SAM	Methionine biosynthesis	157	0	0	54	8	38	0	24	0	0	0	17	16	0	0	0	0	0	0	0	0	0	0	0	0
	Methionine & SAM recycling	98	2	0	51	12	22	0	0	0	0	2	9	0	0	0	0	0	0	0	0	0	0	0	0	0
	Cysteine biosynthesis	51	0	0	40	0	7	0	0	0	0	0	4	0	0	0	0	0	0	0	0	0	0	0	0	0
	Methionine transporters	140	0	0	57	36	39	0	0	0	0	0	0	5	3	0	0	0	0	0	0	0	0	0	0	0
	Miscellaneous	93	0	0	40	10	25	0	0	1	0	0	13	0	0	4	0	0	0	0	0	0	0	0	0	0
	TOTAL	539	2	0	242	66	131	0	24	1	0	2	43	21	3	4	0	0	0	0	0	0	0	0	0	0
Glycine	Glycine metabolism	271	0	0	27	22	49	0	0	6	13	0	12	0	0	2	16	37	46	23	18	0	0	0	0	0
	Serine metabolism	16	0	0	0	0	2	0	0	0	2	0	0	0	0	0	0	0	0	6	6	0	0	0	0	0
	Glycine transporters	60	0	12	3	0	10	0	0	0	0	0	0	0	0	2	0	0	0	0	0	0	9	10	0	14
	Miscellaneous	68	0	0	0	0	22	0	0	0	0	0	3	0	0	2	4	8	11	9	6	1	1	0	1	0
	TOTAL	415	0	12	30	22	83	0	0	6	15	0	15	0	0	6	20	45	57	38	30	1	10	10	1	14
Purine	Purine metabolism	267	9	11	149	21	51	0	0	0	0	0	0	0	16	0	0	0	0	0	0	0	0	5	0	5
	Purine & precursor transporters	84	16	13	28	7	17	0	0	0	0	0	0	0	0	0	0	0	0	0	0	0	0	1	0	2
	Miscellaneous	6	0	0	4	0	2	0	0	0	0	0	0	0	0	0	0	0	0	0	0	0	0	0	0	0
	TOTAL	357	25	24	181	28	70	0	0	0	0	0	0	0	16	0	0	0	0	0	0	0	0	6	0	7
SAH	Methionine biosynthesis	26	0	0	0	0	0	0	0	0	6	0	0	0	0	0	0	0	0	8	6	0	0	0	6	0
	Methionine & SAM recycling	21	0	0	0	0	0	0	0	0	0	0	0	0	0	0	0	0	0	8	6	0	0	0	7	0
	Miscellaneous	21	0	0	0	0	0	0	0	0	0	0	0	0	0	0	0	0	0	8	12	0	0	0	1	0
	TOTAL	68	0	0	0	0	0	0	0	0	6	0	0	0	0	0	0	0	0	24	24	0	0	0	14	0
SAM-IV	Methionine biosynthesis	16	0	0	0	0	0	0	0	0	16	0	0	0	0	0	0	0	0	0	0	0	0	0	0	0
	Miscellaneous	15	0	0	0	0	0	0	0	0	15	0	0	0	0	0	0	0	0	0	0	0	0	0	0	0
	TOTAL	31	0	0	0	0	0	0	0	0	31	0	0	0	0	0	0	0	0	0	0	0	0	0	0	0
SAM-Alpha	Methionine biosynthesis	46	0	0	0	0	0	0	0	0	0	0	0	0	0	0	0	16	30	0	0	0	0	0	0	0
	Miscellaneous	0	0	0	0	0	0	0	0	0	0	0	0	0	0	0	0	0	0	0	0	0	0	0	0	0
	TOTAL	46	0	0	0	0	0	0	0	0	0	0	0	0	0	0	0	16	30	0	0	0	0	0	0	0
SAM-Chlorobi	Methionine & SAM recycling	22	0	0	0	0	0	0	22	0	0	0	0	0	0	0	0	0	0	0	0	0	0	0	0	0
	Miscellaneous	0	0	0	0	0	0	0	0	0	0	0	0	0	0	0	0	0	0	0	0	0	0	0	0	0
	TOTAL	22	0	0	0	0	0	0	22	0	0	0	0	0	0	0	0	0	0	0	0	0	0	0	0	0
SAM-SAH	Methionine & SAM recycling	13	0	0	0	0	0	0	0	0	0	0	0	0	0	0	0	13	0	0	0	0	0	0	0	0
	Miscellaneous	0	0	0	0	0	0	0	0	0	0	0	0	0	0	0	0	0	0	0	0	0	0	0	0	0
	TOTAL	13	0	0	0	0	0	0	0	0	0	0	0	0	0	0	0	13	0	0	0	0	0	0	0	0
SMK-box	Methionine & SAM recycling	13	13	0	0	0	0	0	0	0	0	0	0	0	0	0	0	0	0	0	0	0	0	0	0	0
	Miscellaneous	0	0	0	0	0	0	0	0	0	0	0	0	0	0	0	0	0	0	0	0	0	0	0	0	0
	TOTAL	13	13	0	0	0	0	0	0	0	0	0	0	0	0	0	0	0	0	0	0	0	0	0	0	0
preQ1	Queuosine biosynthesis	108	0	0	42	21	17	0	0	0	0	0	0	0	0	0	0	4	0	0	0	0	24	0	0	0
	Queuosine & precursor transporters	40	9	2	4	6	19	0	0	0	0	0	0	0	0	0	0	0	0	0	0	0	0	0	0	0
	Miscellaneous	22	4	2	0	0	16	0	0	0	0	0	0	0	0	0	0	0	0	0	0	0	0	0	0	0
	TOTAL	170	13	4	46	27	52	0	0	0	0	0	0	0	0	0	0	4	0	0	0	0	24	0	0	0
preQ1-II	Queuosine & precursor transporters	17	3	14	0	0	0	0	0	0	0	0	0	0	0	0	0	0	0	0	0	0	0	0	0	0
	Miscellaneous	0	0	0	0	0	0	0	0	0	0	0	0	0	0	0	0	0	0	0	0	0	0	0	0	0
	TOTAL	17	3	14	0	0	0	0	0	0	0	0	0	0	0	0	0	0	0	0	0	0	0	0	0	0
THF	Folate biosynthesis	1	0	1	0	0	0	0	0	0	0	0	0	0	0	0	0	0	0	0	0	0	0	0	0	0
	Folate transporters	23	8	4	0	0	11	0	0	0	0	0	0	0	0	0	0	0	0	0	0	0	0	0	0	0
	Miscellaneous	0	0	0	0	0	0	0	0	0	0	0	0	0	0	0	0	0	0	0	0	0	0	0	0	0
	TOTAL	24	8	5	0	0	11	0	0	0	0	0	0	0	0	0	0	0	0	0	0	0	0	0	0	0
MOCO	Molybdenum cofactor biosynthesis	237	0	0	0	0	24	0	1	0	0	0	0	0	6	0	1	1	0	0	0	53	36	44	0	71
	Molybdenum & tungsten transporters	58	0	0	0	0	4	0	0	0	0	0	0	3	3	0	0	0	0	0	0	0	0	0	0	48
	Miscellaneous	12	0	0	0	0	5	0	0	0	0	0	0	0	2	0	0	0	0	0	0	0	3	2	0	0
	TOTAL	307	0	0	0	0	33	0	1	0	0	0	0	3	11	0	1	1	0	0	0	53	39	46	0	119
glmS	Aminosugar biosynthesis	44	1	0	11	7	17	0	0	0	0	0	5	3	0	0	0	0	0	0	0	0	0	0	0	0
	Miscellaneous	0	0	0	0	0	0	0	0	0	0	0	0	0	0	0	0	0	0	0	0	0	0	0	0	0
	TOTAL	44	1	0	11	7	17	0	0	0	0	0	5	3	0	0	0	0	0	0	0	0	0	0	0	0
Mg sensor	Magnesium transporters	5	0	0	0	0	0	0	0	0	0	0	0	0	0	0	0	0	0	0	0	5	0	0	0	0
	Miscellaneous	0	0	0	0	0	0	0	0	0	0	0	0	0	0	0	0	0	0	0	0	0	0	0	0	0
	TOTAL	5	0	0	0	0	0	0	0	0	0	0	0	0	0	0	0	0	0	0	0	5	0	0	0	0
GEMM	Polysaccharide degradation	14	0	0	0	0	0	0	0	0	0	0	0	3	0	0	0	0	0	0	0	0	0	4	0	7
	Miscellaneous	121	0	0	20	0	63	0	0	0	0	6	0	0	0	6	0	0	0	0	1	1	0	11	0	13
	TOTAL	135	0	0	20	0	63	0	0	0	0	6	0	3	0	6	0	0	0	0	1	1	0	15	0	20
PyrR	Pyrimidine metabolism	602	117	120	107	52	113	0	0	36	43	0	8	6	0	0	0	0	0	0	0	0	0	0	0	0
	Pyrimidine transporters	7	5	0	0	0	2	0	0	0	0	0	0	0	0	0	0	0	0	0	0	0	0	0	0	0
	Miscellaneous	20	0	2	2	5	0	0	0	0	7	0	2	2	0	0	0	0	0	0	0	0	0	0	0	0
	TOTAL	629	122	122	109	57	115	0	0	36	50	0	10	8	0	0	0	0	0	0	0	0	0	0	0	0
sucA	Citric acid cycle	42	0	0	0	0	0	0	0	0	0	0	0	0	0	0	0	0	0	24	18	0	0	0	0	0
	Miscellaneous	0	0	0	0	0	0	0	0	0	0	0	0	0	0	0	0	0	0	0	0	0	0	0	0	0
	TOTAL	42	0	0	0	0	0	0	0	0	0	0	0	0	0	0	0	0	0	24	18	0	0	0	0	0
serC	Serine metabolism	63	0	0	0	0	0	0	0	0	0	0	0	0	0	0	8	27	28	0	0	0	0	0	0	0
	Miscellaneous	0	0	0	0	0	0	0	0	0	0	0	0	0	0	0	0	0	0	0	0	0	0	0	0	0
	TOTAL	63	0	0	0	0	0	0	0	0	0	0	0	0	0	0	8	27	28	0	0	0	0	0	0	0
speF	Ornithine degradation	12	0	0	0	0	0	0	0	0	0	0	0	0	0	0	0	0	12	0	0	0	0	0	0	0
	Miscellaneous	0	0	0	0	0	0	0	0	0	0	0	0	0	0	0	0	0	0	0	0	0	0	0	0	0
	TOTAL	12	0	0	0	0	0	0	0	0	0	0	0	0	0	0	0	0	12	0	0	0	0	0	0	0
ydaO-yuaA	Potassium transporters	21	0	0	21	0	0	0	0	0	0	0	0	0	0	0	0	0	0	0	0	0	0	0	0	0
	Miscellaneous	61	0	0	14	0	19	0	0	5	9	13	0	0	0	1	0	0	0	0	0	0	0	0	0	0
	TOTAL	82	0	0	35	0	19	0	0	5	9	13	0	0	0	1	0	0	0	0	0	0	0	0	0	0
ykkC-yxkD	Urea and agmatine utilization	122	0	0	0	0	0	0	0	0	0	26	0	0	0	0	0	4	40	21	7	5	0	0	19	0
	Multidrug resistance transporter	25	2	0	16	2	5	0	0	0	0	0	0	0	0	0	0	0	0	0	0	0	0	0	0	0
	Miscellaneous	26	1	0	3	0	11	0	0	0	3	3	0	0	0	0	0	4	0	0	1	0	0	0	0	0
	TOTAL	173	3	0	19	2	16	0	0	0	3	29	0	0	0	0	0	8	40	21	8	5	0	0	19	0
mini-ykkC	Urea and agmatine utilization	28	0	0	0	0	0	0	0	0	0	0	0	0	0	0	0	0	20	2	2	1	0	0	3	0
	Multidrug resistance transporter	51	0	0	0	0	0	0	0	0	0	1	0	0	0	1	4	3	7	8	1	9	0	4	6	7
	Miscellaneous	7	0	0	0	0	0	0	0	0	0	0	0	0	0	0	0	7	0	0	0	0	0	0	0	0
	TOTAL	86	0	0	0	0	0	0	0	0	0	1	0	0	0	1	4	10	27	10	3	10	0	4	9	7
ykoK	Magnesium transporters	37	6	5	8	0	11	0	0	1	0	0	0	0	0	0	0	0	0	0	0	5	0	1	0	0
	Miscellaneous	29	9	1	2	0	3	0	0	0	14	0	0	0	0	0	0	0	0	0	0	0	0	0	0	0
	TOTAL	66	15	6	10	0	14	0	0	1	14	0	0	0	0	0	0	0	0	0	0	5	0	1	0	0
L10 leader	Ribosome biogenesis	214	30	30	22	14	37	0	0	6	2	21	10	8	22	0	0	0	0	0	0	0	6	6	0	0
	Miscellaneous	0	0	0	0	0	0	0	0	0	0	0	0	0	0	0	0	0	0	0	0	0	0	0	0	0
	TOTAL	214	30	30	22	14	37	0	0	6	2	21	10	8	22	0	0	0	0	0	0	0	6	6	0	0
L13 leader	Ribosome biogenesis	86	26	30	16	14	0	0	0	0	0	0	0	0	0	0	0	0	0	0	0	0	0	0	0	0
	Miscellaneous	0	0	0	0	0	0	0	0	0	0	0	0	0	0	0	0	0	0	0	0	0	0	0	0	0
	TOTAL	86	26	30	16	14	0	0	0	0	0	0	0	0	0	0	0	0	0	0	0	0	0	0	0	0
L19 leader	Ribosome biogenesis	61	14	13	10	7	17	0	0	0	0	0	0	0	0	0	0	0	0	0	0	0	0	0	0	0
	Miscellaneous	0	0	0	0	0	0	0	0	0	0	0	0	0	0	0	0	0	0	0	0	0	0	0	0	0
	TOTAL	61	14	13	10	7	17	0	0	0	0	0	0	0	0	0	0	0	0	0	0	0	0	0	0	0
L20 leader	Ribosome biogenesis	223	45	45	27	21	55	0	0	0	0	0	0	0	30	0	0	0	0	0	0	0	0	0	0	0
	Miscellaneous	0	0	0	0	0	0	0	0	0	0	0	0	0	0	0	0	0	0	0	0	0	0	0	0	0
	TOTAL	223	45	45	27	21	55	0	0	0	0	0	0	0	30	0	0	0	0	0	0	0	0	0	0	0
L21 leader	Ribosome biogenesis	138	30	27	22	11	30	0	0	0	0	2	0	0	16	0	0	0	0	0	0	0	0	0	0	0
	Miscellaneous	59	9	11	11	6	15	0	0	0	0	0	0	0	7	0	0	0	0	0	0	0	0	0	0	0
	TOTAL	197	39	38	33	17	45	0	0	0	0	2	0	0	23	0	0	0	0	0	0	0	0	0	0	0
S15 leader	Ribosome biogenesis	98	9	15	0	0	3	0	0	7	0	0	0	0	0	0	0	9	0	0	0	12	10	9	8	16

	Miscellaneous	**0**	0	0	0	0	0	0	0	0	0	0	0	0	0	0	0	0	0	0	0	0	0	0	0	0
	TOTAL	**98**	9	15	0	0	3	0	0	7	0	0	0	0	0	0	0	9	0	0	0	12	10	9	8	16
**Combined ribosom**	**Ribosome biogenesis**	**820**	**154**	**160**	**97**	**67**	**142**	**0**	**0**	**13**	**2**	**23**	**10**	**8**	**68**	**0**	**0**	**9**	**0**	**0**	**0**	**12**	**16**	**15**	**8**	**16**
	**Miscellaneous**	**59**	**9**	**11**	**11**	**6**	**15**	**0**	**0**	**0**	**0**	**0**	**0**	**0**	**7**	**0**	**0**	**0**	**0**	**0**	**0**	**0**	**0**	**0**	**0**	**0**
	**TOTAL**	**879**	**163**	**171**	**108**	**73**	**157**	**0**	**0**	**13**	**2**	**23**	**10**	**8**	**75**	**0**	**0**	**9**	**0**	**0**	**0**	**12**	**16**	**15**	**8**	**16**
Leu leader	Leucine biosynthesis	**163**	0	0	0	0	0	0	0	0	0	0	0	0	0	0	0	0	0	0	0	48	14	37	0	64
	Miscellaneous	**2**	0	0	0	0	0	0	0	0	0	0	0	0	0	0	0	0	0	0	0	1	1	0	0	0
	TOTAL	**165**	0	0	0	0	0	0	0	0	0	0	0	0	0	0	0	0	0	0	0	49	15	37	0	64
His leader	Histidine biosynthesis	**334**	0	0	0	0	0	0	0	0	0	0	0	0	0	0	0	0	0	0	0	91	37	80	0	126
	Histidine transporters	**6**	0	0	0	0	0	0	0	0	0	0	0	0	3	0	0	0	0	0	0	0	3	0	0	0
	Miscellaneous	**9**	0	0	0	0	0	0	0	0	0	0	0	0	0	0	0	0	0	0	0	1	8	0	0	0
	TOTAL	**349**	0	0	0	0	0	0	0	0	0	0	0	0	3	0	0	0	0	0	0	92	48	80	0	126
Thr leader	Threonine biosynthesis	**131**	0	0	0	0	0	0	0	0	0	0	0	0	0	0	0	0	0	0	0	34	19	30	0	48
	Miscellaneous	**0**	0	0	0	0	0	0	0	0	0	0	0	0	0	0	0	0	0	0	0	0	0	0	0	0
	TOTAL	**131**	0	0	0	0	0	0	0	0	0	0	0	0	0	0	0	0	0	0	0	34	19	30	0	48
Trp leader	Tryptophan biosynthesis	**250**	0	0	0	0	0	0	0	13	0	0	0	0	2	0	0	0	10	0	0	67	0	62	0	96
	Miscellaneous	**0**	0	0	0	0	0	0	0	0	0	0	0	0	0	0	0	0	0	0	0	0	0	0	0	0
	TOTAL	**250**	0	0	0	0	0	0	0	13	0	0	0	0	2	0	0	0	10	0	0	67	0	62	0	96
**Combined amino a**	**Amino acid biosynthesis**	**878**	**0**	**0**	**0**	**0**	**0**	**0**	**0**	**13**	**0**	**0**	**0**	**0**	**2**	**0**	**0**	**0**	**10**	**0**	**0**	**240**	**70**	**209**	**0**	**334**
	**Miscellaneous**	**17**	**0**	**0**	**0**	**0**	**0**	**0**	**0**	**0**	**0**	**0**	**0**	**0**	**3**	**0**	**0**	**0**	**0**	**0**	**0**	**2**	**12**	**0**	**0**	**0**
	**TOTAL**	**895**	**0**	**0**	**0**	**0**	**0**	**0**	**0**	**13**	**0**	**0**	**0**	**0**	**5**	**0**	**0**	**0**	**10**	**0**	**0**	**242**	**82**	**209**	**0**	**334**
T-box	Amino acyl-tRNA synthetases	**970**	222	158	181	94	267	0	0	8	7	0	23	10	0	0	0	0	0	0	0	0	0	0	0	0
	Amino acid biosynthesis	**636**	114	116	144	74	184	0	0	0	0	0	4	0	0	0	0	0	0	0	0	0	0	0	0	0
	Amino acid transporters	**207**	104	32	15	0	56	0	0	0	0	0	0	0	0	0	0	0	0	0	0	0	0	0	0	0
	Miscellaneous	**192**	44	93	13	6	21	0	0	0	0	0	13	2	0	0	0	0	0	0	0	0	0	0	0	0
	TOTAL	**2005**	484	399	353	174	528	0	0	8	7	0	40	12	0	0	0	0	0	0	0	0	0	0	0	0

*SFC number for individual riboswitch family is listed in each row, with averaged proportion listed in the last column; 'per genome' value is listed in the bottom half for each riboswitch class.
