# Supplementary material for: Comparative genomics of metabolic capacities of regulons controlled by cis-regulatory RNA motifs in bacteria
Source: BMC Genomics. 2013 Sep 2;14:597. doi: 10.1186/1471-2164-14-597 (PMC3766115; doi:10.1186/1471-2164-14-597)
Supplement: Additional file 7 — Comparative genomics analysis of functional regulon content for RNA motifs from groups B and C, ribosomal and amino acid operon leaders (groups D and E), and T-boxes (group F). Additional text describes detailed analyses of the remaining RNA motif families that were not included in the main manuscript text. [file 1471-2164-14-597-S7.pdf]

Additional file 7 - Comparative genomics analysis of functional regulon content for RNA motifs from groups B and C, ribosomal and amino acid operon leaders (groups D and E), and T-boxes (group F).

Group B - moderately distributed riboswitches

SAM

The SAM (S-adenosylmethionine) riboswitch regulates mostly genes in the cysteine and methionine metabolic pathways, which directly affects the intracellular balance of SAM cofactor, a major cellular methyl donor. Based on their proportion from all the regulated genes, starting from high to low, the genes regulated by this riboswitch were classified into four SFCs of known functional categories: (i) methionine biosynthesis, (ii) methionine transporters, (iii) methionine and SAM recycling, and (iv) cysteine biosynthesis, which represent 11, 4, 11, and 13 unique functional gene orthologs, respectively (Additional file 5). Together these SFCs can be categorized under two major OFCs of amino acid metabolism and amino acid uptake. SAM riboswitches were found in Corynebacteriaceae, Chloroflexi, Cyanobacteria, numerous genomes of Firmicutes, Desulfovibrionales and Thermotogales (see Additional file 6 and RegPrecise). Altogether we annotated 257 SAM RNA sites that control 539 genes in 68 genomes. On average, these numbers correspond to 7.9 regulated genes per genome or 2.1 genes per riboswitch.

As expected, most of these genes fall into cysteine and methionine metabolic pathway (Figure S1). Of the novel regulated genes found, methylthioribose ABC transport systems are found in some species of Bacillales (see RegPrecise). Among other novel regulated genes found in Bacillales there are N-acyl-L-amino acid amidohydrolase (EC 3.5.1.14), and predicted sulfonate monooxygenase (OB3078). In Clostridiaceae, genes encoding YbaK family protein can be found regulated in two genomes. In Chloroflexi, the SAM-regulated adenosine kinase Adk can be found in three genomes.

Lysine

Lysine riboswitches mainly regulate genes in lysine metabolism. Genes regulated by Lysine riboswitches were categorized into two SFCs of lysine biosynthesis and lysine transporter genes that have 11 and 8 unique functional gene orthologs, respectively (Additional file 5). Together these SFCs can be categorized under two major OFCs of amino acid metabolism and amino acid uptake. Lysine riboswitches were found mainly in Firmicutes, γ-proteobacteria, and Thermotogales (see Additional file 6 and RegPrecise). Altogether there are 186 Lysine RNA sites regulating 381 genes in 102 genomes. On average, these numbers correspond to 3.7 regulated genes per genome or 2.0 genes per riboswitch.

Lysine biosynthesis genes dominate in the SFC category for most of genomes analyzed (Figure S2). However, lysine transporter genes dominate the number of total regulated genes in Pasteurellales. Most of regulated lysine biosynthesis genes occupy the pathway that governs the conversion of small and acidic amino acids to lysine; however, in Enterobacteriales, only *lysC* (encoding an aspartokinase gene) is regulated by lysine riboswitch (see RegPrecise). Most regulated lysine transporters can be classified as either ABC-type (*lysXY* and *Clos_0722-24*) or secondary (*yvsH*, *lysW*, and *lysP*) transporters, and genes for these transporters were found encoded in their own operons separate from the rest of lysine biosynthesis genes.

Purine

The Purine riboswitches control genes important for purine biosynthesis and purine salvage. The regulated genes were classified under two SFCs of purine metabolism and purine and precursor transporters that account for 18 and 6 unique functional gene orthologs each (Additional file 5). These two SFCs were in turn categorized under the OFCs of nucleotide metabolism and nucleotide uptake. Purine riboswitches were found mostly in Firmicutes, certain γ-proteobacteria (Shewanella and Vibrionales), and few Thermotogales (T Additional file 6 and Figure S3; see also RegPrecise). The distribution we had observed agrees with the notion that the Purine riboswitch regulons in γ-proteobacteria might have been the result of horizontal gene transfer events [1]. Altogether we annotated 141 Purine RNA sites that control 357 genes in 68 genomes. On average, these numbers correspond to 5.3 regulated genes per genome or 2.5 genes per riboswitch.

For purine metabolic genes, most of them regulate the conversion of amino acids, pentose phosphate, and thiamin to purine derivatives such as inosine and hypoxanthine while all purine and precursor transporter genes (pbuG, pbuX, nupG, pbuE, and pbuO) encode secondary transporters (see RegPrecise and KEGG database). Although only species under Bacillales have most riboswitch regulation for purine metabolic genes, the purine metabolic pathway appeared to be preserved in other taxa with few purine riboswitches, with exception in *Lactobacillus johnsonii* NCC 533 and *Lactobacillus brevis* ATCC 367.

PyrR

The PyrR binding RNA motif controls the expression of pyrimidine biosynthesis and transport genes. Genes regulated by this RNA motif were categorized under the SFCs of pyrimidine metabolism and pyrimidine transport, which represent 14 and 1 unique functional gene orthologs each (Additional file 5). These SFCs in turn can be categorized under the broader OFCs of nucleotide metabolism and nucleotide uptake. This RNA motif was found in Actinobacteria, Chloroflexi, Thermus-Deinococcus and many genomes of Firmicutes (Additional file 6 and Figure S4; see also RegPrecise). Altogether we counted 211 PyrR RNA sites that

control 629 genes in 79 genomes. On average, these numbers correspond to 8.0 regulated genes per genome or 3.0 genes per RNA site.

Genes responsible for pyrimidine metabolism (*pyrRPBCKDFEGI*, *carAB*, *ntd*, and *codA*) are the dominant SFC and account for nearly 95% of regulated genes. In contrast, pyrimidine transport genes (*codB*) are found only in Clostridiaceae and Lactobacillaceae. From our analysis, no novel function was uncovered for any of regulated genes.

GEMM

The cyclic di-GMP-responsive riboswitches, or GEMM RNA motifs, control genes that were largely responsible for motility, virulence, and biofilm formation. In accordance, many genes we found in the corresponding regulons encode proteins that participate in these functions (categorized as miscellaneous SFC in Figure S5). The only other SFC recognized from this study was responsible for polysaccharide degradation, and four unique functional gene orthologs were recognized under this category (Additional file 5). GEMM riboswitches were found in Cyanobacteria, some Firmicutes, and various Proteobacteria (Additional file 6 and Figure S5; see also RegPrecise). Altogether we annotated 89 GEMM RNA sites that control 135 genes in 50 genomes. On average, these numbers correspond to 2.7 regulated genes per genome or 1.5 genes per riboswitch.

Of all SFCs, polysaccharide degradation genes were found only in Deinococcus-Thermus, Vibrionales, and Shewanella and account for just over 10% of all regulated genes (Figure S5 and Additional file 6). In the miscellaneous SFC, the majority of genes were noted for signaling and inducing motility. For example, *tlpA* (encoding methyl-accepting chemotaxis sensory transducer) can be found in three genomes of Bacillales, as well as genes (*srfAA*, *srfAB*, and *srfAC*) encoding components of surfactin synthetase (see RegPrecise). In some genomes of Clostridiaceae, *cheY* (encoding chemotaxis regulator), *flgBCE* (encoding flagellar components) and *fabH* (encoding 3-oxoacyl-[acyl-carrier-protein] synthase, KASIII (EC 2.3.1.41)) can be found.

MOCO

The MOCO RNA motif mainly regulates the biosynthesis and uptake of molybdenum and tungsten cofactors, which participates in oxygen atom transfer in nitrogen, sulfur, and carbon metabolism [2]. Genes regulated by MOCO RNAs were organized into two major SFCs: molybdenum cofactor biosynthesis and molybdenum and tungsten transporters; these two SFCs account for 8 and 6 unique functional gene orthologs, respectively (Additional file 5). Molybdenum cofactor biosynthesis SFC is included under the broader OFC of coenzyme metabolism, and molybdenum and tungsten transporter SFC is under the OFC of metal homeostasis. MOCO

riboswitches were found mostly in γ-proteobacteria, as well as Clostridiaceae, and Thermotogales (Additional file 6 and Figure S6; see also RegPrecise). Altogether we annotated 62 MOCO RNA sites that control 307 genes in 58 genomes. On average, these numbers correspond to 5.3 regulated genes per genome or 5.0 genes per RNA site.

Of all genes belonging to molybdenum cofactor biosynthesis SFC, *moaABCDE* genes are the most prevalent and are responsible for converting GTP to molybdopterin in two steps (see folate biosynthesis pathway in KEGG database and RegPrecise). In addition, we also uncovered *mobAB* and *moeA* genes that encode hypothetical molybdopterin biosynthesis proteins. Of molybdenum and tungsten transport SFC, *modABC* and *tupABC* genes, which both encode ABC-type transporters, are also present in some genomes for the uptake of molybdenum and tungsten, respectively.

PreQ1

The PreQ1 riboswitches regulates the biosynthesis and uptake of queuosine nucleoside [3,4]. The SFCs annotated for PreQ1 riboswitch include genes responsible for queuosine biosynthesis and queuosine and precursor transport, which account for 4 and 3 unique functional gene orthologs, respectively (Additional file 5). These SFCs were included under the broader OFCs of coenzyme metabolism and coenzyme uptake. PreQ1 riboswitches were present in many genomes of Firmicutes and also some Proteobacteria (Additional file 6 and Figure S7; see also RegPrecise). Altogether we annotated 72 PreQ1 RNA sites that control 170 genes in 51 genomes. On average, these numbers correspond to 3.3 regulated genes per genome or 2.4 genes per riboswitch.

Under the SFC responsible for queuosine biosynthesis, *queCDEF* are the genes most conserved between different organisms although they were found absent in some Firmicutes (Lactobacillaceae and Streptococcaceae). For SFC responsible for queuosine and precursor transport, *ypdP*, *queT*, and *qrtT* were the prevalent transporters, with all of them belonging to the ECF transport system; however, these transporter were not found in Proteobacteria. Novel regulated genes regulated by PreQ1 riboswitches include *iunH* (encoding preQ1-regulated inosine-uridine nucleoside hydrolase (EC 3.2.2.1)), *COG1924* (encoding activator of 2-hydroxyglutaryl-CoA dehydratase), *COG1775* (benzoyl-CoA reductase/2-hydroxyglutaryl-CoA dehydratase subunit), *folE* (GTP cyclohydrolase I (EC 3.5.4.16)), which are regulated in four genomes of Clostridiaceae (see RegPrecise). In addition, *iunH* can also be found in four genomes of Lactobacillaceae and two species of Streptococcaceae.

ydaO-yuaA

The *ydaO-yuaA* RNA motif was first discovered as a regulator of some unknown cellular process although it controls genes primarily responsible for metal uptake [5]. Recently, the *ydaO* motif was shown to function as an ATP-sensing riboswitch [10]. The genes regulated by this riboswitch can be categorized under the SFC of Potassium transporters with 6 unique functional gene orthologs (Additional file 5). The SFC is included under the broader OFC of metal homeostasis. *ydaO-yuaA* riboswitches were present in Corynebacteriaceae, Cyanobacteria, some Firmicutes, and Desulfovibrionales (Additional file 6 and Figure S8; see also RegPrecise). Altogether we annotated 59 *ydaO-yuaA* RNA sites that control 82 genes in 37 genomes. On average, these numbers correspond to 2.2 regulated genes per genome or 1.4 genes per riboswitch.

Beside transporters with known affinity for potassium such as KtrAB and KdpABCD, *ydaO-yuaA* appeared to regulate transporters with wider specificities, as well as other protein with potential roles in osmoregulation. *COG0791* (encoding cell wall-associated hydrolase) and *rpfA* (encoding resuscitation-promoting factor) are regulated in at least two genomes of Corynebacteriaceae (see RegPrecise). *tauCAB* (encoding ABC-type nitrate/sulfonate/bicarbonate transport system) and *COG1055* (Na+/H+ antiporter NhaD and related arsenite permeases) are also found in at least two genomes of Cyanobacteria. *ydaO* (encoding putative metabolite transporter) were regulated in 7 genomes of Bacillales examined. *COG1301* (encoding sodium:dicarboxylate symporter family protein) were regulated in two genomes of Bacillales. *COG3773* (encoding Cell wall hydrolyses involved in spore germination) and lysM/COG3773 (encoding *COG3773* genes fused to an N-terminal peptidoglycan-binding lysin domain) were found in four genomes of Clostridiaceae and *dapB* (Dihydrodipicolinate reductase (EC 1.3.1.26)) in two genomes.

ykkC-yxkD

The *ykkC-yxkD* RNA motif is a conserved regulatory element originally identified in *Bacillus subtilis*. The motif was found to be associated with multidrug resistance genes (*ykkCD*) and an unknown gene (*yxkD*). This RNA motif regulates genes that can be categorized under the SFCs of multidrug resistance transport and urea and agmatine utilization, with 2 and 9 unique functional gene orthologs, respectively (Additional file 5). These SFCs are in turn associated with the miscellaneous OFC and secondary metabolism OFC. From our analysis, the *ykkC-yxkD* RNAs were found in Mycobacteriaceae, Cyanobacteria, many genomes of Firmicutes, and various Proteobacteria (Additional file 6 and Figure S10; see also RegPrecise). Altogether we annotated 58 *ykkC-yxkD* RNA sites that control 173 genes in 46 genomes. On average, these numbers correspond to 3.8 regulated genes per genome or 3.0 genes per RNA site.

This RNA motif regulates genes responsible for multidrug resistance transport (*ykkC* and *ykkD*) or urea and agmatine utilization (*ucaAB, speB, hypAB, uctBPA,* and *amt*). Regulated genes with putative functions include

yxkD (encoding an efflux transporters with 5 to 6 transmembrane segments), which were found in three genomes of Bacillales. *COG3382* and COG731 (encoding putative Fe-S oxidoreductase) were found in at least three genomes of Clostridiaceae.

mini-ykkC

The mini-*ykkC* RNA motif is a putative regulatory element found to be associated with detoxification genes [6]. The SFCs for urea and agmatine utilization and multidrug resistance transporter were identified from our analysis, with 3 and 1 unique functional gene orthologs, respectively (Additional file 5); these two SFCs were categorized under the broader OFCs of secondary metabolism and miscellaneous. This RNA motif was found in the genomes of various Proteobacteria and in the cyanobacterum *Gloeobacter violaceus* (Additional file 6 and Figure S9; see also RegPrecise). Altogether we annotated 67 mini-*ykkC* RNA sites that control 86 genes in 61 genomes. On average, these numbers correspond to 1.4 regulated genes per genome or 1.3 genes per RNA site.

This RNA motif regulates a subset of genes controlled by *ykkC-yxkD* riboswitch, and these genes can be categorized under multidrug resistance transporter (*ykkC*) or urea and agmatine utilization (*ucaAB* and *amt*). However, no novel function was uncovered for mini-*ykkC* regulated genes.

glmS

The glmS riboswitch regulates the expression of *glmS* gene, which is responsible for the production of glucosamine-6-phosphate (GlcN6P) [5]. This riboswitch acts as a ribozyme and self-cleaves upon binding to its native ligand GlcN6P [7]. Genes regulated by glmS riboswitch were categorized under the SFC of aminosugar biosynthesis, with *glmS* being the sole gene ortholog (Additional file 5); this SFC is included under the broader OFC of secondary metabolism. glmS riboswitches were found in Chloroflexi, Deinococcus-Thermus, and many genomes of Firmicutes (Additional file 6 and Figure S11; see also RegPrecise). Altogether we annotated 44 *glms* RNA sites that control 44 genes in 44 genomes. On average, these numbers correspond to 1.0 regulated genes per genome or 1.0 genes per riboswitch. There was no novel regulated gene associated with this riboswitch from our analysis.

ykoK

The *ykoK* RNA motif regulates the expression of a diverse range of metal ion transporters. The genes regulated by the *ykoK* RNAs primarily belong to the magnesium transporters SFC, with 3 unique functional gene orthologs identified (Additional file 5). This SFC is in turn assigned to the broader OFC of metal homeostasis. The *ykoK* RNA motif is mostly restricted to Gram-positive organisms and few genomes of

Enterobacteriales (Additional file 6 and Figure S12; see also RegPrecise). Altogether we annotated 48 *ykoK* RNA sites that control 66 genes in 39 genomes. On average, these numbers correspond to 1.7 regulated genes per genome or 1.4 genes per RNA site.

The most widely distributed transporters such as *mgtE* were found in both Gram-positive organisms and γ-proteobacteria, and *mgtB* were found in Firmicutes (see RegPrecise). Less abundant are *corA* and *mntH*-like magnesium transporters, and they were restricted to Clostridiaceae and Mycobacteriaceae. Still, some hypothetical magnesium transporters are regulated by the RNA motif such as *sapB* in *Bacillus halodurans* C-125. SapB, which contains 4 to 6 transmembrane segments, is homologous to the existing MgtC, a component of a P-type ATPase required for magnesium uptake (Alix and Blanc-Potard, 2007). In contrast to known transporter, a large proportion of genes with unknown function are regulated in Lactobacillaceae (around 60%), and in Mycobacteriaceae they represent the entirety of *ykoK*-regulated genes. As shown in RegPredict, COG0474 encodes a P-type ATPase while lp_2077 and lp_2076 encode components of an ABC-type anion transport system (with 10 to 12 transmembrane segments in the permease complex) in Lactobacillaceae. In addition to these hypothetical magnesium transporters, cell envelope-associated transcriptional regulator (LytR) genes were regulated in two species of Lactobacillaceae, and genes encoding paralogues of PE/PPE proteins that contribute to modulation of host immune response can be found in some species of Mycobacteriaceae.

SAH

The SAH (S-adenosylhomocysteine) riboswitches regulate genes responsible for methionine metabolism [6]. The genes regulated by this riboswitch can be categorized under the SFCs for methionine biosynthesis and methionine and SAM recycling, which include 2 and 1 unique functional gene orthologs, respectively (Additional file 5). Both SFCs are assigned to the broader OFC of amino acid metabolism. SAH riboswitches were found in some genomes of β-proteobacteria, Mycobacteriaceae, and Pseudomonadaceae (Additional file 6 and Figure S13; see also RegPrecise). Altogether we annotated 27 SAH riboswitch sites that control 68 genes in 27 genomes. On average, these numbers correspond to 2.5 regulated genes per genome or 2.5 genes per riboswitch.

Like other riboswitches with specificities for SAM or SAH, this riboswitch regulates genes responsible for methionine biosynthesis (*metF, metH*) and methionine and SAM recycling (*ahcY*). Novel regulated genes found for this regulatory element include PF04020 (encoding predicted membrane protein with high similarity to Mycobacterial 4 TMS Phage Holin (MP4 Holin) Family; see TCDB [11]) and PF04993 (encoding regulator of competence-specific genes with TfoX N-terminal domain), which were found in all *Ralstonia* genomes.

Group C – phylogenetically restrictive RNA motifs

A total of 223 sites from 14 Rfam families were uncovered in our analysis (Table 1). RNA motifs in this group are restrictive in their phylogenetic distribution and typically confined to just one to three taxonomic groups. RNA motifs in this group usually have only one or two genes regulated per operon. For novel regulated genes, we focused only on those found in two or more species in a taxonomic group, which increase the likelihood of discovering true regulatory elements.

Examples of RNA motifs from this group include THF, ylbH, and Mg sensor riboswitches (refer to Additional file 3 for the remaining riboswitches). The tetrahydrofolate (THF) riboswitches were restricted to just a few taxa within Firmicutes, with small number of regulated genes for each riboswitch (Additional file 6 and Figure S14; see also RegPrecise). The genes regulated by this riboswitch are most likely responsible for folate uptake (*folT*), with a case of riboswitch regulated folate biosynthesis gene (*folC*) found in *Streptococcus suis*. The *ylbH* riboswitches were only found in Bacillales, with *ylbH* (encoding ribosomal RNA small subunit methyltransferase D (EC 2.1.1.-)) and *coaD* (encoding phosphopantetheine adenylyltransferase (EC 2.7.7.3)) being the only regulated genes (see Additional file 6 and RegPrecise). *coaD* is one component in the pathway involved in the coenzyme A biosynthesis. The magnesium (Mg) sensor riboswitch was only phylogenetically restricted to some species of Enterobacteriales. In all the cases examined, the Mg sensor riboswitch regulates an ATPase component of a P-type transporter, *mgtA*, but other components are absent from the regulon. Some novel regulated genes found in two or more genomes from the group C of riboswitches include *Rv3342* (encoding a methyltransferase) and *draG* (encoding a probable ribosylglycohydrolase), which are regulated by SAM-IV in Mycobacteriaceae.

Group D – ribosomal leaders

There are a variety of ribosomal proteins regulated by conserved leader sequences at the 5' untranslated region of mRNA; they include 5 components (L10, L13, L19, L20, and L21) that form part of the large ribosomal subunit and one component (S15) from the small ribosomal subunit. The regulon content of ribosomal leader sequences are highly conserved and always encode ribosomal operons, with each leader sequence dedicated to a particular ribosomal protein, and each genome contains only a copy of any particular ribosomal operon (Additional file 6 and Figure S15; see also RegPrecise). A Rho-independent transcription terminator forms when excessive cellular ribosomal proteins are detected by the leader sequence, contributing to transcription regulation [8]. The majority of genes regulated under each ribosomal leader are grouped under the SFC of ribosome biogenesis, which is in turn associated with the OFC of protein synthesis (Additional file 5). The number of ribosomal leader regulated genes is fairly small from the species examined, and ribosomal

leaders appear to be confined mostly to Thermotogales and many lineages of Firmicutes, ranging from 6 to more than 10 regulated genes per genome (Additional file 6 and Figure S15). In all, there are 452 ribosomal leader sites regulating 879 genes across 17 taxonomic groups (Additional file 4-6). However, even though all the ribosomal proteins are highly conserved in evolution, their regulatory regions need not be conserved. This is illustrated by the wide variation in the number of regulated genes. However, the structure of operons appeared to be highly conserved between different leader despite the lack of a leader sequence for some operons (see Additional file 1and RegPrecise).

Group E – amino acid leaders

Amino acid leader RNA motifs control genes responsible for biosynthesis and/or transport of amino acids. There are four leader sequences described in Rfam so far with specificities for leucine, histidine, threonine, and tryptophan. The leader sequence achieves regulation by encoding a series of codons specific for the amino acid products of its operon produce. When the cellular level of that particular amino acid drops, ribosome would stall on the leader region of mRNA and results in the formation of an anti-terminator loop that facilitates transcription read-through [9]. Genes regulated by the various amino acid attenuator sequences can all be categorized under the OFCs of amino acid metabolism and amino acid uptake (Additional file 5). Sites for the various amino acid attenuator sequences were found primarily in γ-proteobacteria with few exceptions in other lineages. From our study, we discovered 184 sites across 7 taxonomic groups (Additional file 6 and Figure S16). In γ-proteobacteria, the histidine leader regulons include the histidine biosynthesis enzymes encoded in the *hisGDCBHAFI* operons (see Additional file 1 and RegPrecise). However, in Thermotogales, putative histidine transporters (*hisJML* and *yuiF*) were found under His leader regulaton. For leucine leader, the regulons identified in genomes of Enterobacteriales and Shewanellaceae include the *leuABCD* genes involved in the leucine biosynthesis. For threonine leader, which can be found in genomes of Enterobacteriales and Shewanellaceae, the regulated *thrABC* operons encode enzymes from the threonine biosynthesis pathway. For tryptophan leader, the tryptophan biosynthesis genes *trpEGDCBA* are regulated in Enterobacteriales, Shewanellaceae, and a few genomes of Corynebacteriaceae. However, only *trpB* is regulated in a few genomes of Thermotogales.

Group F – T-boxes

T-boxes are *cis*-regulatory RNA elements that recognize specifically uncharged tRNA molecule and are responsible for regulating genes involved in amino acid metabolism. The majority of SFCs regulated by T-boxes are as follows: (i) amino acid biosynthesis (with 64 unique orthologs), (ii) amino acid transporters (with

29 unique orthologs), and (iii) aminoacyl-tRNA synthetases (with 27 unique orthologs) (Additional file 5). These three SFCs can be classified under the broader OFCs of amino acid metabolism, amino acid uptake, and protein synthesis, respectively. T-boxes are mostly found in Firmicutes, ranging from 26 regulated genes per genome in Clostridiaceae to 32 genes in Lactobacillaceae (Additional file 6 and Figure S17). Altogether we annotated 1134 T-box sites that control 2005 genes in 9 taxonomic groups. On average, these numbers correspond to 1.8 genes per T-box.

The T-boxes are sparsely distributed in Chloroflexi, Deinococcus-Thermus, and both lineages of Actinobacteria examined. In Firmicutes, aminoacyl-tRNA synthetases and amino acid biosynthesis genes appeared to be equally well represented and account for 70% or more of the total regulated genes. In Lactobacillaceae, there is also a large representation of amino acid transporter genes, which makes up around 20% of regulated genes. However, in other taxa examined, aminoacyl-tRNA made up the majority of the regulated genes, ranging from roughly 50 to 100%.

References

1. Singh P, Sengupta S: **Phylogenetic analysis and comparative genomics of purine riboswitch distribution in prokaryotes.** *Evol Bioinform Online* 2012, 8:589-609.
2. Zhang Y, Gladyshev VN: **Molybdoproteomes and evolution of molybdenum utilization.** *J Mol Biol* 2008, 379:881-99.
3. Reader JS, Metzgar D, Schimmel P, de Crécy-Lagard V: **Identification of four genes necessary for biosynthesis of the modified nucleoside queuosine.** *J Biol Chem* 2004, 279:6280-5.
4. Eitinger T, Rodionov DA, Grote M, Schneider E: **Canonical and ECF-type ATP-binding cassette importers in prokaryotes: diversity in modular organization and cellular functions.** *FEMS Microbiol Rev* 2011, 35:3-67.
5. Barrick JE, Corbino KA, Winkler WC, Nahvi A, Mandal M, Collins J, Lee M, Roth A, Sudarsan N, Jona I, Wickiser JK, Breaker RR: **New RNA motifs suggest an expanded scope for riboswitches in bacterial genetic control.** *Proc Natl Acad Sci U S A* 2004, 101:6421-6.
6. Weinberg Z, Barrick JE, Yao Z, Roth A, Kim JN, Gore J, Wang JX, Lee ER, Block KF, Sudarsan N, Neph S, Tompa M, Ruzzo WL, Breaker RR: **Identification of 22 candidate structured RNAs in bacteria using the CMfinder comparative genomics pipeline.** *Nucleic Acids Res* 2007, 35:4809-19.
7. Winkler WC, Nahvi A, Roth A, Collins JA, Breaker RR: **Control of gene expression by a natural metabolite-responsive ribozyme.** *Nature* 2004, 428:281-6.
8. Choonee N, Even S, Zig L, Putzer H: **Ribosomal protein L20 controls expression of the Bacillus subtilis infC operon via a transcription attenuation mechanism.** *Nucleic Acids Res* 2007, 35:1578-88.
9. Kolter R, Yanofsky C: **Attenuation in amino acid biosynthetic operons.** *Annu Rev Genet* 1982, 16:113-34.
10. Watson PY, Fedor MJ. **The ydaO motif is an ATP-sensing riboswitch in Bacillus subtilis.** *Nat Chem Biol.* 2012, 8:963-5.
11. Saier MH Jr, Yen MR, Noto K, Tamang DG, Elkan C. **The Transporter Classification Database: recent advances.** *Nucleic Acids Res*. 2009, 37:D274-8.
