# Supplementary material for: Comparative genomics of metabolic capacities of regulons controlled by cis-regulatory RNA motifs in bacteria
Source: BMC Genomics. 2013 Sep 2;14:597. doi: 10.1186/1471-2164-14-597 (PMC3766115; doi:10.1186/1471-2164-14-597)
Supplement: Additional file 8 — Supporting figures S1-17 for Additional file 7 (Comparative genomics analysis of functional regulon content for RNA motifs from groups B, C, D, E, F). Each figure represents functional composition of RNA motif regulons across different lineages. Average number of RNA-regulated genes per genome denotes the overall height of each bar. Colored bar parts show functional regulon composition using SFC categories indicated in the legend. For each taxonomic group, a number in parenthesis represents the number of studied genomes. Phylum / subdivision names are indicated by braces beneath the taxon names. [file 1471-2164-14-597-S8.pdf]

Additional file 8 – Supporting figures S1-17 for Additional file 7 (Comparative genomics analysis of functional regulon content for RNA motifs from groups B, C, D, E, F).

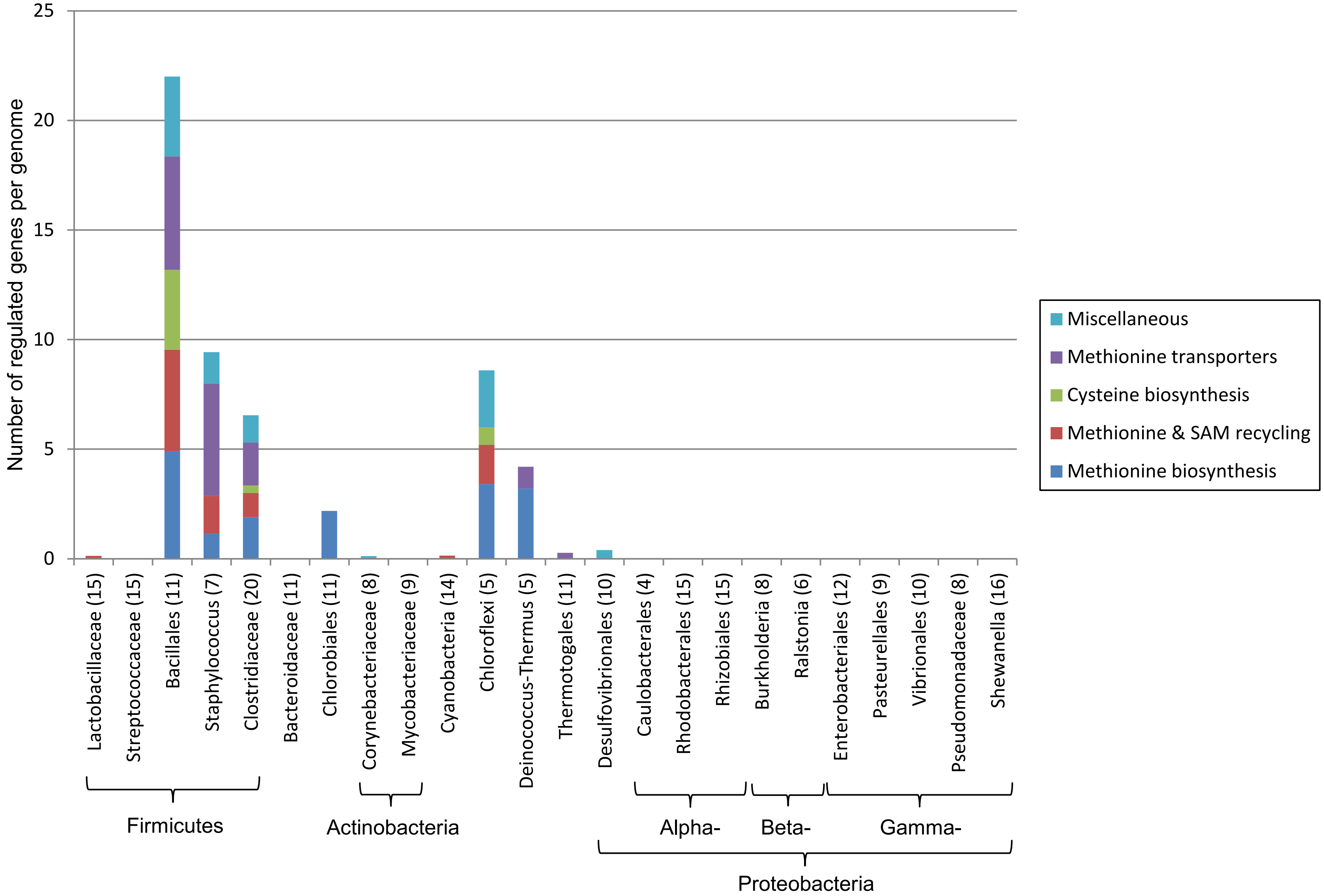

Figure S1. **Functional composition of SAM riboswitch regulons across different lineages.**
Average number of riboswitch regulated genes per genome denotes the overall height of each bar. Colored bar parts show functional regulon composition using SFC categories indicated in the legend. For each taxonomic group, a number in parenthesis represents the number of studied genomes. Phylum / subdivision names are indicated by braces beneath the taxon names.

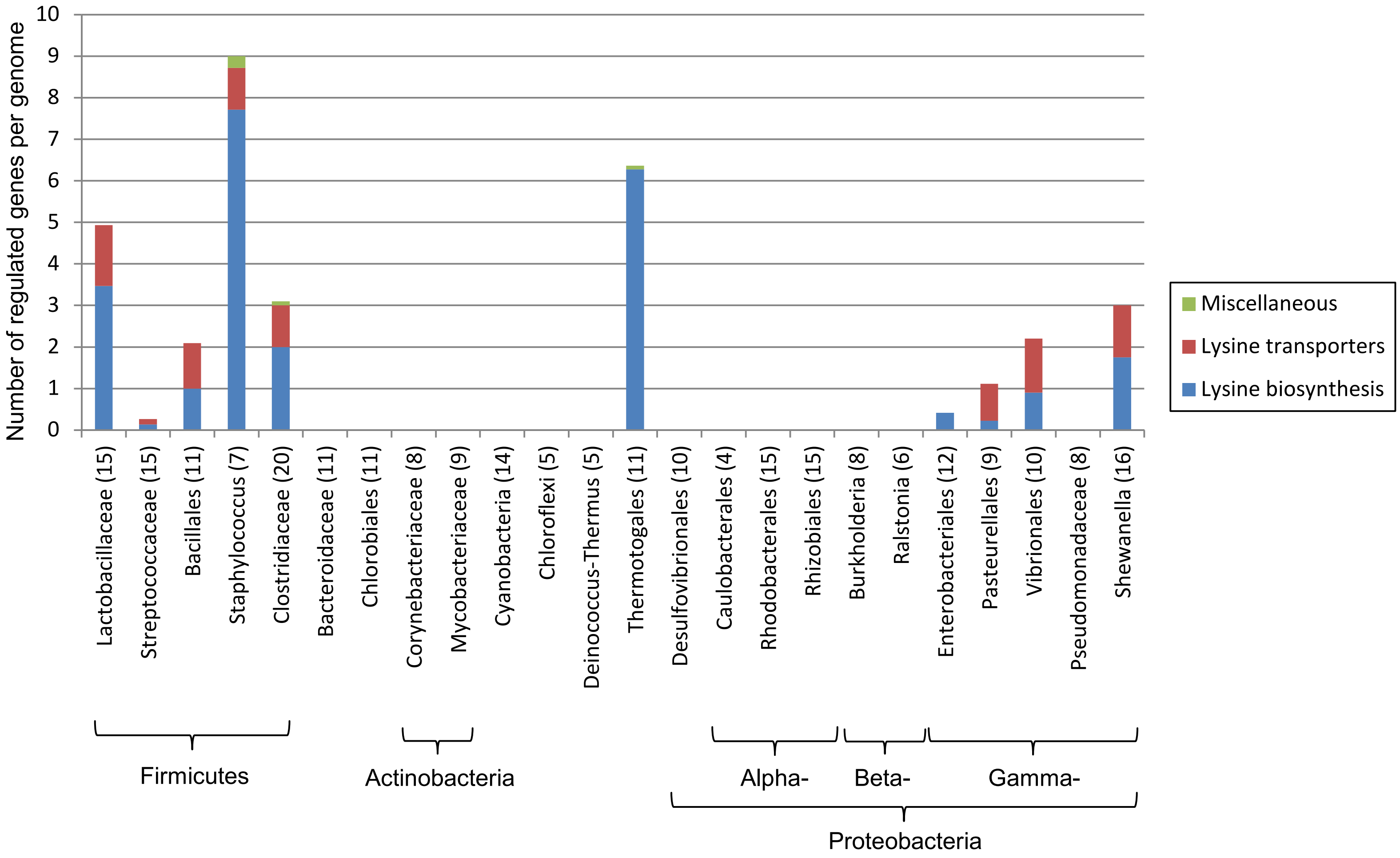

Figure S2. **Functional composition of lysine riboswitch regulons across different lineages.**
See Figure S1 for figure descriptions.

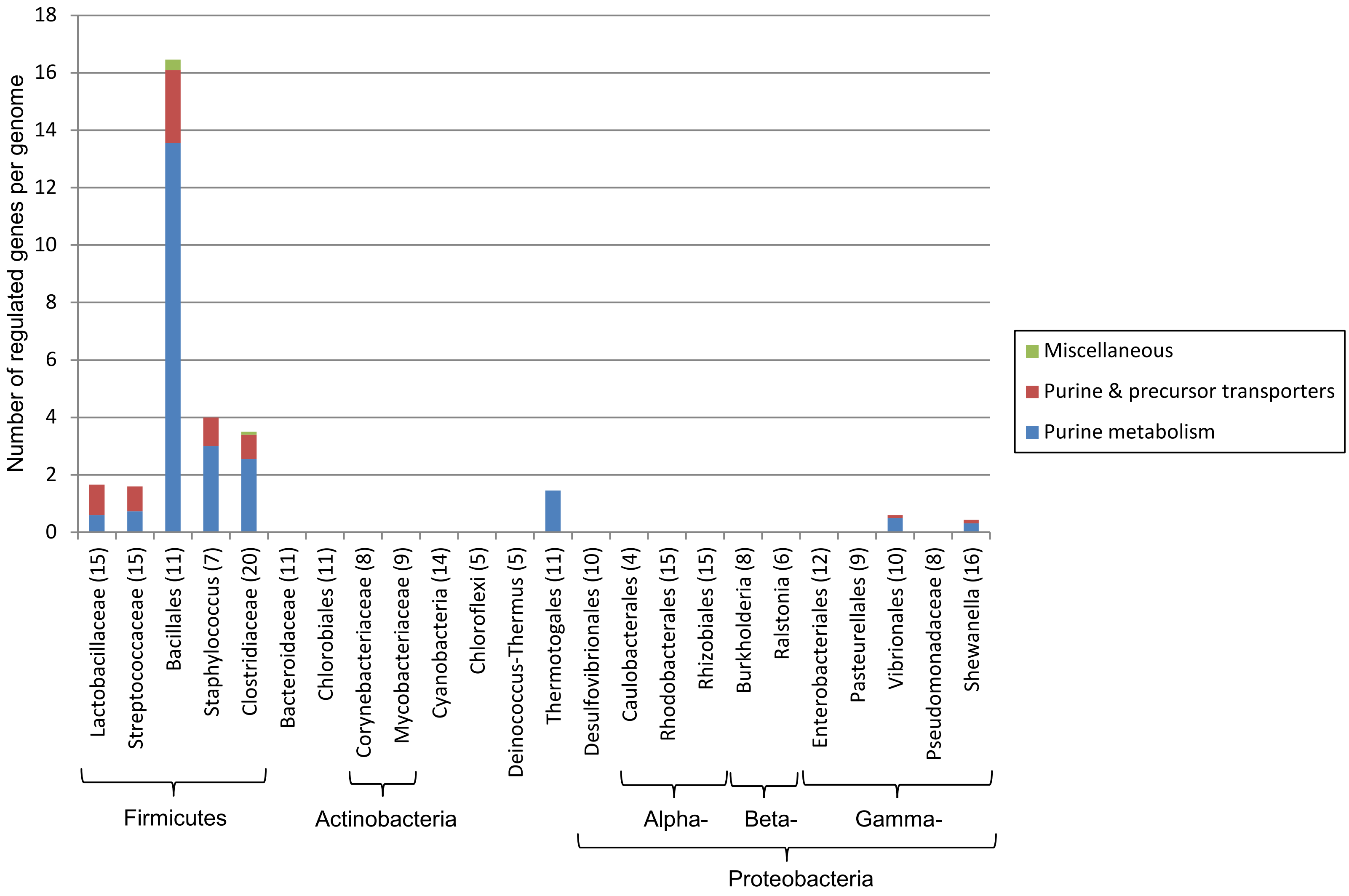

Figure S3. **Functional composition of purine riboswitch regulons across different lineages.** See Figure S1 for figure descriptions.

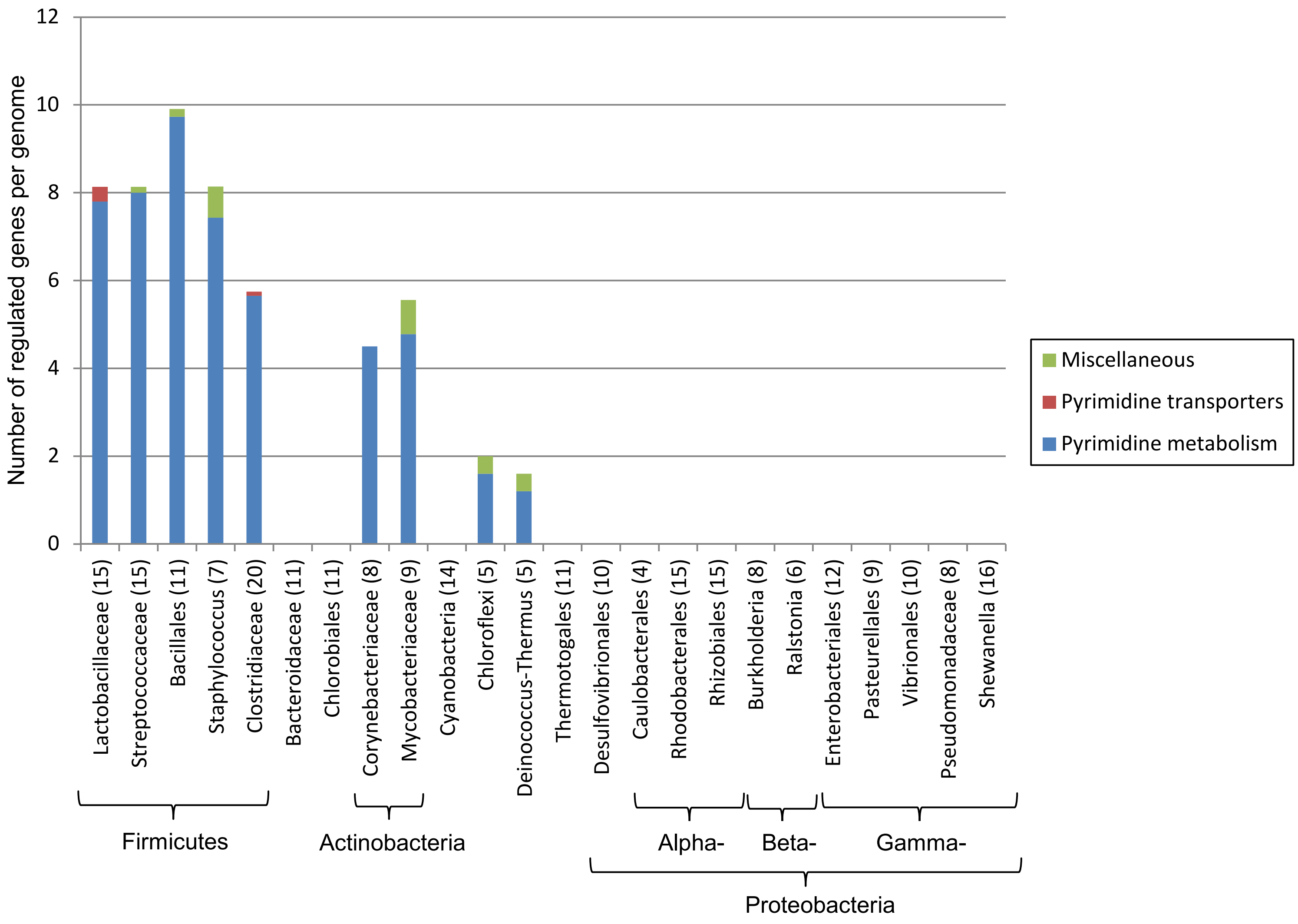

Figure S4. **Functional composition of PyrR binding RNA motif regulons across different lineages.** See Figure S1 for figure descriptions.

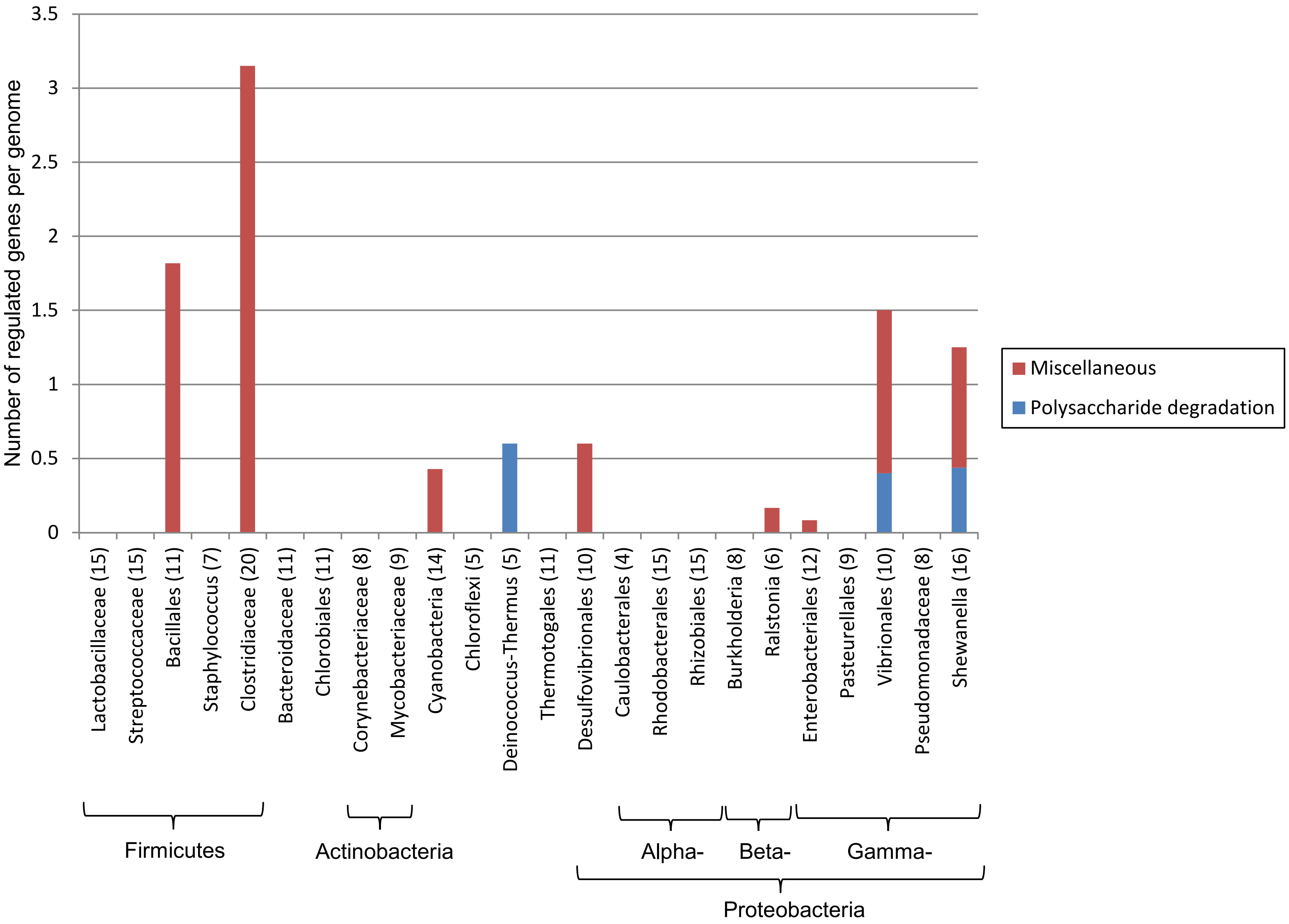

Figure S5. **Functional composition of GEMM riboswitch regulons across different lineages.** See Figure S1 for figure descriptions.

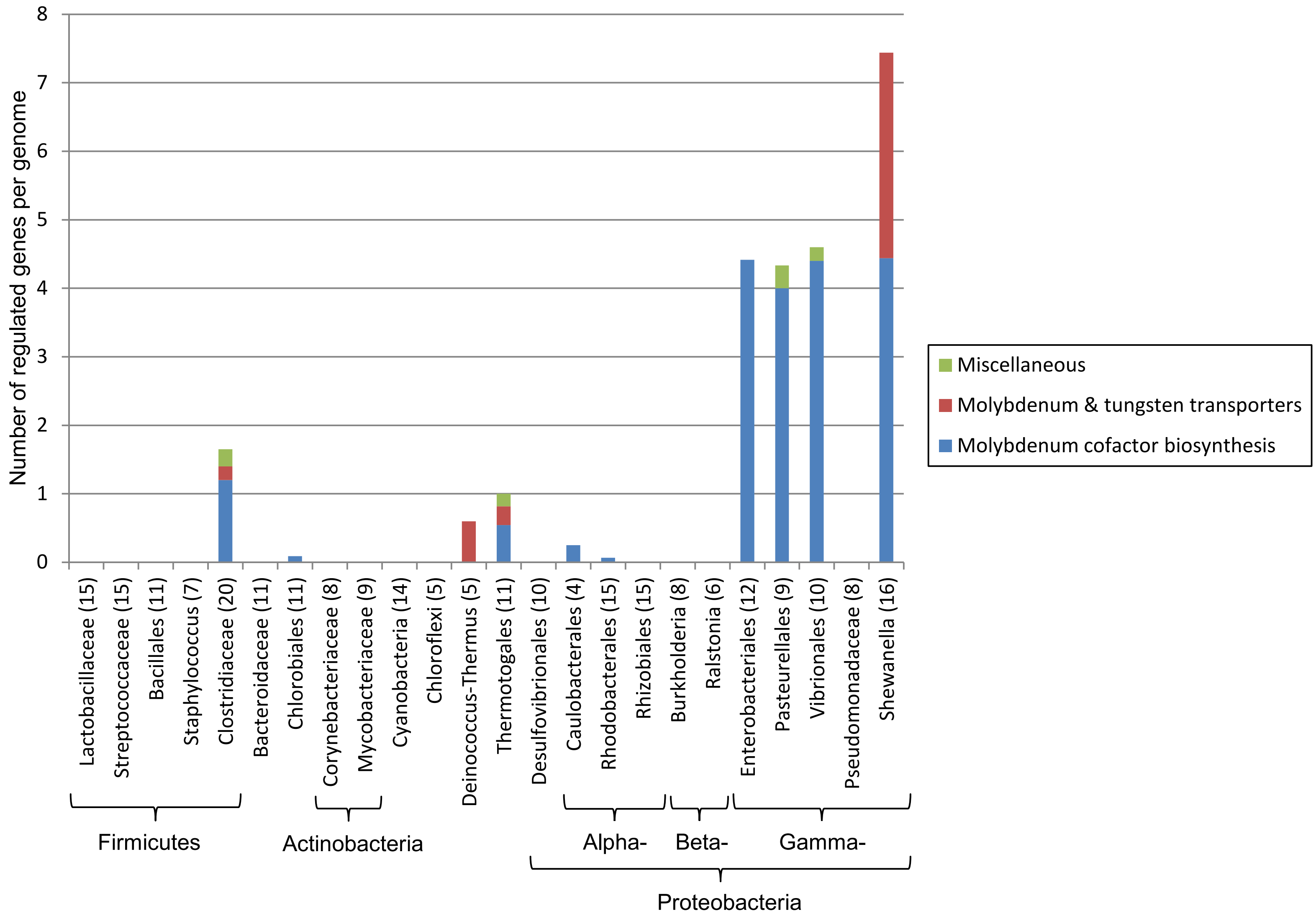

Figure S6. **Functional composition of MOCO RNA motif regulons across different lineages.** See Figure S1 for figure descriptions.

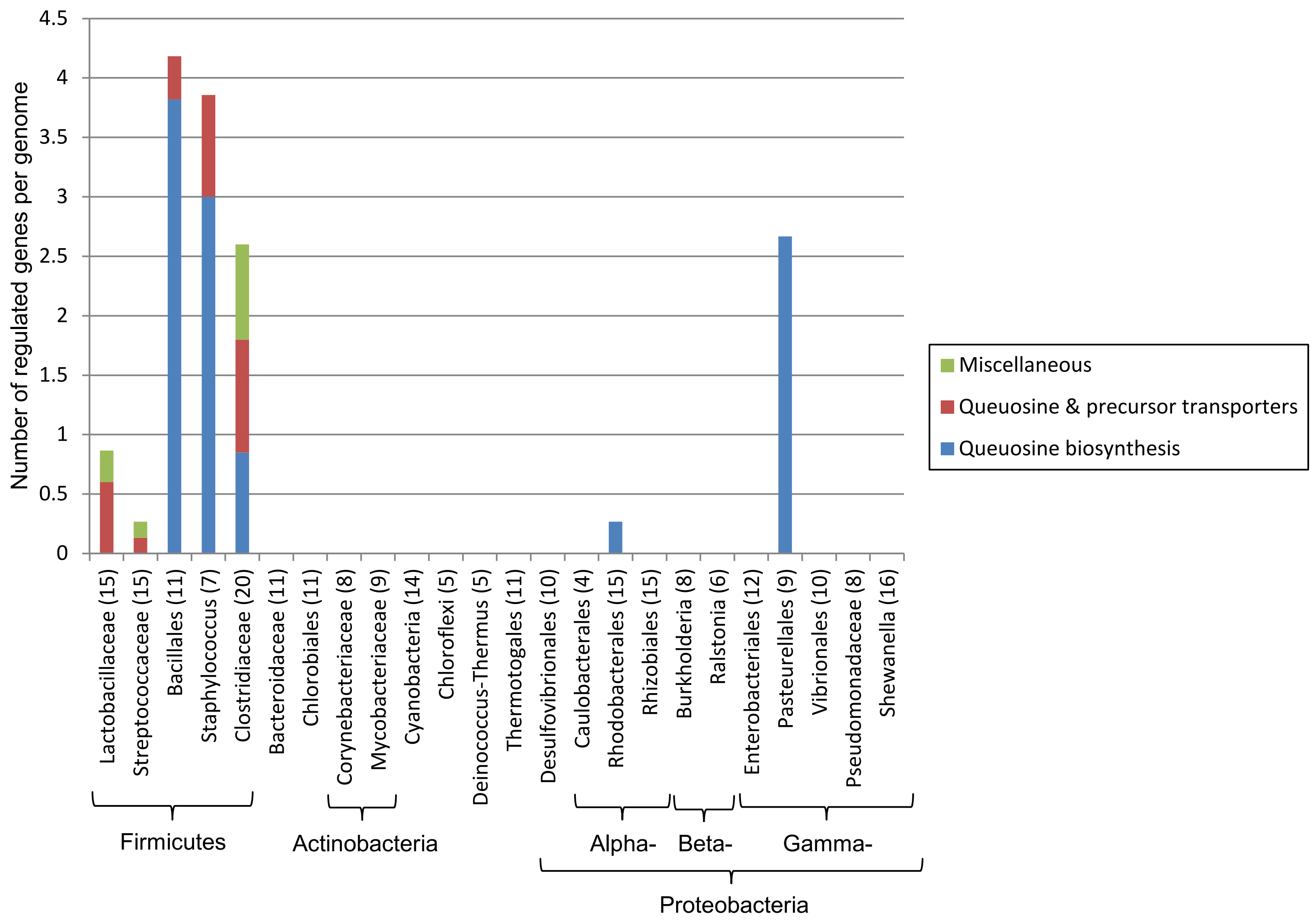

Figure S7. **Functional composition of PreQ1 riboswitch regulons across different lineages.** See Figure S1 for figure descriptions.

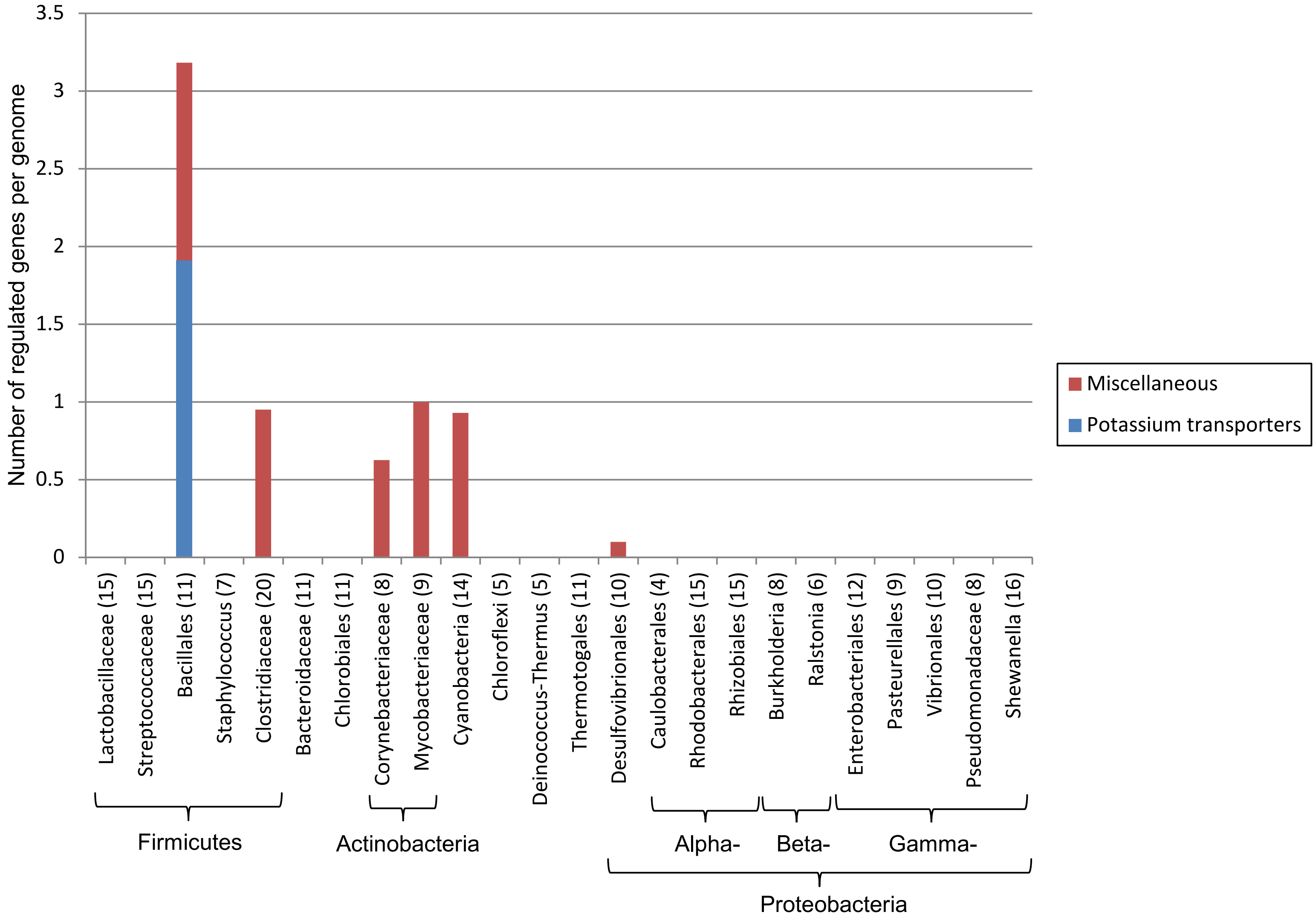

Figure S8. **Functional composition of ydaO-yuaA riboswitch regulons across different lineages.** See Figure S1 for figure descriptions.

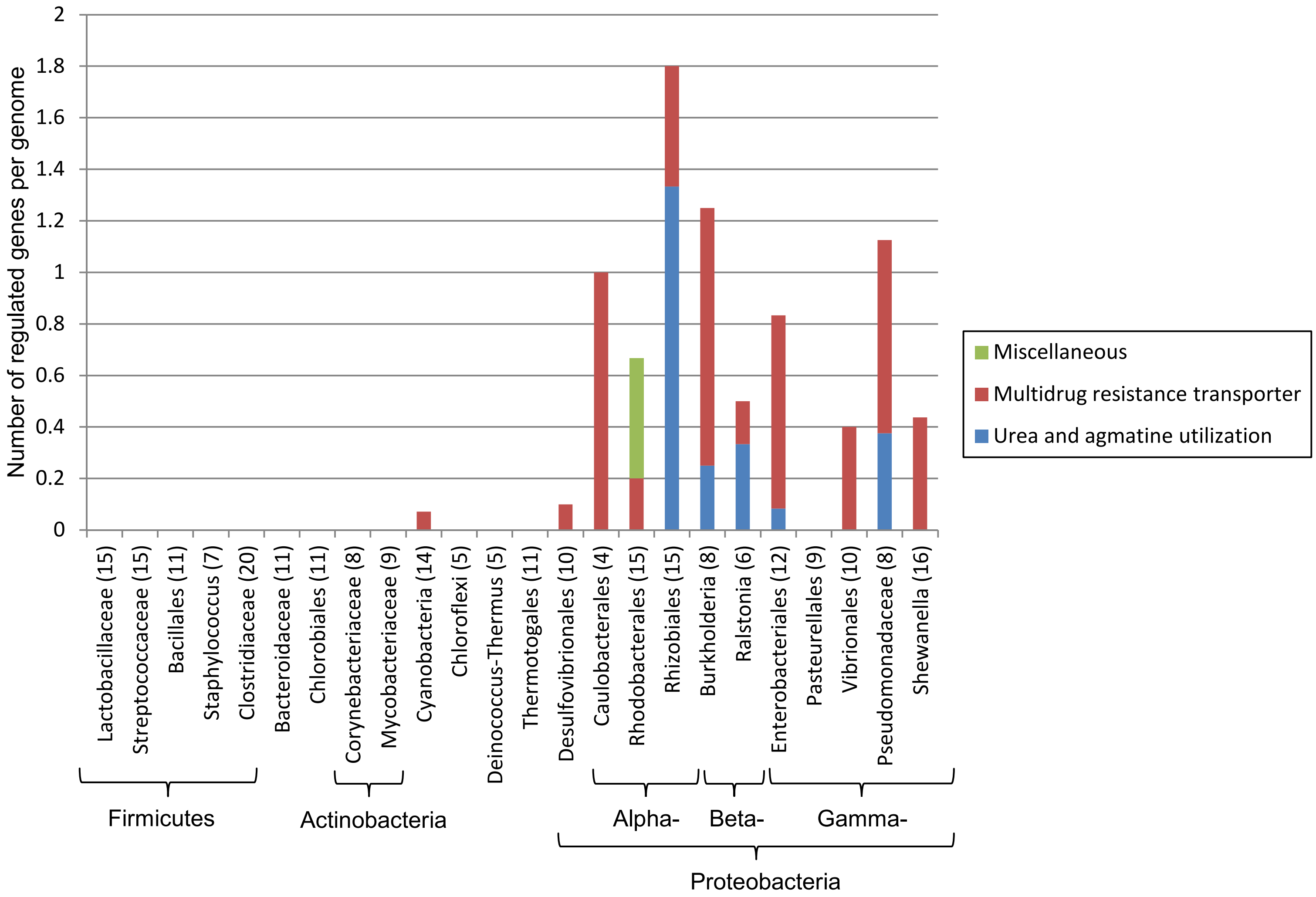

Figure S9. **Functional composition of mini-ykkC RNA motif regulons across different lineages.** See Figure S1 for figure descriptions.

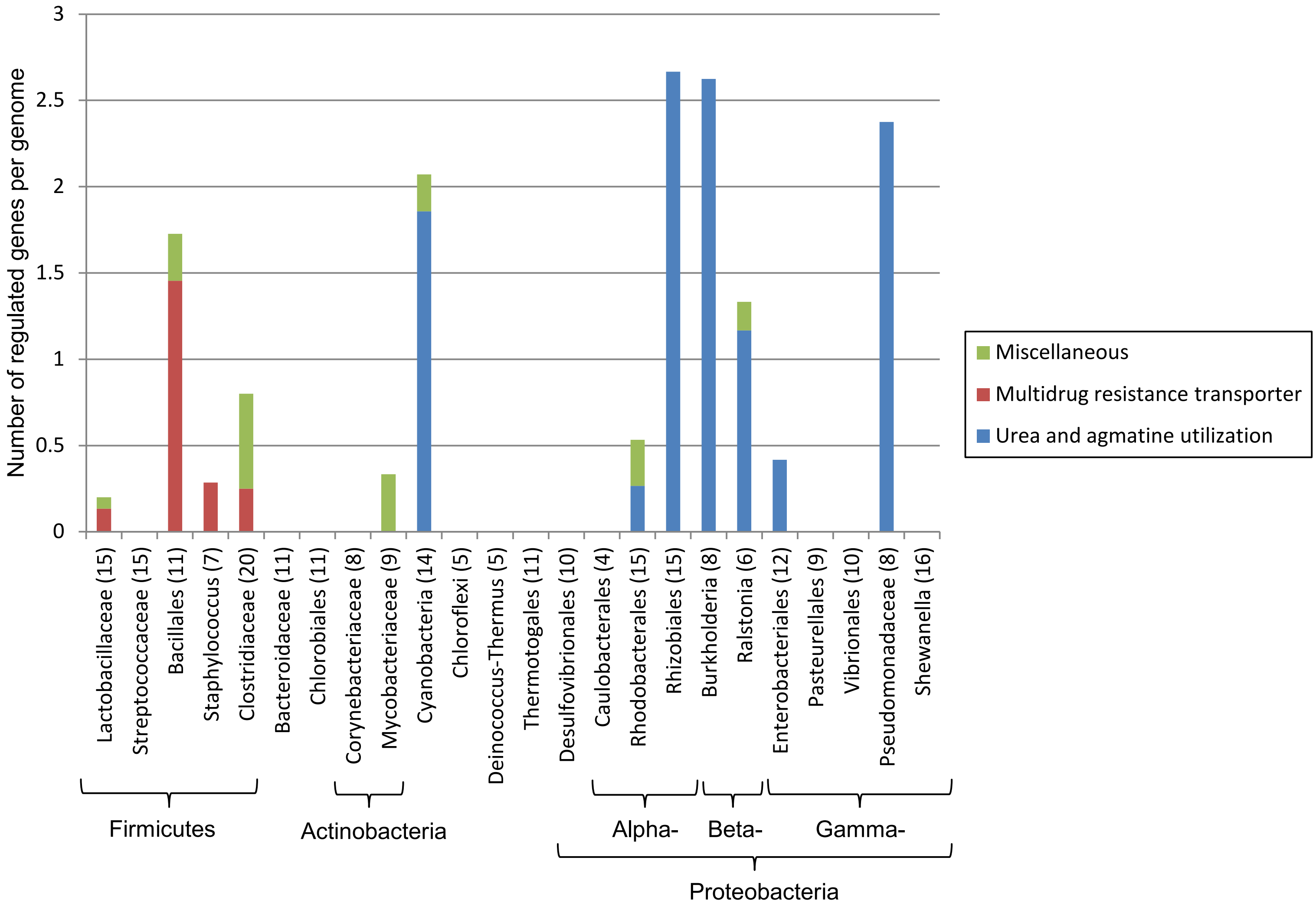

Figure S10. **Functional composition of ykkC-yxkD RNA motif regulons across different lineages.**
See Figure S1 for figure descriptions.

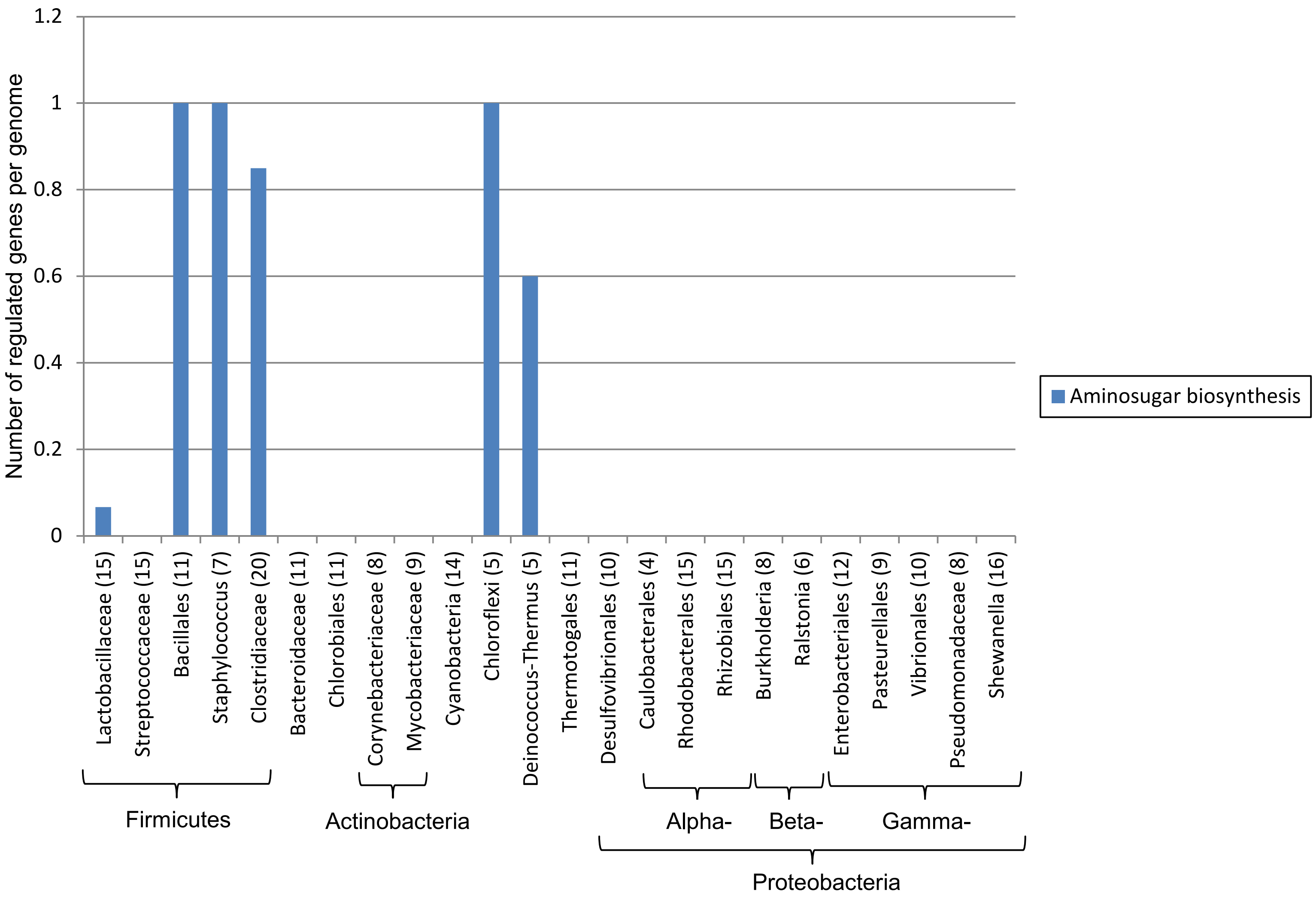

Figure S11. **Functional composition of glmS riboswitch regulons across different lineages.**
See Figure S1 for figure descriptions.

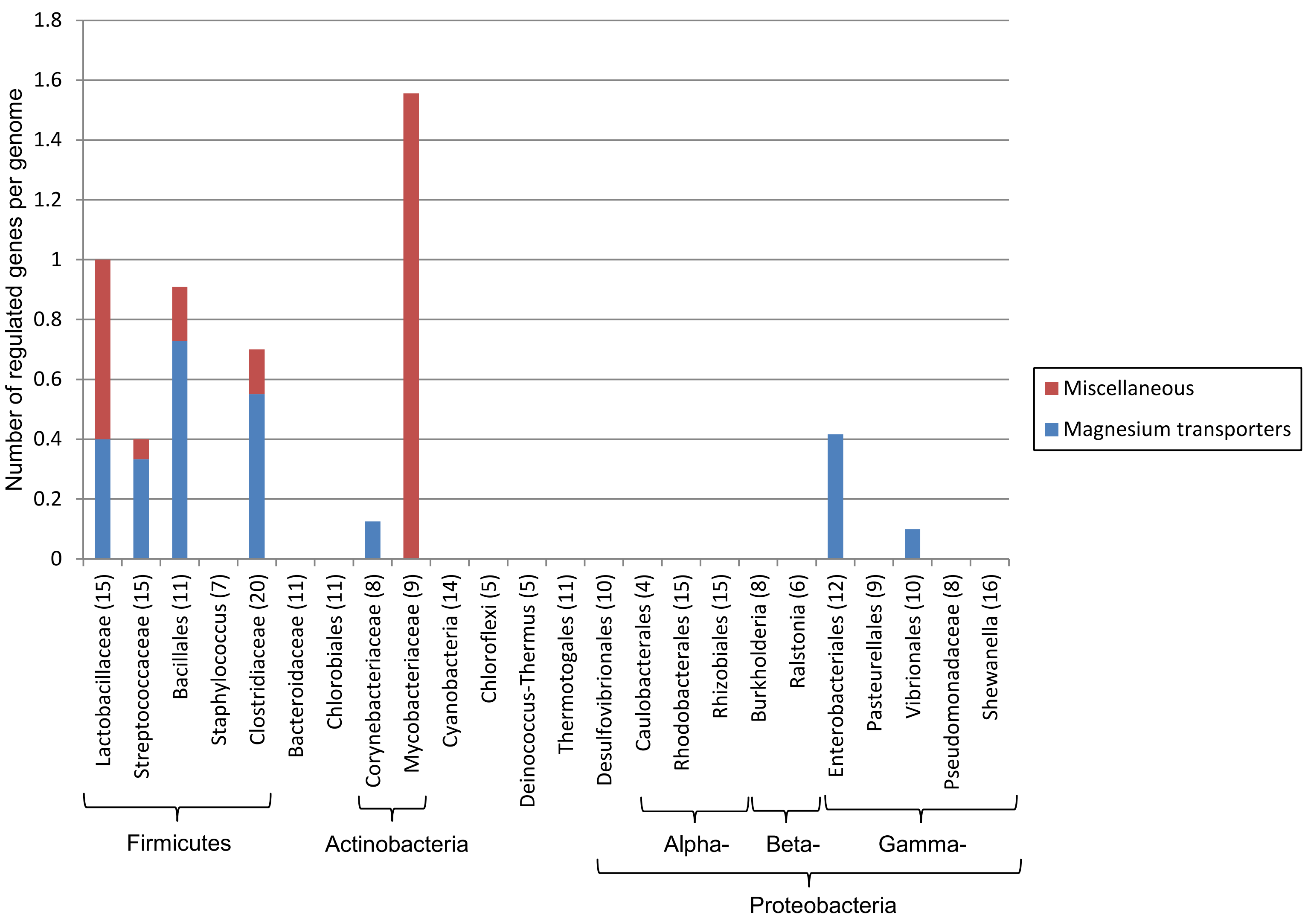

Figure S12. **Functional composition of ykoK RNA motif regulons across different lineages.**
See Figure S1 for figure descriptions.

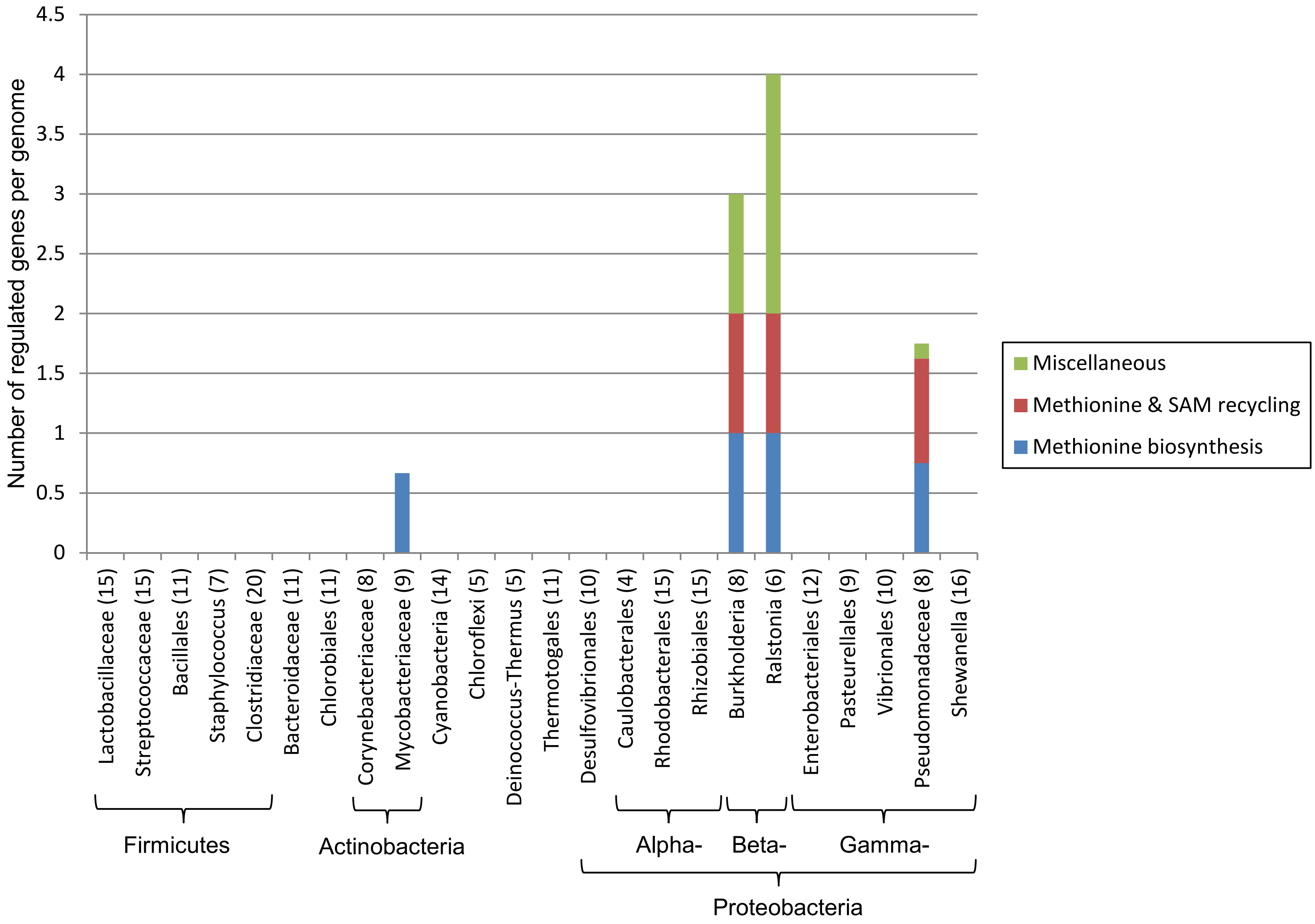

Figure S13. **Functional composition of SAH riboswitch regulons across different lineages.** See Figure S1 for figure descriptions.

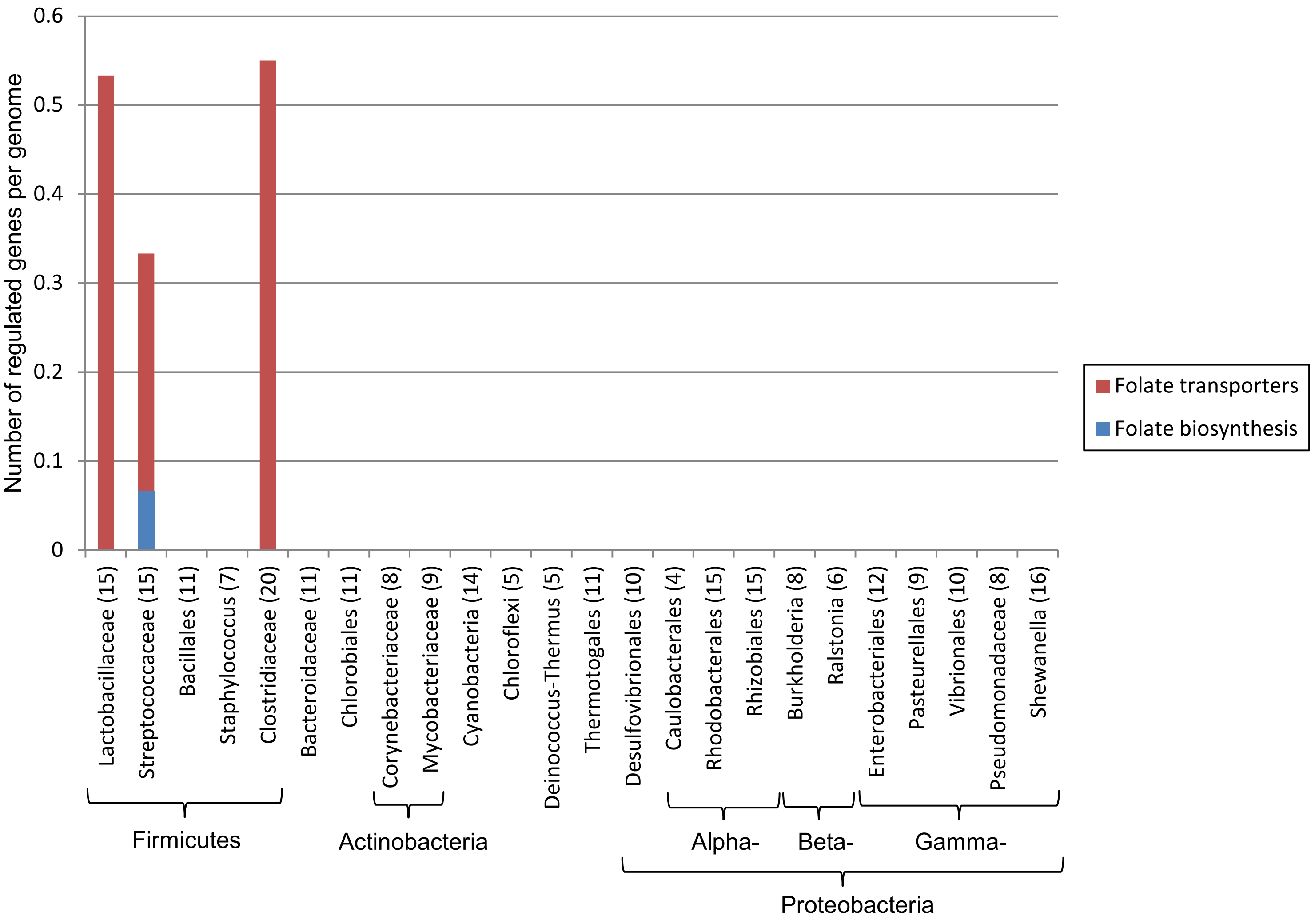

Figure S14. **Functional composition of THF riboswitch regulons across different lineages.**
See Figure S1 for figure descriptions.

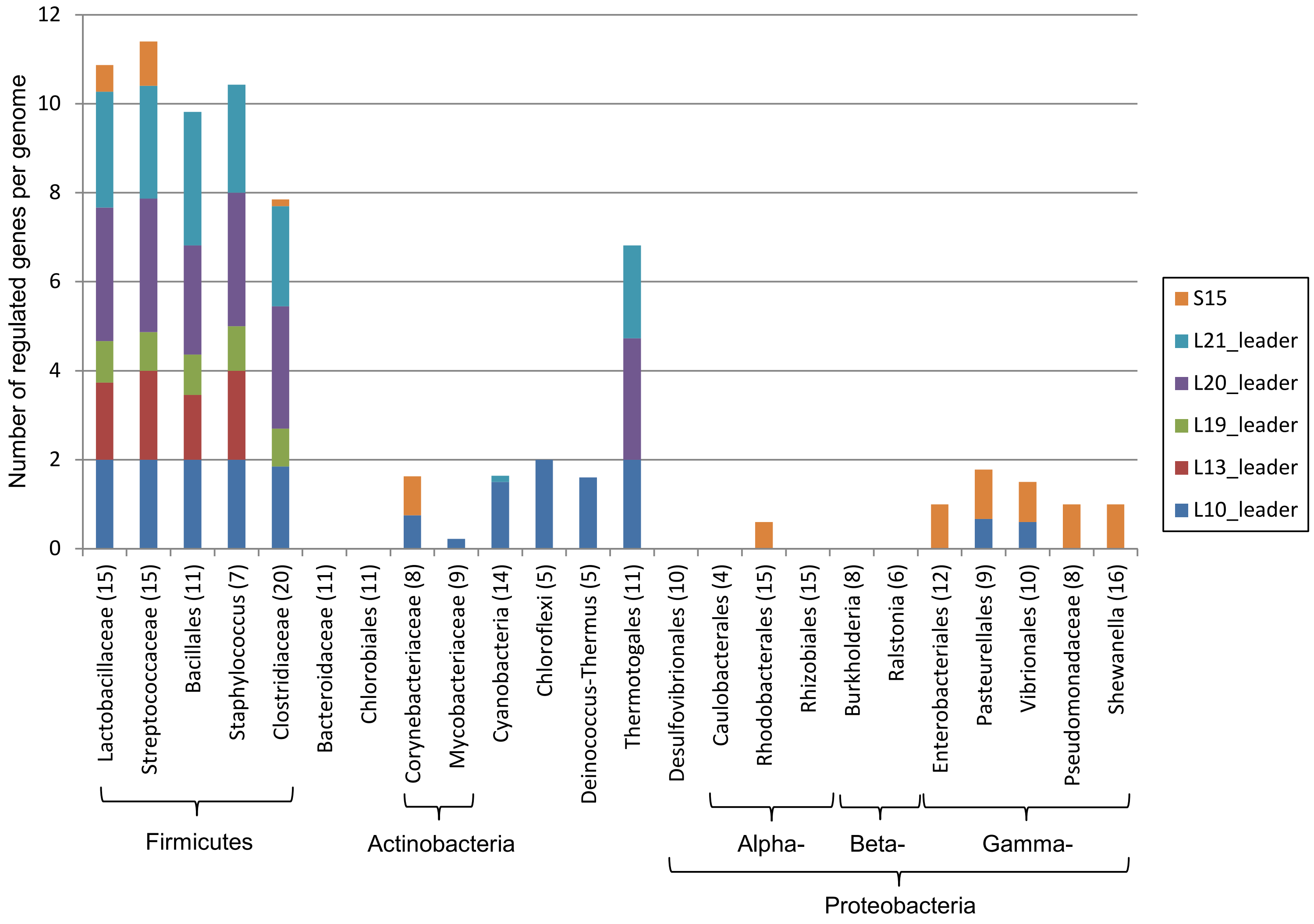

Figure S15. **Distribution of genes controlled by ribosomal leader regulons across different lineages.**
See Figure S1 for figure descriptions. Note that the colored bar parts show the combined gene number under each leader sequence (see legend) instead of individual SFC categories.

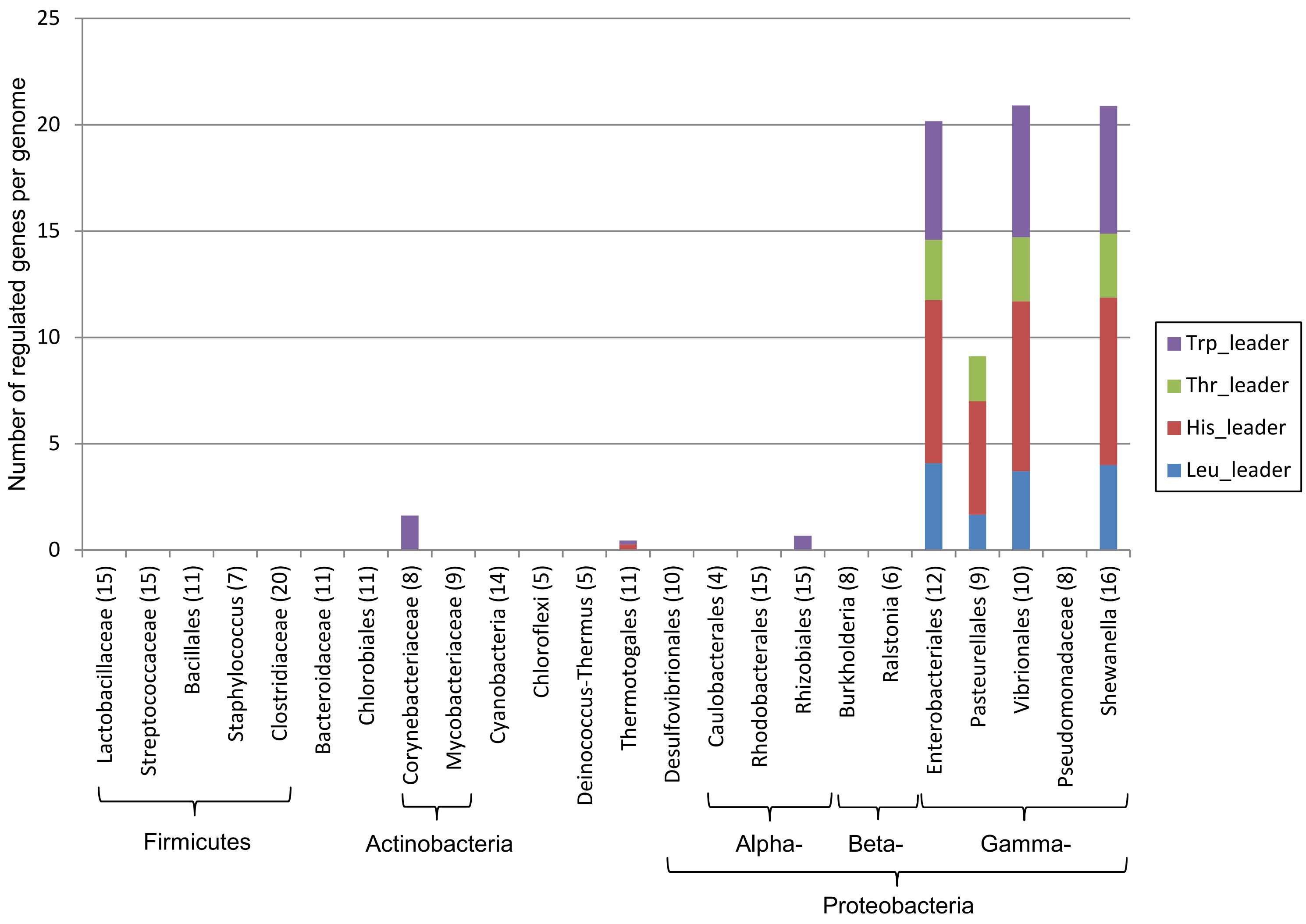

Figure S16. **Distribution of genes controlled by amino acid leader regulons across different lineages.**
See Figure S1 for figure descriptions. Note that the colored bar parts show the combined gene number under each leader sequence (see legend) instead of individual SFC categories.

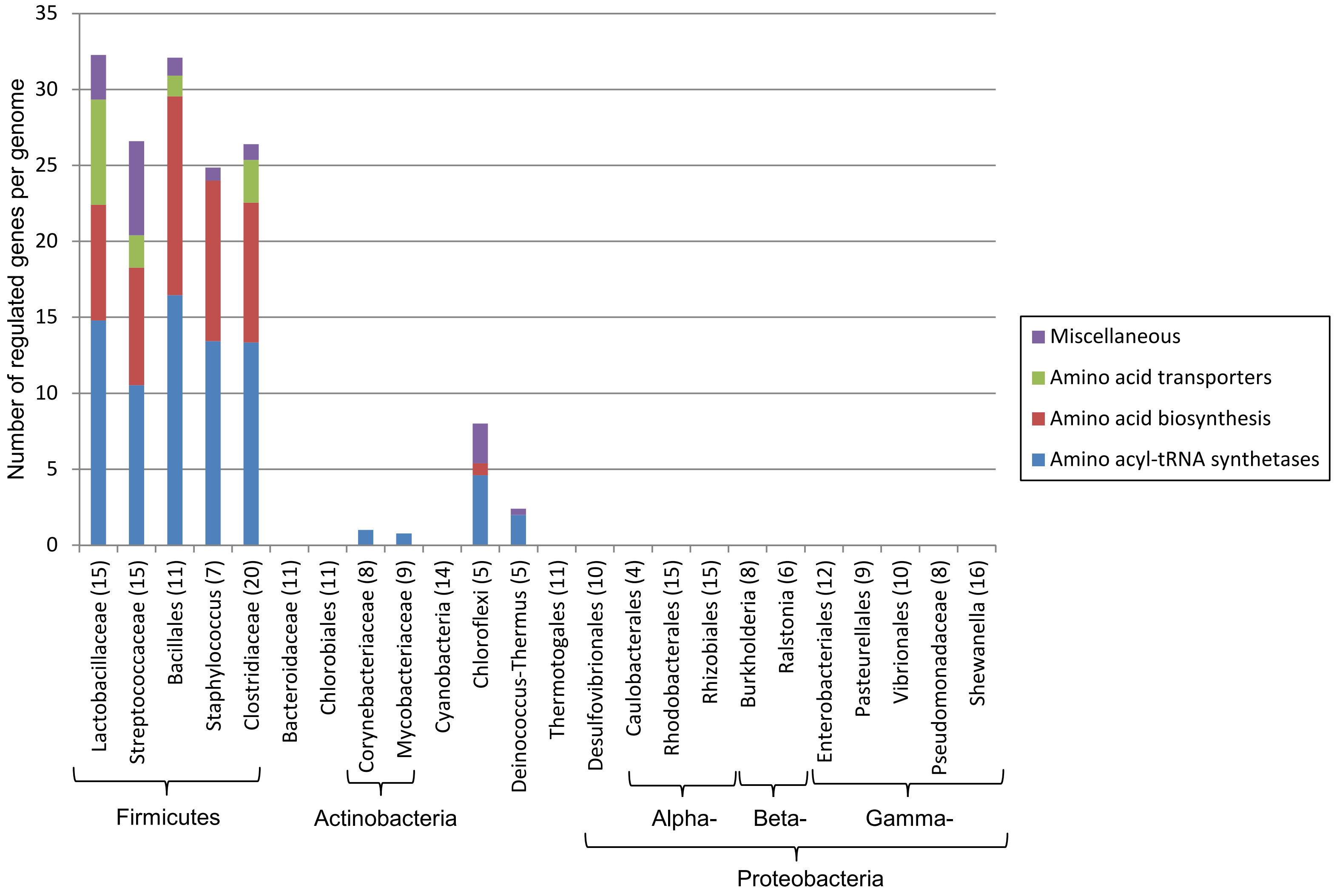

Figure S17. **Functional composition of T-box regulons across different lineages.**
See Figure S1 for figure descriptions.
