# Supplementary material for: Comparative genomics of metabolic capacities of regulons controlled by cis-regulatory RNA motifs in bacteria
Source: BMC Genomics. 2013 Sep 2;14:597. doi: 10.1186/1471-2164-14-597 (PMC3766115; doi:10.1186/1471-2164-14-597)
Supplement: Additional file 5 — Classification of identified RNA motif-regulated genes by specific and overall functional categories (SFC and OFC). Only genes with annotated functional roles in the RegPrecise database are shown under ‘gene name’; number of gene families indicates the total types of different orthologues found. [file 1471-2164-14-597-S5.pdf]

Additional file 5. Classification of RNA motif-regulated genes by specific and overall functional categories (SFC and OFC).

Riboswitch	SFC category	Number of gene families and their names*		OFC category
Cobalamin	B12 & precursor transporters	26	btuB, btuF, btuC, btuD, btuT, btuM, btuU, btuV, btuN, btuX, btuY, cblT, cbrV, cbrU, cbrT, RoseRS_3102, RoseRS_3101, RoseRS_3100, Pjdr2_2686, Pjdr2_2687, Pjdr2_2688, btuB2-btuM-btuR, btuD-cbiZ	2. Coenzyme uptake
Cobalamin	B12 biosynthesis	43	cobD, cobC, cobU, cobS, cobT, btuR, pduO, cbiP, cbiB, cblS, pduX, cobG, cobE, cobF, cysG, cbiL, cbiH, cbiG, cbiF, cbiD, cbiJ, cbiE, cbiT, cbiC, cbiA, cbiX, cbiK, cobN, chlI, chlD, cbiET, cbiGH, cbiGF. cbiHC. cbiHJ. cbiJD. cbiKL. cbiOP. cbiP-cobU. cbiXC. chlID.	1. Coenzyme metabolism
Cobalamin	Cobalt transporters	12	cbtA, cbtB, cbtC, cbtD, cbtG, hoxN, cbiM, cbiN, cbiQ, cbiO, hupE, Ssed_2094	6. Metal homeostasis
Cobalamin	Isozymes of B12-dependent enzymes	13	nrdA, nrdB, nrdD, nrdG, nrdE, nrdF, nrdH, nrdI, metE, metF, ardX, frdX, achX	10. Miscellaneous
FMN	Riboflavin biosynthesis	7	ribD, ribE, ribB, ribA, ribH, ribBA, ribBH	1. Coenzyme metabolism
FMN	Riboflavin transporters	5	ribU, impX, pnuX, ribX, ribY	2. Coenzyme uptake
GEMM	Polysaccharide degradation	4	chiA, cbp, Deide_1p01370, licB	10. Miscellaneous
glmS	Aminosugar biosynthesis	1	glmS	5. Secondary metabolism
Glycine	Glycine metabolism	12	grdB, grdE, grdX, grdA, grdC, grdD, glyA, abgB, gcvT, gcvH, gcvP, gcvB	3. Amino acid metabolism
Glycine	Glycine transporters	3	glyP, ydgF, potE	4. Amino acid uptake
Glycine	Serine metabolism	3	sdaA, serC, serA	3. Amino acid metabolism
His leader	Histidine biosynthesis	8	hisG, hisD, hisC, hisB, hisH, hisA, hisF, hisI	3. Amino acid metabolism
His leader	Histidine transporters	4	hisJ, hisM, hisL, yuiF	4. Amino acid uptake
L10 leader	Ribosome biogenesis	2	rplJ, rplL	9. Protein synthesis
L13 leader	Ribosome biogenesis	2	rplM, rpsI	9. Protein synthesis
L19 leader	Ribosome biogenesis	1	rplS	9. Protein synthesis
L20 leader	Ribosome biogenesis	3	infC, rpmI, rplT	9. Protein synthesis
L21 leader	Ribosome biogenesis	4	rplU, rpmA, rplM, rpsI	9. Protein synthesis
Leu leader	Leucine biosynthesis	4	leuA, leuB, leuC, leuD	3. Amino acid metabolism
Lysine	Lysine biosynthesis	11	lysC, asd, dapF, dapA, dapB, dapD, dapL, dapX, lysA, argD, patA	3. Amino acid metabolism
Lysine	Lysine transporters	8	lysW, lysP, yvsH, lysX, lysY, Clos_0722, Clos_0723, Clos_0724	4. Amino acid uptake
Mg sensor	Magnesium transporters	1	mgtA	6. Metal homeostasis
mini-ykkC	Multidrug resistance transporter	1	ykkC	10. Miscellaneous
mini-ykkC	Urea and agmatine utilization	3	ucaA, ucaB, amt	5. Secondary metabolism
MOCO	Molybdenum & tungsten transporters	6	modA, modB, modC, tupA, tupB, tupC	6. Metal homeostasis
MOCO	Molybdenum cofactor biosynthesis	8	moaA, moaB, moaC, moaD, moaE, mobA, mobB, moeA	1. Coenzyme metabolism
preQ1	Queuosine & precursor transporters	3	ypdP, queT, qrtT	2. Coenzyme uptake
preQ1	Queuosine biosynthesis	4	queC, queD, queE, queF	1. Coenzyme metabolism
preQ1-II	Queuosine & precursor transporters	1	queT	2. Coenzyme uptake
Purine	Purine & precursor transporters	6	pbuG, pbuO, pbuX, nupG, pbuE	8. Nucleotide uptake
Purine	Purine metabolism	18	guaB, guaA, guaD, purE, purK, purB, purC, purS, purQ, purL, purF, purM, purN, purH, purD, adeC, add, xpt	7. Nucleotide metabolism
PyrR	Pyrimidine metabolism	14	pyrR, pyrP, pyrB, pyrC, carA, carB, pyrK, pyrD, pyrF, pyrE, pyrG, pyrI, ntd, codA	7. Nucleotide metabolism
PyrR	Pyrimidine transporters	1	codB	8. Nucleotide uptake
S15 leader	Ribosome biogenesis	1	rpsO	9. Protein synthesis
SAH	Methionine & SAM recycling	1	ahcY	3. Amino acid metabolism
SAH	Methionine biosynthesis	2	metF, metH	3. Amino acid metabolism
SAM	Cysteine biosynthesis	13	cysH, sat, cysC, cysI, sirB, sirC, cysG, cysK, cysP, sbpA, cysT, cysW, cysA	3. Amino acid metabolism
SAM	Methionine & SAM recycling	11	metK, mtnK, mtnA, mtnW, mtnX, mtnB, mtnD, rhc, ahcY, mmuP, mtnE	3. Amino acid metabolism
SAM	Methionine biosynthesis	11	metX, metY, metA, metE, metF, metH, metI, metC, hom, bhmT, msd	3. Amino acid metabolism
SAM	Methionine transporters	4	metN, metP, metQ, metT	4. Amino acid uptake
SAM-Alpha	Methionine biosynthesis	6	metY, metX, metW, metA, hom, metC	3. Amino acid metabolism
SAM-Chlorobi	Methionine & SAM recycling	1	metK	3. Amino acid metabolism
SAM-Chlorobi	Methionine & SAM recycling	1	ahcY	3. Amino acid metabolism
SAM-IV	Methionine biosynthesis	2	metY, metX	3. Amino acid metabolism
SAM-SAH	Methionine & SAM recycling	1	metK	3. Amino acid metabolism
serC	Serine metabolism	2	serC, serA	3. Amino acid metabolism
SMK_box	Methionine & SAM recycling	1	metK	3. Amino acid metabolism
speF	Ornithine degradation	1	speF	3. Amino acid metabolism
sucA	Citric acid cycle	3	sucA, sucB, lpdA	5. Secondary metabolism

T-box	Amino acid biosynthesis	**64**	argG, argF, argC, argJ, argB, argD, argH, asnA, asnB, cysE, cysK, mccB, mccA, hisC, hisZ, hisG, hisD, hisB, hisH, hisA, hisF, hisI, hisE, hisK, ilvE, ilvB, ilvH, ilvC, ilvD, ilvA, leuA, leuB, leuC, leuD, asd, metA, metB, metY, hom, metI, metC, metF, metE, yxjH, mmuM, proB, proA, proI, serA, yurG, thrC, thrB, trpE, trpG, trpD, trpC, trpF, trpB, trpA, tyrA, aroA, aroF, aroE, phhA	3. Amino acid metabolism
T-box	Amino acid transporters	**29**	artP, artQ, artM, glnQ, glnH, glnM, glnP, tcyA, tcyB, tcyC, cysP, hisZ, hisX, hisY, brnQ, yvbW, yocR, lysX, metQ, metN, metP, ansP, ykbA, yhdH, trpX, trpY, trpZ, trpP, tyrT	4. Amino acid uptake
T-box	Amino acyl-tRNA synthetases	**27**	alaS, asnS, aspS, gatC, gatA, gatB, argS, hisS, cysS, glyQ, glyS, ileS, leuS, lysS, lysU, glnS, metS, pheS, pheT, proS, serS, thrS, thrZ, trpS, tyrS, tyrZ, valS	9. Protein synthesis
THF	Folate biosynthesis	**1**	folC	1. Coenzyme metabolism
THF	Folate transporters	**1**	folT	2. Coenzyme uptake
Thr leader	Threonine biosynthesis	**3**	thrA, thrB, thrC	3. Amino acid metabolism
TPP	Thiamine & precursor transporters	**19**	thiX, thiY, thiZ, ykoF, ykoE, ykoD, ykoC, cytX, thiU, thiW, ecfA, ecfT, thiT, pnuT, thiB, thiP, thiQ, omp_thi, thiW-ecfAA	2. Coenzyme uptake
TPP	Thiamine biosynthesis	**20**	tenA, tenI, thiC, thiD, thiE, thiS, thiF, thiG, thiH, thiO, thiM, thiN, thi4, tnr3, thiK, thiDE, thiDN, thiD-tenA, thiED-tenA, thiOS	1. Coenzyme metabolism
Trp leader	Tryptophan biosynthesis	**7**	aroA, trpE, trpG, trpD, trpC, trpB, trpA	3. Amino acid metabolism
ybhL	Miscellaneous	**1**	ybhL	10. Miscellaneous
ydaO-yuaA	Potassium transporters	**6**	ktrA, ktrB, kdpA, kdpB, kdpC, kdpD	6. Metal homeostasis
ykkC-yxkD	Multidrug resistance transporter	**2**	ykkC, ykkD	10. Miscellaneous
ykkC-yxkD	Urea and agmatine utilization	**9**	ucaA, ucaB, speB, hypA, hypB, amt, uctB, uctP, uctA,	5. Secondary metabolism
ykoK	Magnesium transporters	**4**	mgtE, mgtB, corA, mntH	6. Metal homeostasis
ylbH	Miscellaneous	**2**	ylbH, coaD	10. Miscellaneous
yybP-ykoY	Miscellaneous	**18**	yybP, ykoY, ykoX, atcL, yrzF, COG2119, COG1971, COG3339, COG0530, COG1321, htpX, Cbei_1147, CPF_0680, CBO2022, CPF_0825, yugP, TTC0541, Mvan_0329	10. Miscellaneous

*only genes with annotated functional roles in the RegPrecise database are shown under 'gene name';
number of gene families indicates the total types of different orthologues found
